# Transcriptomic profiling of calcified aortic valves in clonal hematopoiesis of indeterminate potential carriers

**DOI:** 10.1038/s41598-022-24130-8

**Published:** 2022-11-27

**Authors:** Francesco Vieceli Dalla Sega, Domenico Palumbo, Francesca Fortini, Ylenia D’Agostino, Paolo Cimaglia, Luisa Marracino, Paolo Severi, Oriana Strianese, Roberta Tarallo, Giovanni Nassa, Giorgio Giurato, Giovanni Pecoraro, Serena Caglioni, Elisa Mikus, Alberto Albertini, Gianluca Campo, Roberto Ferrari, Paola Rizzo, Alessandro Weisz, Francesca Rizzo

**Affiliations:** 1 Maria Cecilia Hospital, GVM Care and Research, 48033 Cotignola, Italy; 2 Department of Medicine, Surgery and Dentistry ‘Scuola Medica Salernitana’, University of Salerno, 84081 Baronissi, Italy; 3 Clinical Research and Innovation, Clinica Montevergine S.P.A., 83013 Mercogliano, Italy; 4 Medical Genomics Program, AOU ‘SS. Giovanni di Dio e Ruggi d’Aragona’, University of Salerno, Salerno, Italy; 5 Department of Translational Medicine, Laboratory for Technologies of Advanced Therapies (LTTA), University of Ferrara, Ferrara, Italy; 6 Genome Research Center for Health, Campus of Medicine, University of Salerno, Baronissi, SA Italy; 7 Cardiology Unit, Azienda Ospedaliero-Universitaria di Ferrara, University of Ferrara, Ferrara, Italy

**Keywords:** Computational biology and bioinformatics, Cardiology

## Abstract

Clonal hematopoiesis of indeterminate potential (CHIP) is characterized by the presence of clones of mutated blood cells without overt blood diseases. In the last few years, it has emerged that CHIP is associated with atherosclerosis and coronary calcification and that it is an independent determinant of cardiovascular mortality. Recently, CHIP has been found to occur frequently in patients with calcific aortic valve disease (CAVD) and it is associated with a poor prognosis after valve replacement. We assessed the frequency of CHIP by DNA sequencing in the blood cells of 168 CAVD patients undergoing surgical aortic valve replacement or transcatheter aortic valve implantation and investigated the effect of CHIP on 12 months survival. To investigate the pathological process of CAVD in CHIP carriers, we compared by RNA-Seq the aortic valve transcriptome of patients with or without CHIP and non-calcific controls. Transcriptomics data were validated by immunohistochemistry on formalin-embedded aortic valve samples. We confirm that CHIP is common in CAVD patients and that its presence is associated with higher mortality following valve replacement. Additionally, we show, for the first time, that CHIP is often accompanied by a broad cellular and humoral immune response in the explanted aortic valve. Our results suggest that an excessive inflammatory response in CHIP patients may be related to the onset and/or progression of CAVD and point to B cells as possible new effectors of CHIP-induced inflammation.

## Introduction

Somatic mutations accumulate during the lifespan and those that confer selective advance can give rise to clones of mutated cells. When this occurs in hematopoietic stem cells, a relevant ratio of differentiated cells bearing mutations can be found in the blood. The condition characterized by the presence of mutated clones without overt blood pathology has been defined as clonal hematopoiesis of indeterminate potential (CHIP)^1^. Among the most characterized CHIP-driving mutations, there are those affecting genes encoding for epigenetic factors such as DNA methyltransferase 3A (DNMT3A), Tet Methylcytosine Dioxygenase 2 (TET2), and ASXL Transcriptional Regulator 1 (ASXL1) or signaling proteins such as Janus kinase 2 (JAK2)^2^.

Somatic mutations are infrequent in young people but they remarkably increase with age^2,3^.Conventionally, CHIP is defined as the presence of an expanded blood cell clone that presents a mutation in a known driver gene with a variant allele frequency (VAF) of at least 2%, a cutoff mostly imposed by coverage limitation of the applied next-generation sequencing (NGS) technologies^1^. Indeed, this “old” definition is currently overtaken by targeted error-corrected NGS that mitigates systematic errors and can detect even much smaller mutant clones^4^. On the other hand, this cutoff may have biological significance as a mutated clone needs to reach a certain VAF before having an impact on human health^5^.

In recent years, population studies have shown that the presence of CHIP mutations increases the risk of cardiovascular mortality^6^. At the same time, experimental studies in animal models have established the causal role of CHIP mutations in promoting atherosclerosis and in accelerating heart failure^7,8^. CHIP is believed to promote cardiovascular disease by increasing the inflammatory potential of immune cells harboring mutations, as shown by the increase of inflammation circulating markers^9,10^ or specific inflammatory cell subsets^11^. Increased inflammatory potential of CHIP has also been confirmed experimentally in bone-marrow cells with inactivating mutations in TET2 or DNMT3A^7^. More recently, single-cell RNA sequencing of human peripheral blood cells has confirmed that the presence of CHIP mutations promotes an inflammatory phenotype in both monocytes and T cells^12,13^. Although it is now clear that CHIP mutations are able to prime immune cells for an excessive response, the cellular and molecular mechanisms through which CHIP-induced inflammation promotes human cardiovascular diseases remain unknown.

Among cardiovascular diseases, calcific aortic valve disease (CAVD) is a major cause of illness and death^14^. CAVD is characterized by the narrowing of the aortic valve opening and the consequent left ventricular outflow obstruction. The combination of these alterations is responsible for the development of symptoms and the following events that characterize the advanced stages of the disease^15^. To date due to the incomplete understanding of CAVD pathogenesis, no effective medical treatment can prevent or slow the progression of CAVD and aortic valve replacement remains the only clinical treatment for severe, symptomatic aortic stenosis^16^. Recently, it has been found that mutations in TET2 and DNMT3A are frequently present in CAVD patients^11^, and this is linked to an increase in the inflammatory signature of circulating monocytes^12^ and to alterations in the number of circulating pro-inflammatory T cells^11^. On this basis, it has been suggested that CHIP may play a role in promoting CAVD. Nevertheless, no cellular and molecular mechanistic data exist, either from animal models or from human tissues, linking CHIP to the progression of CAVD.

The present study aimed to gain insights into the pathological process of CAVD in CHIP carriers. To this end, we screened for CHIP, by DNA sequencing of blood samples, a cohort of 168 patients with calcified aortic stenosis who had undergone surgical aortic valve replacement (SAVR) or via transcatheter aortic valve implantation (TAVI). Furthermore, we investigated the effect of CHIP on survival 12 months after valve replacement. To uncover molecular pathways that could link CHIP to CAVD, we examined the aortic valve transcriptome from CHIP, non-CHIP patients, or non-calcific controls by RNA-Seq. We then validated our results by immunohistochemistry on formalin-embedded aortic valve samples.

## Results

### Detection of CHIP in patients with calcific aortic stenosis

**Figure 1:**
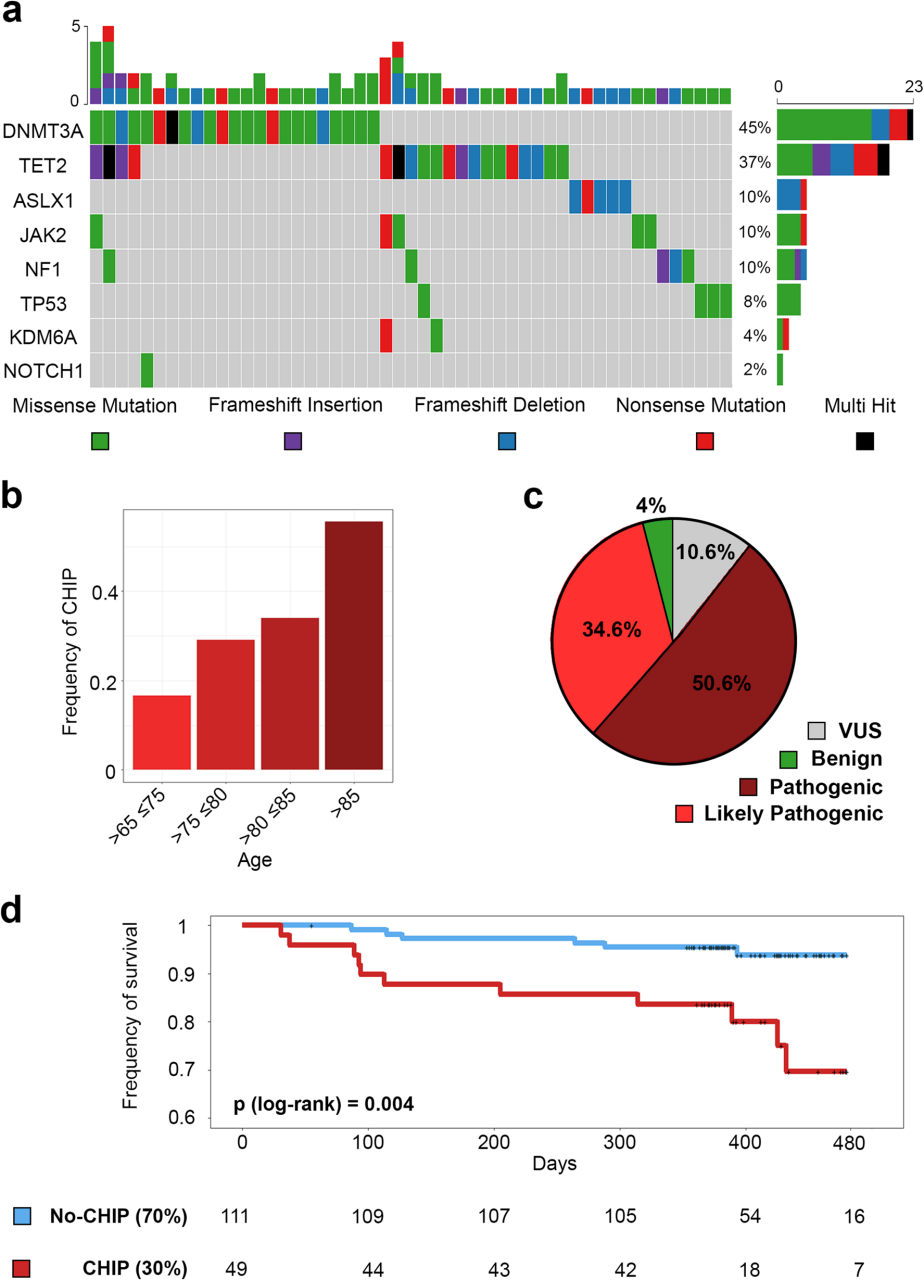
Identified mutations and survival. (**a**) Mutations landscape in the 9 genes. (Top panel) Each column represents per-sample mutations, with a range from a minimum of one to a maximum of 5, (Lower panel) Color-coded matrix of individual mutations, rows show the mutation distributions among the genes. On the right panel is shown the percentage of per-gene mutations. (**b**) Frequency of CHIP in our cohort divided by age ranges. The color gets darker as the frequency increases. (**c**) Pie chart of variants clinical annotation from VarSome. (**d**) Kaplan–Meier cumulative survival curves of CHIP (red) vs No-CHIP (blue). The number of CHIP and No-CHIP patients over the time in our cohort is shown below the plot.

A cohort of 168 patients affected by calcific severe aortic stenosis was analyzed by targeted sequencing using a custom panel of 9 genes, selected among those more frequently mutated in CHIP, namely: DNMT3A, TET2, ASXL1, JAK2, NOTCH1, TP53, KDM6A, CBL, and NF1. An average of 15 million reads per sample were generated, covering the regions of interest at 99.89% with at least 10X coverage and approximately 98% with 50X coverage and with an average depth of 350X (Supplementary Table 1). We were able to find 306 somatic variants distributed among the 9 genes. Furthermore, one-third of the single nucleotide variants corresponds to a C > T substitution, which is usually associated with increased susceptibility to deamination: a clear mutational signature correlated to aging (Supplementary Fig. 1)^2,17,18^. After filtering for not synonymous variants and a variant allele frequency (VAF) higher than 0.02 we obtained 75 mutations associated with CHIP (Fig. 1A and Supplementary Table 2). In accordance with literature^6^, the most frequently affected genes were TET2 (24) and DNMT3A (29) genes, accounting for 32% and 38.6% of the total CHIP-related mutations identified (Fig. 1a).

Overall, we identified 51 out of 168 subjects with CHIP (30.3%), defined as having at least one mutation with VAF > 2%. 16 of the mutated individuals (31.7%) had more than 1 mutation. The majority (12/16) of these had 2 mutations, 1 had 3 mutations, 2 had 4 mutations, and only 1 patient had 5 mutations (Fig. 1a). Respectively, 45% and 37% of all the patients with CHIP have at least one mutation in DNMT3A or TET2, while only 10% have one mutation in ASXL1, JAK2, TP53, KDM6A, NOTCH1, or NF1. No mutations were observed in CBL in our cohort. Interestingly, most ASXL1 and TET2 mutations are frameshift deletions, while for all other genes most are missense mutations (Fig. 1a and Supplementary Fig. 2). The median VAF of CHIP mutations observed in the entire cohort was 8.9% and the distribution of the allele frequencies showed that most of the identified mutations (60%) have a VAF < 10% (Supplementary Fig. 3a). Notably, NF1 shows the highest median VAF followed by DNMT3A and TET2 (Supplementary Fig. 3b).

Consistent with published data, the CHIP prevalence in our severe AV stenosis cohort increases with patient age, from 16.7% (7 out of 42 patients) in the 65–75 years age group, to 29% (16 out of 55) at 75–80 years, to 34% (18 out of 53) at 80–85 years, and reaching 55.5% (10 out of 18) at 85–90 years (Fig. 1b). Moreover, 50.6% of the detected variants associated with CHIP were classified as Pathogenic by VarSome^19^, 34.6% as Likely pathogenic, 10.6% as Variant of Unknown Significance (VUS), and only 4% as Benign (Fig. 1c and Supplementary Table 2).

The frequency of CHIP in the whole cohort was 30.4%, when cases were grouped by the valve replacement procedure, CHIP frequency was 25.9% (29 out of 112) and 39.3%, (22 out of 56), respectively, in SAVR and TAVI patients, reflecting the age differences of the two sub-groups. Importantly, in comparison to published results on unselected populations cohorts^2,20^ the frequency of CHIP among severe aortic valve stenosis patients was higher in all age groups and similar to that previously reported for this disease^11^.

### Clinical characteristics and prognosis after valve replacement

The clinical and echocardiographic characteristics of the patients are summarized in Table 1. As shown, there were no significant differences (threshold *p* value < 0.05) between the CHIP and no-CHIP groups, except for the age, which was higher in the CHIP group (80.9 vs 78.4). Neither risk factors nor the presence of clinically manifest atherosclerotic vascular disease was different. Similarly, at the time of replacement, the severity of aortic stenosis established by aortic mean gradient was similar in CHIP vs no-CHIP patients. Likewise, hematological and laboratory values did not differ between the two groups except for hemoglobin which was slightly lower in CHIP carriers (12.5 vs 13.1 g/dL).

**Table 1:** Baseline characteristics, echocardiographic and laboratory parameters in patients with and without CHIP.

	TOTAL MEAN	TOTAL SD	No-CHIP MEAN (117)	No-CHIP SD	CHIP MEAN (51)	CHIP SD	*p *value
Age (168)	79.18	5.21	78.44	5.33	80.86	4.54	0.007
Weight kg (168)	72.49	12.02	72.91	12.15	71.55	11.78	0.485
Height cm (168)	163.98	8.47	164.31	8.20	163.24	9.11	0.382
BMI kg m2 (168)	26.92	3.87	26.96	3.87	26.84	3.90	0.687
SEX (F-M) (168)	87 (51.7%)–81		57 (48.7%)–60		30 (58.8%)–21		0.246
AVR Type (SAVR-TAVR) (168)	112 (72.6%)–56		83 (70.9%)–34		29 (56.8%)–22		0.101
Hypertension (YES–NO) (168)	139 (82.7%)–29		97 (82.9%)–20		42 (82.3%)–9		1
Dyslipidemia (YES–NO) (168)	117 (69.6%)–51		77 (65.8%)–40		40 (78.4%)–11		0.147
Type 2 Diabetes mellitus (YES–NO) (165)	43 (26%)–122		31 (26.9%)–84		12 (24%)–38		0.692
Atrial fibrillation (YES–NO) (168)	42 (25%)–126		30 (25.6%)–87		12 (23.5%)–39		0.853
Peripheral artery disease (YES–NO) (167)	34 (20.3%)–133		20 (17.2%)–96		14 (27.4%)–37		0.143
COPD (YES–NO) (168)	22 (13%)–146		13 (11.1%)–104		9 (17.6%)–42		0.338
Prior MI (YES–NO) (167)	19 (11.3%)–148		14 (11.9%)–103		5 (10%)–45		0.791
Smoking history (YES–NO) (168)	46 (27.3%)–122		33 (28.2%)–84		13 (25.4%)–38		0.864
Coronary Artery Disease (YES–NO) (168)	72 (42.8%)–96		47 (40.1%)–70		25 (49%)–26		0.312
ANGINA CLASS (> 3 YES–NO) (152)	11 (7.2%)–141		6 (5.6%)–100		5 (10.8%)–41		0.304
NYHA III + IV (YES–NO) (151)	44 (29.1%)–107		28 (26.4%)–78		16 (35.5%)–29		0.330
**Echocardiographic characteristics**							
LV EF (167)	59.15	10.85	59.41	10.90	58.57	10.81	0.414
LV mass g (106)	131.67	111.68	119.36	110.23	165.94	110.46	0.066
Mean aortic gradient mmHg (167)	45.94	12.69	45.75	12.10	46.38	14.10	0.842
Functional AVA cm2 (127)	0.74	0.21	0.75	0.22	0.72	0.17	0.607
Mitral regurgitation (> 3 + YES–NO) (164)	16 (9.7%)–148		8 (7%)–106		8 (16%)–42		0.088
Tricuspid regurgitation (> 3 + YES–NO) (123)	5 (4%)–118		3 (3.5%)–82		2 (5.2%)–36		1
Aortic regurgitation (> 3 + YES–NO) (166)	15 (9%)–151		10 (8.7%)–105		5 (9.8%)–46		1
**Laboratory**							
White blood count (167)	7.66	2.30	7.54	2.29	7.91	2.33	0.160
Neutrophils (167)	5.68	5.78	5.19	2.23	6.80	9.87	0.165
Lymphocytes (167)	1.65	1.26	1.55	0.56	1.89	2.11	0.745
Monocytes (167)	0.66	0.65	0.61	0.22	0.78	1.13	0.924
Eosinophils (167)	0.17	0.18	0.16	0.18	0.18	0.19	0.508
Basophils (167)	0.03	0.05	0.02	0.04	0.05	0.07	0.064
Hemoglobin g dl (168)	12.84	1.58	13.01	1.48	12.46	1.73	**0.014**
Platelets (168)	216.45	71.87	220.84	70.01	206.37	75.72	0.168
Creatinine mg dl (168)	1.07	0.41	1.05	0.43	1.12	0.37	0.100
Crockoft-gault eGFR ml min (> 60 YES–NO) (168)	86 (51.2%)–82		66 (56.4%)–51		20 (39.2%)–31		0.060
Total cholesterol mg dl (165)	163.16	34.10	166.28	35.13	155.56	30.47	0.054
Triglycerides mg dl (165)	113.21	52.45	116.88	56.73	104.25	39.24	0.300
HDL mg dl (163)	51.05	14.55	52.16	15.55	48.40	11.53	0.172
Albumin g dl (158)	4.03	0.36	4.06	0.37	3.96	0.35	0.116
LDL (162)	89.90	27.69	91.41	29.03	86.32	24.11	0.427

Each column displays the mean or the standard deviation (SD) for the continuous variables of both groups (Total), the No-CHIP or the CHIP samples. For dichotomous variables, the total number of each variable and the percentage of the first one are reported. In the last column, the resulting p-value for each No-CHIP vs CHIP test is shown and the significant ones are written in bold.

Furthermore, patients carrying CHIP mutations experienced a significantly worse survival within 12 months from valve replacement (Fig. 1d). Likewise, when a multivariable Cox proportional regression analysis was performed to account for the potential effect of age, sex, or the type of procedure (TAVI or SAVR), CHIP carriers remained independently associated with an increased death rate in the medium-term follow-up (Long-rank = 0.004 and Cox proportional hazards regression model with *p* value = 0.023). Within 30 days after valve replacement, 6 out of 117 (5.1%) non-CHIP carriers and 2 out of 51 CHIP carriers (3.9%) died during the hospitalization. These patients were excluded from the survival analysis to avoid potential confounding effects of intra- or early post-procedure complications causing death. The mortality after discharge from the hospital was attributable in 10 cases (56%) to cardiovascular events and in 8 cases (44%) to non-cardiovascular causes. Interestingly, when considering only CVD events, CHIP carriers still display increased mortality (Supplementary Fig. 4).

Our study included CAVD patients undergoing different valve replacement procedures (SAVR or TAVI), which choice is driven by age, comorbidity, and frailty. In general, TAVI is preferred in older and complex patients. Importantly, also considering the sub-cohort of 112 patients that underwent SAVR, the mortality was considerably lower in patients without CHIP (Supplementary Fig. 5a). In our cohort CHIP was more frequent in females (30 out of 88) than males (21 out of 85), on the contrary, survival analysis revealed that CHIP status is predictive of adverse outcomes in males, but not in females (Supplementary Fig. 5b and c). Clinical data of these sub-groups are reported in Supplementary Table 3–5.

### Transcriptome profiling of the aortic valves of CHIP carriers

**Figure 2:**
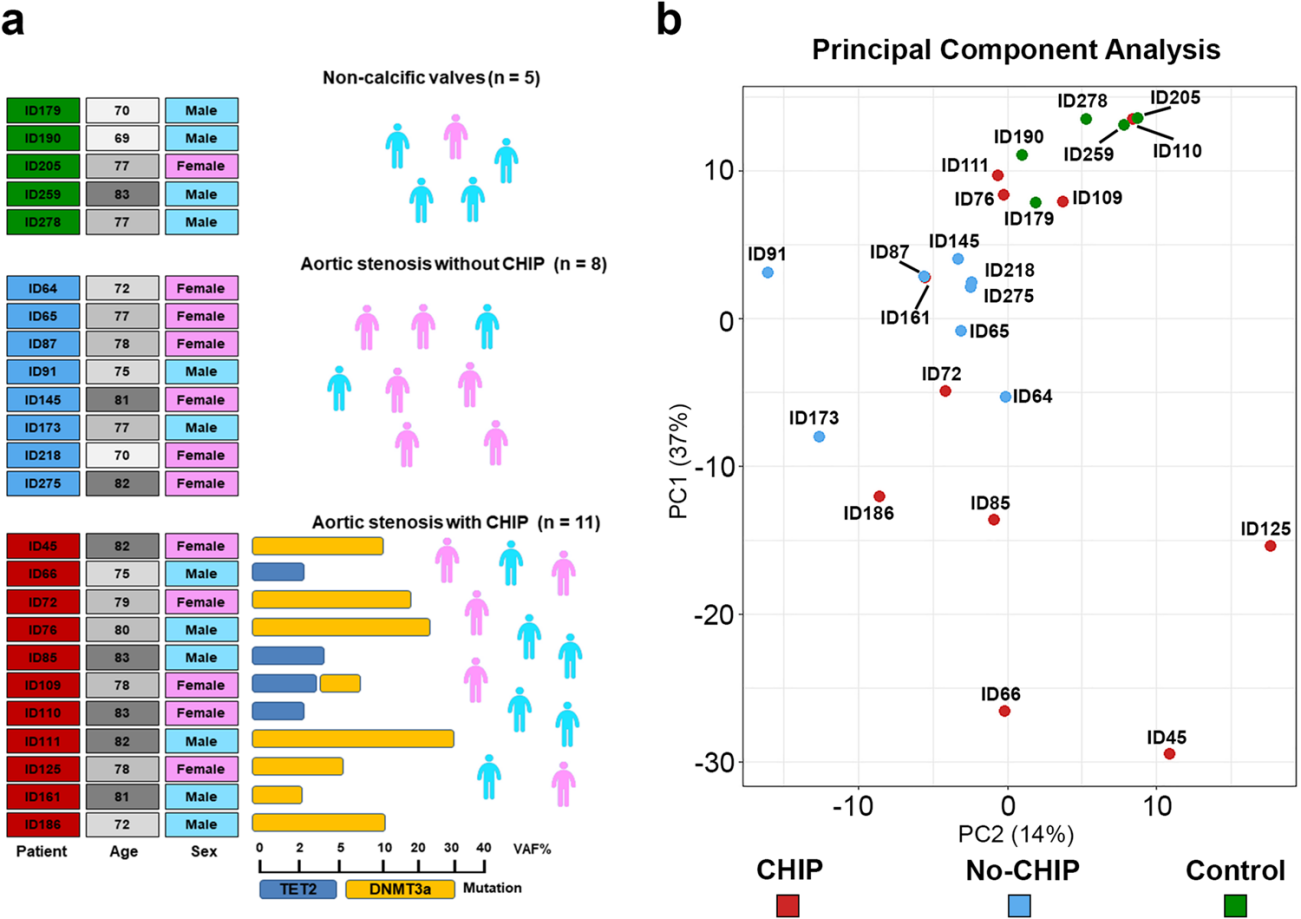
RNA-Seq cohort representation. (**a**) Cohort’s characteristics of RNA-Seq selected patients. The bottom bar shows the percentage of Variant Allele Frequency for the mutations identified in each sample. (**b**) PCA plot of the first two components of the RNA-Seq data. Each group is colored differently.

To gain insights into the molecular mechanism of aortic valve calcification in CHIP patients, we performed RNA-Seq in a subset of aortic valves removed during valve replacement surgery from CAVD patients with (n = 11) or without CHIP (n = 8), and in non-calcified aortic valves from patients without CHIP (n = 5), which we used as non-calcific valve reference (Fig. 2a and Supplementary Table 6). To minimize confounding factors, we included only samples from patients carrying TET2 or DNMT3A mutations, as previous studies demonstrated that monocytes from cardiovascular patients harboring mutations in DNMT3A or TET2 exhibit a highly inflamed transcriptome^12^. As shown in Fig. 2a, among CHIP carriers, 4 patients had TET2 mutation, 5 had DNMT3A mutations, and 1 had both TET2 and DNMT3A mutations. VAF ranged from 2 to 31%. CHIP and no-CHIP patients were similar in age, as well as co-morbidities and severity of stenosis and valve calcification as shown in Table 2 and Supplementary Fig. 6.

**Table 2:** Baseline characteristics, echocardiographic and laboratory parameters in patients with and without CHIP for RNA-Seq analysis.

	TOTAL MEAN	TOTAL SD	No-CHIP MEAN (8)	No-CHIP SD	CHIP MEAN (11)	CHIP SD	*p* value
Age (19)	78.16	3.92	76.50	4.11	79.36	3.47	0.097
Weight kg (19)	69.32	9.68	65.88	9.91	71.82	9.15	0.246
Height cm (19)	163.89	6.93	161.88	8.20	165.36	5.80	0.262
BMI kg m2 (19)	25.79	3.40	25.06	2.90	26.33	3.76	0.386
SEX (F-M) (19)	11 (57.8%)–8		6 (75%)–2		5 (45.4%)–6		0.340
AVR Type (SAVR-TAVR) (19)	19 (100%)–		8 (100%)–		11 (100%)–		NaN
Hypertension (YES–NO) (19)	17 (89.4%)–2		8 (100%)–		9 (81.8%)–2		NaN
Dyslipidemia (YES–NO) (19)	14 (73.6%)–5		6 (75%)–2		8 (72.7%)–3		1
Type 2 Diabetes mellitus (YES–NO) (19)	3 (15.7%)–16		1 (12.5%)–7		2 (18.1%)–9		1
Atrial fibrillation (YES–NO) (19)	1 (5.2%)–18		1 (12.5%)–7		-11 (100%)		NaN
Peripheral artery disease (YES–NO) (19)	3 (15.7%)–16		1 (12.5%)–7		2 (18.1%)–9		1
COPD (YES–NO) (19)	–19 (100%)		− 8 (100%)		− 11 (100%)		NaN
Prior MI (YES–NO) (18)	2 (11.1%)–16		1 (12.5%)–7		1 (10%)-9		1
Smoking history (YES–NO) (19)	7 (36.8%)–12		3 (37.5%)–5		4 (36.3%)-7		1
Coronary Artery Disease (YES–NO) (19)	6 (31.5%)–13		2 (25%)–6		4 (36.3%)-7		0.640
ANGINA CLASS (> 3 YES–NO) (19)	1 (5.2%)–18		− 8 (100%)		1 (9%)-10		NaN
NYHA III + IV (YES–NO) (19)	3 (15.7%)–16		1 (12.5%)-7		2 (18.1%)-9		1
**Echocardiographic characteristics**							
LV EF (19)	62.80	8.68	65.40	8.62	60.90	8.61	0.177
LV mass g (18)	153.83	115.04	154.54	106.86	153.38	125.08	0.928
Mean aortic gradient mmHg (19)	44.68	13.06	43.88	10.43	45.27	15.17	0.804
Functional AVA cm2 (14)	0.73	0.22	0.74	0.35	0.72	0.12	0.787
Mitral regurgitation (> 3 + YES–NO) (19)	2 (10.5%)-17		1 (12.5%)–7		1 (9%)–10		1
Tricuspid regurgitation (> 3 + YES–NO) (18)	− 18 (100%)		− 8 (100%)		− 10 (100%)		NaN
Aortic regurgitation (> 3 + YES–NO) (19)	− 19 (100%)		− 8 (100%)		− 11 (100%)		NaN
**Laboratory**							
White blood count (19)	7.78	1.81	6.88	1.62	8.44	1.71	0.052
Neutrophils (19)	5.40	1.68	4.51	1.70	6.05	1.41	0.090
Lymphocytes (19)	1.59	0.48	1.64	0.35	1.55	0.56	0.707
Monocytes (19)	0.59	0.17	0.56	0.22	0.61	0.14	0.401
Eosinophils (19)	0.17	0.12	0.13	0.05	0.21	0.14	0.089
Basophils (19)	0.05	0.05	0.05	0.05	0.05	0.05	0.623
Hemoglobin g dl (19)	12.81	1.20	13.01	1.53	12.65	0.94	0.649
Platelets (19)	230.47	54.29	240.13	57.13	223.45	53.78	0.741
Creatinine mg dl (19)	0.91	0.33	0.79	0.24	1.01	0.36	0.152
Crockoft-gault eGFR ml min (> 60 YES–NO) (19)	12 (63%)–7		6 (75%)–2		6 (54.5%)–5		0.667
Total cholesterol mg dl (19)	169.53	165.55	175.00	20.89	165.55	26.86	0.492
Triglycerides mg dl (19)	100.21	104.45	94.38	35.86	104.45	23.51	0.492
HDL mg dl (19)	53.11	51.00	56.00	10.54	51.00	16.91	0.247
Albumin g dl (17)	4.19	4.11	4.30	0.12	4.11	0.19	0.042
LDL (19)	96.38	93.65	100.13	23.20	93.65	18.37	0.442

Each column displays the mean or the standard deviation (SD) for the continuous variables of both groups (Total), the No-CHIP or the CHIP samples. For dichotomous variables, the total number of each variable and the percentage of the first one are reported. In the last column, the resulting *p* value for each No-CHIP vs CHIP test is shown.

PCA analysis partially clustered non-calcific versus calcific samples but did not clearly differentiate CHIP from non-CHIP samples. PCA has also highlighted a remarkable heterogeneity in the transcriptomic profile of the calcific samples, particularly of those from patients with CHIP (Fig. 2b).

**Figure 3:**
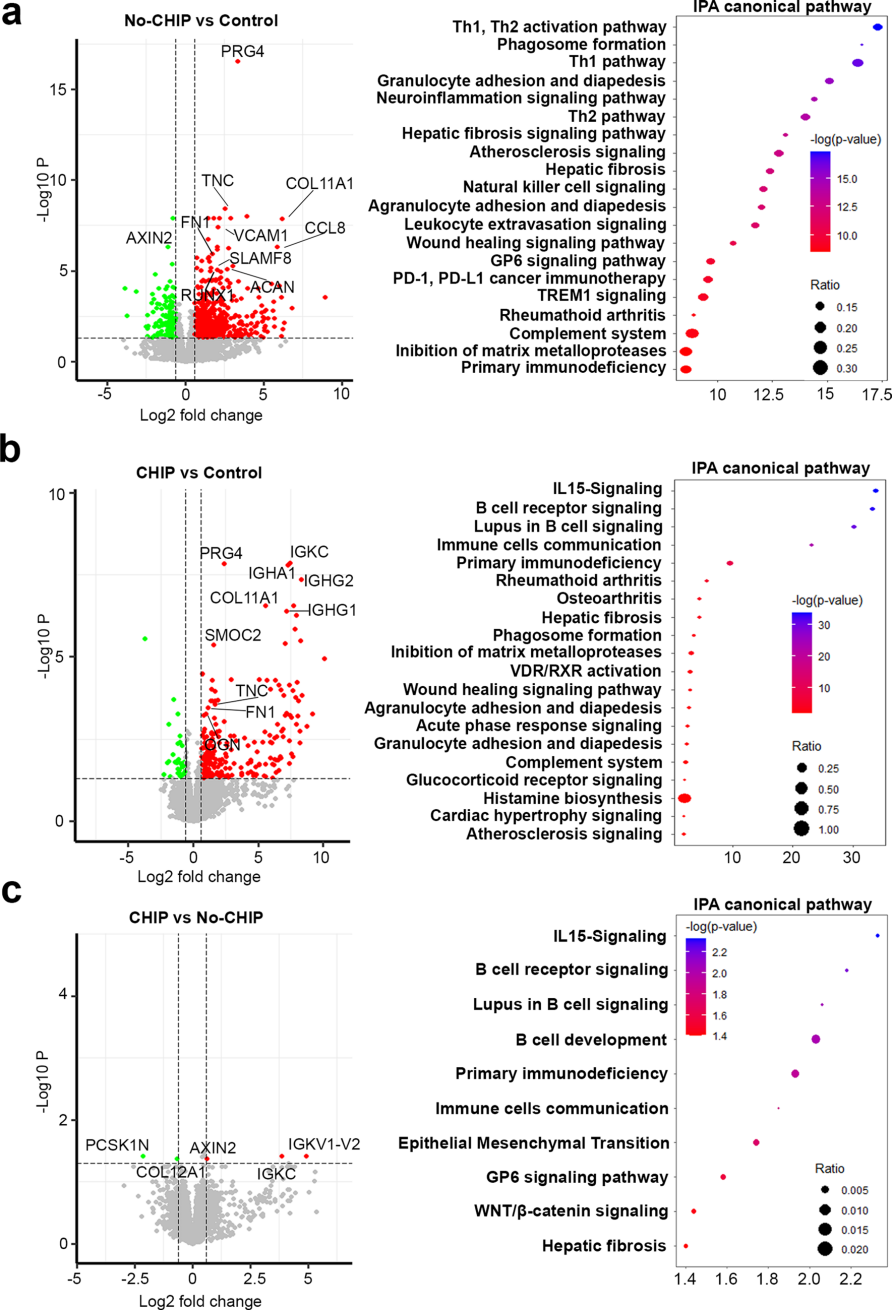
Differentially expressed genes and pathway analysis. (Left panel) Volcano plot of the expressed genes obtained by RNA-Seq analysis and (Right panel) Dot plot of the IPA canonical pathways analysis, showing the ratio (bigger dot size is related to bigger ratio) of each gene set and the -log *p* value (ranging from red to blue), of CHIP vs No-CHIP (**a**), No-CHIP vs Control (**b**) and CHIP vs Control (**c**).

Comparison of CAVD samples of no-CHIP carriers versus non-calcified controls revealed 859 differentially expressed genes (DEGs) (Fig. 3a, left panel and Supplementary Table 7), including several genes regulating biological processes implicated in aortic valve calcification such as immune and inflammatory responses, ossification, and chondrogenic differentiation. Among the top 50 DEGs, many have already been observed in previous RNA-Seq studies using similar samples^21,22^, these include genes related to inflammatory responses such as CCL8, VCAM1, SLAMF8, or related to alterations of extracellular matrix, mineralization, and osteo-chondrogenic differentiation such as PRG4, TNC, FN1, COL11A1, RUNX1, AXIN2, and ACAN.

A comparison of the valves from CHIP patients with non-calcific controls identified 266 DEGs (Fig. 3b, left panel and Supplementary Table 8). Most of these genes are related to adaptive immune responses with 67 genes related to B cell activation and immunoglobulins production, accounting for roughly 25% of the DEGs. Strikingly, of the top 50 DEGs, nearly half (23 genes) are involved in adaptive immune responses, with 17 of them being involved in the humoral response. These genes include those that encode antibody components, comprising constant and variable regions, light and heavy chains of both IgG and IgA such as IGKC, IGHA1, IGHG1, and IGHG2. Among DEGs not related to humoral immunity many overlap with those already implicated in aortic valve calcification, these include TNC, PRG4, COL11A1, SMOC2, FN1, and OGN.

When directly comparing the transcriptome of calcific samples of patients with or without CHIP, only 5 DEGs satisfied the criterion of p < 0.05 adjusted for the false discovery rate (FDR) (Fig. 3c, left panel and Supplementary Table 9), this is probably due to the high heterogeneity in gene expression of calcific samples. Noteworthy, among these genes, immunoglobulin κ constant (IGKC) and IGKV1-12 are involved in antigenic responses, AXIN2 plays a well-known role in vascular calcification, and COL12A1 has been implicated in aortic stenosis and osteo-chondrogenic differentiation^23–25^.

Pathways analysis revealed that CAVD patients without CHIP in comparison with non-calcific controls show disruption in multiple pathways including, Th1, Th2, and NK cell signaling, leukocytes extravasation, phagosome activation, atherosclerosis signaling, and inhibition of matrix metalloproteases (Fig. 3a, right panel). CHIP carriers, in comparison to controls, display alterations that partly overlap those of non-CHIP carriers, such as phagosome activation, atherosclerosis signaling, and inhibition of matrix metalloproteases. In addition, in CHIP carriers, pathways such as IL-15 signaling, B cell receptor signaling, lupus in B cells signaling, and rheumatoid arthritis pathways were dysregulated in comparison to control (Fig. 3b, right panel). Similarly, the direct comparison of CHIP and non-CHIP samples returned alteration of pathways related to IL-15 signaling, B cells development, and lupus in B cells signaling (Fig. 3c, right panel). However, this analysis is based only on 5 DEGs, indicating many similarities with the CHIP comparison to the control samples, and showing, then, only a limited relevance.

### Immune cells and immunoglobulins in calcific aortic valves

**Figure 4:**
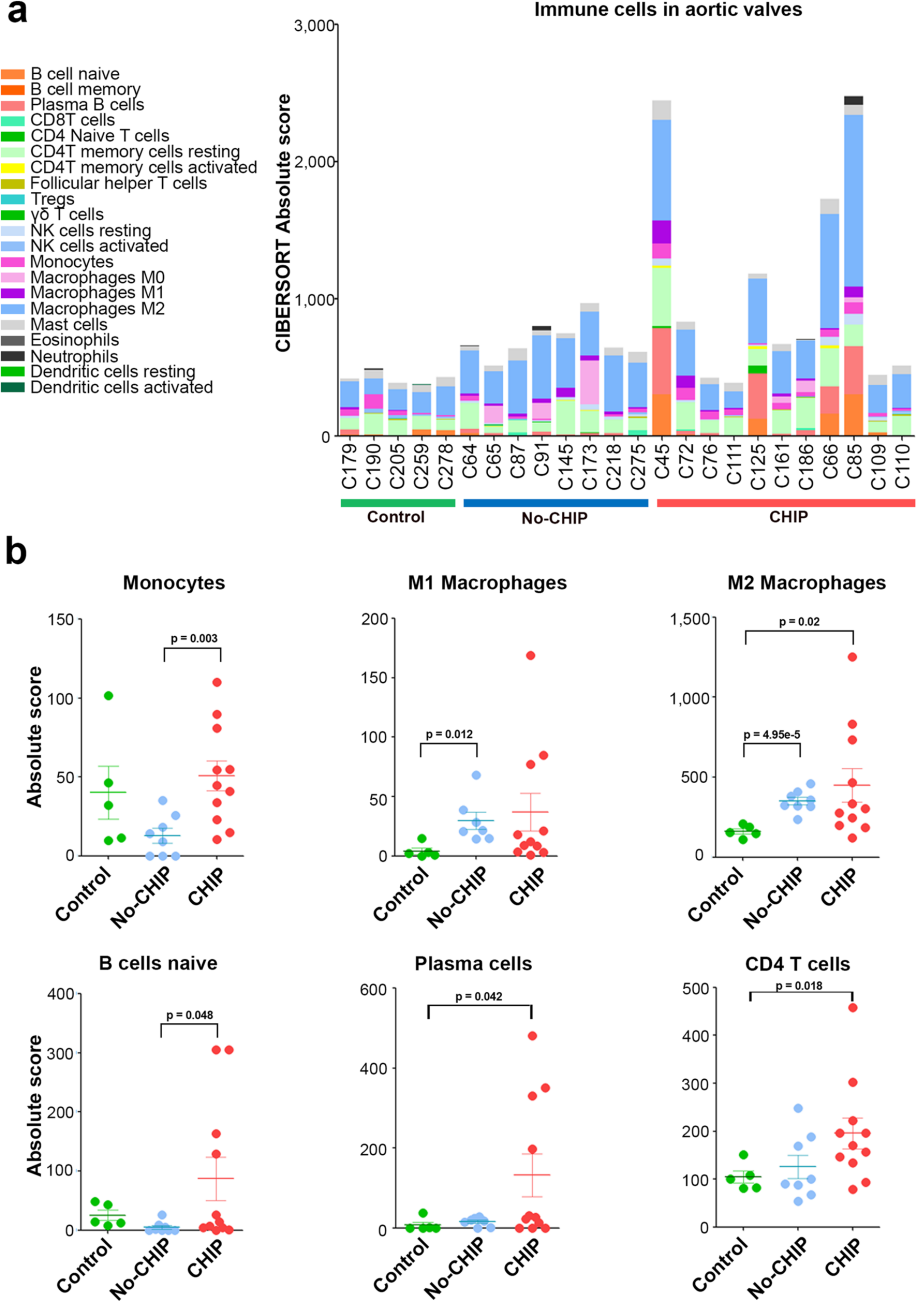
Immune cell profiles in the aortic valves. (**a**) Content of immune cells profile in aortic valve samples obtained by RNA-Seq data. (**b**) Quantification of different immune cell subsets in control, No-CHIP, or CHIP samples. CD4 T cells include memory and naïve T cells. Mean with SEM, are shown. *p* values are referred to T-test comparisons between different groups.

To characterize more in-depth immune cell infiltration in aortic valve samples, we used CIBERSORTx, a deconvolution algorithm that estimates the abundance of 22 immune cell subpopulations starting from bulk RNA-Seq data. The immune cells' relative amounts in the aortic valves of CHIP and non-CHIP CAVD patients, and non-calcific controls, are shown in Fig. 4a. As expected, the calcified samples, from both CHIP and non-CHIP carriers contain more immune cells in comparison to controls. In particular, calcific samples have a higher number of different subtypes of T cells, B cells, and macrophages.

When comparing CAVD samples with or without CHIP, the CHIP ones contain more naïve B cells (*p* = 0.048) and plasma cells, even if, for the latter, the difference is slightly above the limit of significance (*p* = 0.055) (Fig. 4b). Importantly, the amount of infiltrated B cells varies considerably among valves with some samples containing virtually no B cells and others with relatively high levels of cells of the B lineage. Although the presence of B cells was not restricted to CHIP samples, high levels of B cells infiltrate (defined as those exceeding the median of calcific samples) are more prevalent in valves of CHIP patients (7/11) in comparison to non-CHIP (2/8) or controls (1/5). Correlation analysis shows that the number of plasma cells correlates positively with macrophages (M0, M1 and M2 subsets) and CD4 T-cells (naïve and memory) and negatively with NK and Treg cells (Supplementary Fig. 7).

**Figure 5:**
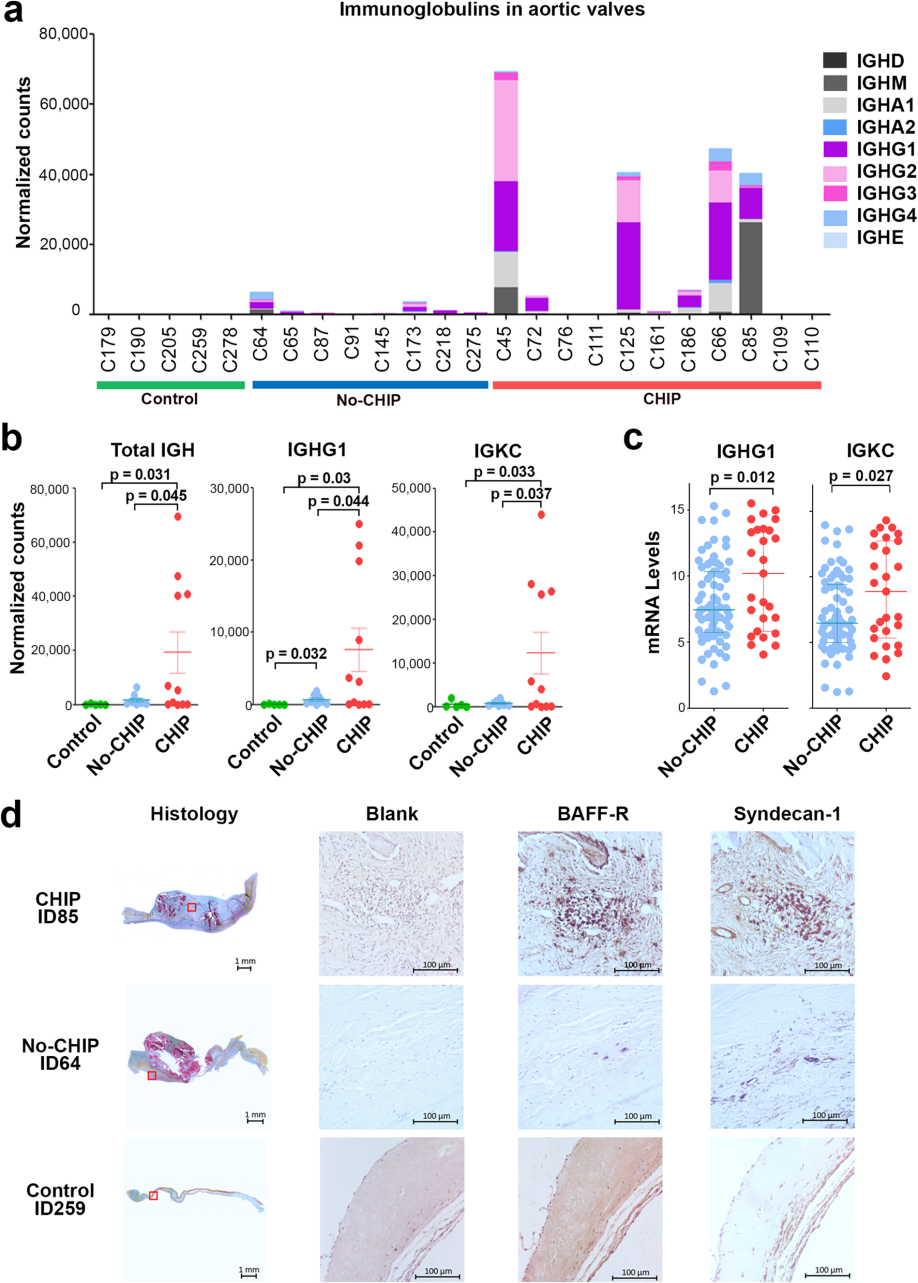
Immunoglobulins analysis and histological images of the aortic valves. (**a**) Content of immunoglobulins-related transcripts in aortic valve samples. (**b**) Quantification by RNA-Seq of immunoglobulins-related transcripts in control, no-CHIP, or CHIP samples. Lines indicate the mean with SEM, *p* values are referred to T-test comparisons between different groups. (**c**) Quantification by qRT-PCR of representative immunoglobulins transcripts in RNA extracted from No-CHIP and CHIP samples. (**d**) Representative images of aortic valves of controls, No-CHIP, CHIP samples. Histological staining on the right shows the entire cusp after Russell-Movat pentachrome staining identifying elastin fibers (black), collagens (yellow), proteoglycans (blue), muscle (red), and cell nuclei (purple). Red squares indicate the areas corresponding to immunohistochemistry images. BAFF-R or CD138 (Syndecan-1) antibodies were used to stain activated B cells or plasma cells, respectively. Negative control sections (Blank) underwent the same procedure except for the primary antibody, which was omitted.

We have also estimated the levels of antibodies in the different samples using the expression of a panel of immunoglobulin-related genes. As expected, data mirrored those of plasma cells calculated by deconvolution confirming that samples with high B cells infiltration also contain more transcripts encoding antibodies as exemplified by IGKC, IGHG1 and total IGH (Fig. 5a–b). To assess if these genes are expressed in a secreted or membrane-associated form, we investigated alternative splicing of the IGHM gene. We observed that the exons coding for the membrane-anchoring portions were expressed at lower levels compared to other exons (Supplementary Fig. 8). This suggests that the majority of transcripts encode for antibodies that can be secreted, while only a fraction of them encode for splicing variants containing membrane-anchoring portions belonging to B cell receptors.

To validate this observation in a larger number of samples we measured by qRT-PCR the levels of transcripts of two representative immunoglobulins genes, namely IGKC and IGHG1, in the RNAs extracted from 102 aortic valves collected from patients undergoing SAVR. In this way, we confirmed that aortic valves of CHIP patients contain higher levels of transcripts for immunoglobulins compared to those without CHIP (Fig. 5c).

To confirm the presence of antibody-producing cells we performed immunohistochemistry of aortic valve- formalin-fixed samples, matching those used for RNA-Seq. We used BAFF-R or CD138 (Syndecan-1) as markers of activated B cells or plasma cells, respectively^26^.We confirmed that all the samples containing high levels of immunoglobulins transcripts display obvious B cells/plasma cells infiltration, representative images are shown in Fig. 5c. In contrast, we found only sporadic or no B cells in samples containing little or no immunoglobulins transcripts. Interestingly, in the valves expressing high levels of immunoglobulins, BAFF-R positive cells were found in clusters containing also CD138-positive cells often localized near the calcific lesions (Fig. 5d). Finally, we quantified BAFF-R and syndecan-1 positive cells in histological samples. We found an increase of B cells in CHIP samples and a similar trend with CD138 positive cells, although T-test was slightly above significance (*p* = 0.08) (Supplementary Fig. 9). Importantly, this data mirrored those of CIBERSORT estimation, further validating this type of analysis.

### Association between immunoglobulins in the valve and mortality

**Figure 6:**
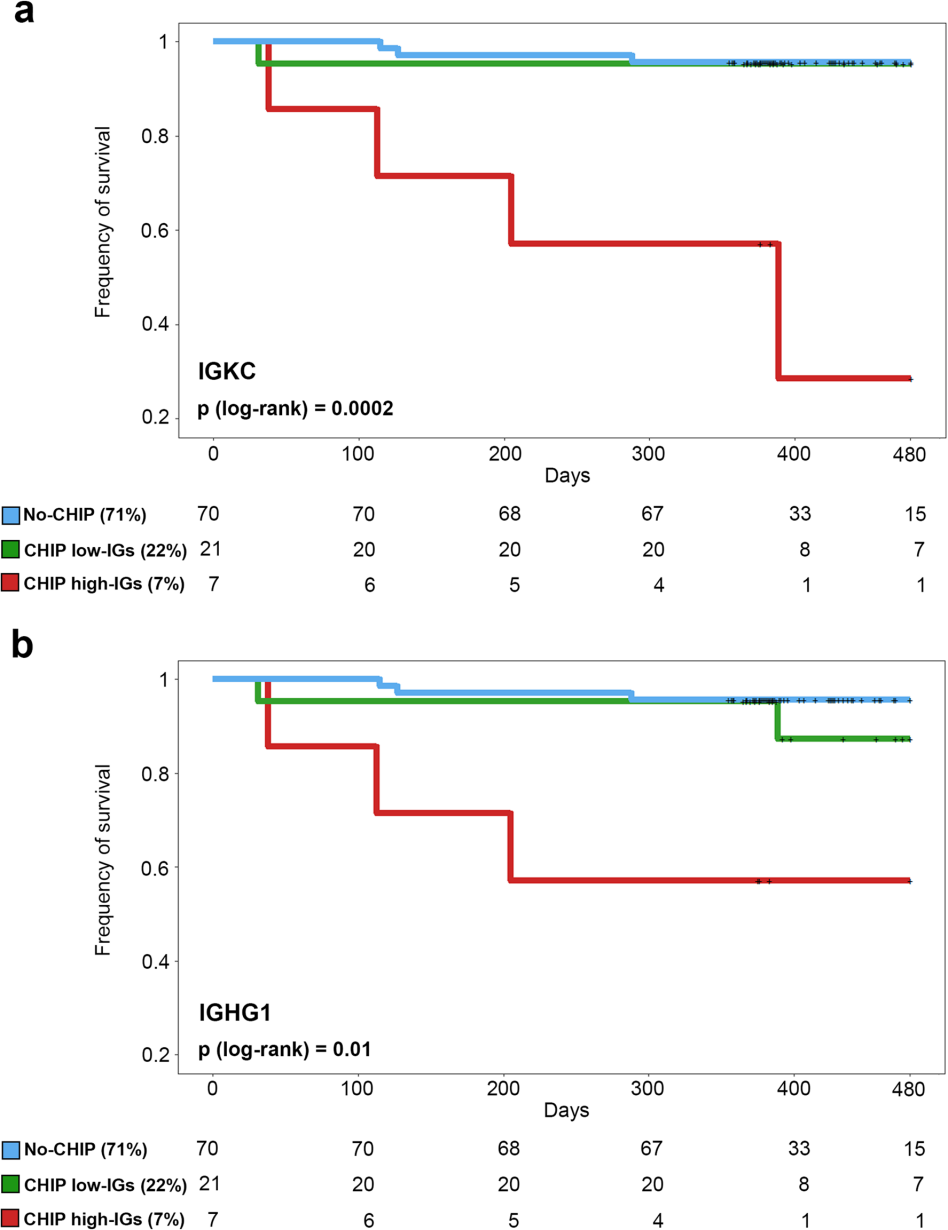
Association between survival and immunoglobulins expression in the aortic valves. Kaplan–Meier cumulative survival curves of No-CHIP (blue), CHIP carrying high levels (top quartile) of immunoglobulins IGKC (panel **a**) or IGHG1 (panel **b**) transcripts (red), or CHIP carrying low IGs (bottom 3 quartiles) (green). The number of patients over the time for the different cohorts is reported below each plot.

Finally, we investigated whether the presence of immunoglobulins in the aortic valves of CHIP patients is related to mortality. We found that patients with CHIP and high levels of IGKC or IGHG1 (top quartile) have poor survival in comparison to those with CHIP but with low levels of immunoglobulins (3 bottom quartiles), which have a survival rate similar to those without CHIP (Fig. 6). Cox-regression analysis showed that the increased mortality was independent of sex and age (*p* = 0.0002 and *p* = 0.01 for IGKC and IGHG1, respectively).

## Discussion

In this study, we compared the aortic valve transcriptome of CAVD patients in the presence or absence of CHIP mutations. We found a high heterogeneity in the trascriptome of CHIP carriers’ valves, which resulted in limited differences between samples with or without CHIP. Despite these limited differences, we found that CHIP patients frequently display a broad cellular and humoral immune response in the aortic valve. Our findings are in line with recent evidence showing that CHIP mutations stimulate immune cells leading to an excessive inflammatory response that increases the cardiovascular risk^13^ and points to B cells as possible players in CHIP-induced inflammation.

By analyzing the transcriptome of aortic valves of CAVD patients, we identified altered expression of several genes which are shared by patients with or without CHIP, these include genes already known for their role in CAVD and related to the extracellular matrix and osteo-chondrocyte transition such as TNC, PRG4, COL11A1, SMOC2, FN1, and OGN^21,27,28^. In agreement with previous reports, pathways analysis has revealed alterations in innate and adaptive immune responses, phagosome activation, atherosclerosis signaling, and inhibition of matrix metalloproteases^22,29,30^. RNA-Seq of diseased valves from patients harboring TET2 or DNMT3A mutations showed a more profound humoral immunity involvement as indicated by higher levels of transcripts related to B cells and immunoglobulin production in comparison to non-calcified valves, or non-CHIP valves. This was also confirmed in a larger set of aortic valves in which we observed higher transcript expression of representative immunoglobulins IGKC and IGHG1 in CHIP carriers than in non-CHIP patients. Additionally, when comparing valve transcriptomes of CHIP patients to non-calcific controls, several pathways related to B cell functions and autoimmune diseases such as systemic lupus erythematosus and rheumatoid arthritis emerged. In addition, IL-15 signaling was altered in the valves of CHIP carriers in comparison to controls, this is consistent with the role of IL-15 in the interactions between macrophages, T cells, and B cells^31,32^. Consistently with previous studies reporting that CHIP-associated mutations affect circulating immune cells, including monocytes, neutrophils, and T cells^33^, we found that end-stage aortic valves of CHIP patients compared to non-CHIP carriers contain more monocytes, B cells, and plasma cells. Noteworthy, the number of B cells has been associated with the severity of the stenosis, suggesting a negative impact of these cells on the valvular disease progression^26^. We also observed that valves from CHIP carriers also have more T cells and macrophages, although the difference was not statistically significant, likely due to the high heterogeneity and relatively low number of samples. Importantly, plasma cell number is positively correlated with macrophages and CD4 T cells, but negatively with immunomodulatory Tregs. This is in line with data showing an increased circulating Th17/Treg ratio in CHIP carriers with CAVD^11^.

Overall, our results indicate that CAVD in CHIP carriers occurs through the typical valvular alterations of the disease but also highlight an augmented involvement of adaptive immune activation and frequently an involvement of humoral responses. Our findings are consistent with recent data showing that, in the context of CHIP, the cooperation of innate and adaptive responses drives an aberrant inflammatory response^13^, and with the concept that different immune cell subtypes participate in the inflammatory response within the calcified aortic valves^34,35^.

The aortic valve calcification is a multi-step process occurring within years, meaning that at the time of surgery the valves have already been profoundly altered. The phenotype of end-stage calcific aortic valves is the result of several layers of inflammatory damages that occurred during the long way leading to the symptomatic disease. In this scenario, it seems plausible that the increase of immune potential in monocytes and T cells in CHIP carriers^13^ facilitates a chronic inflammatory state that may then attract humoral immunity. Moreover, CHIP mutations also occur in B cells^1,36^ and a more direct role of mutated B cells also appears possible^37^. B cells are not normally present in healthy cardiac valves but accumulate in calcific aortic valve tissue^26,38,39^. B cells can be activated by toll-like receptor (TLR) signaling or macrophage-secreted cytokines, such as B cell-activating factor (BAFF) which promotes their proliferation. On this basis, it has been proposed that B cell accumulation may underlie a progressive cycle of pro-inflammatory antigen presentation and production of pro-inflammatory markers linking B cells to CAVD^26^. Our data are in agreement with this model, however, future works will be necessary to assess whether the increased activation of humoral immunity only reflects the increased inflammation of subjects with CHIP or if there is a causal link to the pathological calcification.

However, it must be considered that calcific aortic valves that we analyzed display a remarkable heterogeneity. This is not unexpected, as several studies have shown that the phenotype of the stenotic valve can vary greatly as well as the extent and the characteristics of immune infiltration^38,39^. In fact, we have observed two groups of CHIP carriers, one with higher immunoglobulin levels and the other showing comparable levels to non-CHIP indicating that the presence of immunoglobulins in CAVD is not a peculiar of CHIP. This is not surprising as many reasons can explain this scenario: (1) The activation of humoral immunity can be associated with other conditions, not necessarily with CHIP; (2) Different CHIP mutations in immune cells can have divergent effects on the inflammation; (3) Even in the case of mutations promoting inflammation, their effect on CAVD pathogenesis depends on several other exogenous or endogenous individual factors; (4) Some patients with CHIP may not have been recognized as they may be carriers of CHIP mutations on genes not tested in this study.

Before our study, another work reported that CHIP mutations in TET2 and DNMT3A occur frequently in patients with CAVD and that CHIP presence at the valve replacement time determines a poor prognosis after successful TAVI^11^. Overall, our data are comparable to this report, which has found a CHIP frequency of 33.3% in TAVI patients, a frequency similar to the one observed in our whole cohort (30.4%, rising to 39% when considering only TAVI patients). Similarly to what Mas-Peiro et al. reported in TAVI patients, here, we found that all-cause mortality after 12 months was higher in CHIP compared to non-CHIP carriers. Interestingly, this remains valid also when considering only mortality due to cardiovascular events (Long-rank = 0.04). However, there are relevant differences between the two studies. Our cohort included most patients eligible for cardiac surgery that are on average younger than TAVI patients, as a result, the average age of our cohort was 79.2, compared to 83.0 of the cohort studied by Mas-Peiro and colleagues. Importantly, in our study, the impact of CHIP on prognosis was confirmed also in the subgroup of 112 SAVR patients. Moreover, we detected CHIP using a panel of 9 genes while Mas-Peiro et al. used only TET2/DNMT3A, explaining the higher frequency of CHIP in TAVI patients. Of note, in our cohort, the impact of CHIP on prognosis seems to be more pronounced in males compared to females. However, we cannot rule out that this may be due to decreased size and hence, the lower statistical power of this sub-analysis.

Importantly, we found that among CHIP carriers, those characterized by high levels of immunoglobulins in their valves display increased mortality in comparison to other CHIP patients. This may indicate that the broad cellular and humoral immune response in the aortic valves of CHIP patients reflects deep alterations in the immune cells that may be linked to the poor prognosis. However, as we evaluated overall mortality it is important to highlight that this observation does not imply that the increased mortality is directly linked to CAVD.

Some limitations of this study must be acknowledged. Firstly, we identified alterations in the calcific valves of CHIP carriers that may link CHIP to CAVD, however our study is mostly descriptive, and thus we cannot infer causality between the phenotype and the disease. Secondly, we necessarily had to examine aortic valves from patients with end-stage disease: while this can provide hints of prior inflammatory damages, it precludes the possibility to obtain direct insights on the onset and progression of CAVD. Moreover, the RNA-Seq analysis included a relatively low number of patients although comparable with similar studies^21,40^. Finally, it should be considered that, as we determined CHIP in a panel of frequently mutated genes, we may have missed some mutations in other genes. In addition, each mutation can have a divergent impact on the functionality of encoded protein and in turn on cellular biology, and the design of our study precluded an individual evaluation of each mutation. For these reasons, the findings of this work must be further investigated in future studies addressing the above discussed limitations.

## Conclusions

The discovery of the possible involvement of inflammation and humoral immunity observed in a relevant fraction of CHIP subjects with CAVD may open new perspectives for these patients. Indeed this inflammatory response resembles that involved in auto-immune diseases such as rheumatoid arthritis and systemic lupus erythematosus, pathologies for which drugs targeting B cells such as rituximab, abatacept, and belimumab are already used in clinical practice, and several others are under assessment in clinical trials^41^. Intriguingly, findings from small clinical trials and animal models have shown that rituximab and abatacept appear to relieve atherosclerosis and heart failure^42,43^. Immunotherapy has already revolutionized the treatment of cancer and autoimmunity, and following the success of CANTOS^44^, inflammation targeting may become a realistic goal in the treatment of CAVD as well.

However, after our study, several crucial questions remain unanswered; studies using animal models will be necessary to confirm the causative role of B cells in promoting CAVD in CHIP carriers. Furthermore, scRNA-seq experiments and B cell receptor sequencing could provide important information on mechanisms and identify the immunological target of immunoglobulins.

Advances in the understanding the CHIP consequences in cardiovascular disease, along with the advent of high-throughput genome sequencing approaches are paving the way for clinical use of CHIP in the individual risk assessment that in the near future may lead to personalized immunotherapy for cardiovascular diseases.

## Methods

### Study population

We studied 168 consecutive patients aged ≥ 65 from March 2018 to March 2020 undergoing valve replacement for calcific severe aortic stenosis at Maria Cecilia Hospital, Cotignola (RA), Italy. Of these, 112 patients had cardiac surgery while 56 patients had TAVI. Rheumatic valve disease, infective endocarditis, end-stage kidney disease, and hematological disorders were exclusion criteria. Clinical, echocardiographic, and laboratory data were prospectively collected. Survival was assessed at 12 ± 2 months follow-up after valve replacement for all 168 patients. In addition, we collected 5 non-calcified aortic valves, as controls, from patients without aortic stenosis undergoing valve replacement for severe aortic insufficiency or aneurysm of the aortic root. The primary aim of this study was to obtain information on the biology of CHIP in CAVD patients by analyzing surgically removed valves. The study was approved by the Ethics Committee of “Romagna” (approval code: 590/2017) and was conducted according to the Declaration of Helsinki. All patients gave written informed consent.

### Biological samples collection

Aortic valve leaflets removed during surgery were immediately immersed in RNAlater (ThermoFisher Scientific, Waltham, MA, USA). A cusp of aortic valves was immediately fixed in 10% neutral buffered formalin for 24 h and then embedded in paraffin for histologic evaluations. Blood samplings were performed from an antecubital vein using a 21-gauge needle or from the central venous line. Blood was collected in the early morning at least 12 h after the last administration of anticoagulant drugs. The first 2 to 4 mL of blood were discarded, and the remaining was collected in EDTA tubes and stored at − 80 °C.

### Detection of CHIP

DNA was extracted from whole blood samples with DNeasy Blood & Tissue Kit (Qiagen, Hilden, Germany), according to the manufacturer's instructions. After genomic DNA (gDNA) extraction, quality control checks were performed using Qubit dsDNA BR Assay Kit (ThermoFisher Scientific, Waltham, MA, USA) and Agilent 2200 TapeStation System (Agilent Technologies, Santa Clara, CA, USA). Libraries were prepared using SureSelectXT Low Input Target Enrichment System for Illumina Paired-End Multiplexed Sequencing Library protocol (Agilent Technologies, Santa Clara, CA, USA). The pool was sequenced at a loading concentration of 1.8 pM on Illumina NextSeq-500 System in a 2 × 150 bp paired-end reads format. For further details on library preparation, sequencing, and bioinformatics analyses see Supplementary Methods.

### RNA sequencing and RT-PCR of aortic valves

Aortic valve leaflets were disrupted and homogenized with the TissueRuptor® (Qiagen, Hilden, Germany). Total RNA was extracted with RNeasy® Fibrous Tissue Mini Kit (Qiagen, Hilden, Germany) according to the manufacturer's instructions. The RNA quality checks were performed using the Qubit RNA HS Assay Kit (ThermoFisher Scientific, Waltham, MA, USA) while RIN (RNA Integrity Number) was determined at Agilent 2200 TapeStation System (Agilent Technologies, Santa Clara, CA, USA). Indexed libraries were prepared starting from 150 ng of purified RNA with TruSeq Stranded Total RNA Library Prep Kit (Illumina, San Diego, CA, USA) according to the manufacturer’s instructions. The pooled samples were sequenced on NovaSeq 6000 System (Illumina, San Diego, CA, USA) in a 2 × 100 paired-end format at a final concentration of 250 pM. For further details on library preparation, sequencing, and bioinformatics analyses see Supplementary Methods. CIBERSORTx is a tool that imputes gene expression profiles and provides an estimation of the abundances of cell types in a mixed cell population, using gene expression data. It employs linear support vector regression (SVR) to deconvolute a mixture of gene expression^45^. In this study, we set CIBERSORTx in absolute mode with 1,000 permutations on our bulk RNASeq data in combination with a curated signature matrix for reference cell types (LM22—22 immune cell types) to calculate and predict the absolute amount of cells type in our samples.

### Histology

For histology and immunostaining, 8 µm sections were cut using a microtome (SLEE medial, Mainz, Germany). Russell-Movat pentachrome (Abcam, Cambridge, UK) or Alizarin Red staining (Sigma Aldrich, St. Louis, MO, USA) were performed according to the manufacturer’s instructions. For immunohistochemistry analysis, after antigen retrieval with EDTA buffer (Sigma-Aldrich, St. Louis, MO, USA), slices were incubated overnight with an anti-BAFF-R or CD138 antibodies (Abcam, Cambridge, UK) diluted at 1:250 or 1:8,000, respectively. Slides were stained with HRP/DAB detection kit (Abcam, Cambridge, UK) and counterstained with hematoxylin to visualize cell nuclei. Images of entire aortic valve cusps were composed using NIS-Elements Microscope Imaging Software (Nikon, Tokyo, Japan) from a series of adjacent pictures taken with a 4 × objective taken with a Nikon Eclipse Ni microscope (Nikon, Tokyo, Japan). Immunostaining images were taken with a 20 × objective with a Nikon Eclipse Ni microscope (Nikon, Tokyo, Japan).

### Statistics

All calculations, statistical analyses, and survival curves were produced on R (v4.0.2) with an alpha value set for *p* < 0.05. Student’s T, Mann–Whitney U, and Chi-squared tests were used to determine the presence of significant differences for continuous and discrete variables. Missing data were excluded during statistical hypothesis testing. For more details see Supplementary Methods.

## Supplementary Information

Supplementary Information.

## Data Availability

DNA-Seq and RNA-Seq data from this study are available in the ArrayExpress archive at https://www.ebi.ac.uk/arrayexpress/ and can be accessed with accession numbers E-MTAB-11352 and E-MTAB-11354.

## Abbreviations

ASXL1: ASXL transcriptional regulator 1
BAFF: B cell-activating factor
CAVD: Calcific aortic valve disease
CHIP: Clonal hematopoiesis of indeterminate potential
DEGs: Differentially expressed genes
DNMT3A: DNA methyltransferase 3A
FDR: False discovery rate
IGKC: Immunoglobulin κ constant
JAK2: Janus kinase 2
NGS: Next-generation sequencing
PCA: Principal component analysis
SAVR: Surgical aortic valve replacement
TAVI: Transcatheter aortic valve implantation
TET2: TET methylcytosine dioxygenase 2
TLR: Toll like receptor
VAF: Variant allele frequency
VUS: Variant of unknown significance
