# Supplementary material for: Transcriptomic profiling of calcified aortic valves in clonal hematopoiesis of indeterminate potential carriers
Source: Sci Rep. 2022 Nov 27;12:20400. doi: 10.1038/s41598-022-24130-8 (PMC9701688; doi:10.1038/s41598-022-24130-8)
Supplement: Supplementary file 1 — Supplementary Information. [file 41598_2022_24130_MOESM1_ESM.docx]

**Transcriptomic profiling of calcified aortic valves in clonal hematopoiesis of indeterminate potential carriers**

Francesco Vieceli Dalla Sega*^1^, Domenico Palumbo*^2,3^, Francesca Fortini^1^, Ylenia D’Agostino^2,4^, Paolo Cimaglia^1^, Luisa Marracino^5^, Paolo Severi^5^, Oriana Strianese^3,6^, Roberta Tarallo^2,6^, Giovanni Nassa^2,6^, Giorgio Giurato^2,6^, Giovanni Pecoraro^2,6^, Serena Caglioni^7^, Elisa Mikus^1^, Alberto Albertini^1^, Gianluca Campo^7^, Roberto Ferrari^1,5^, Paola Rizzo^1,5^° Alessandro Weisz^2,4,6^°, Francesca Rizzo^2,6^°

*The first two authors contributed equally to the study

°corresponding authors

^1^Maria Cecilia Hospital, GVM Care and Research, Cotignola, Italy

^2^Department of Medicine, Surgery and Dentistry ‘Scuola Medica Salernitana’, University of Salerno, 84081 Baronissi

^3^Clinical Research and Innovation, Clinica Montevergine S.p.A., 83013 Mercogliano, Italy;

^4^Medical Genomics Program, AOU ‘SS. Giovanni di Dio e Ruggi d’Aragona’, University of Salerno, Salerno, Italy;

^5^Department of Translational Medicine, Laboratory for Technologies of Advanced Therapies (LTTA),

University of Ferrara, Ferrara, Italy;

^6^Genome Research Center for Health, Campus of Medicine, University of Salerno, Baronissi (SA), Italy;

^7^Cardiology Unit, Azienda Ospedaliero-Universitaria di Ferrara, University of Ferrara, Ferrara, Italy

Corresponding Authors:

Francesca Rizzo

+39 089 96-5221

[frizzo@unisa.it](mailto:frizzo@unisa.it)

Paola Rizzo

+39 0532 455508‬‬‬‬‬‬‬‬‬‬‬‬‬‬‬‬‬‬‬‬‬‬

[rzzpla@unife.it](mailto:rzzpla@unife.it)

Alessandro Weisz

+39 089 96-5043

aweisz@unisa.it

**Supplementary Tables**

| Supplementary Table 1: DNA sequencing statistics | | |
| --- | --- | --- |
| Sample | Passing Filter reads | Average coverage |
| ID1 | 11,309,492 | 473.40 |
| ID2 | 16,138,345 | 622.34 |
| ID3 | 25,448,580 | 522.62 |
| ID4 | 29,489,091 | 751.05 |
| ID5 | 32,869,979 | 605.41 |
| ID6 | 27,550,622 | 661.37 |
| ID7 | 19,365,368 | 645.48 |
| ID8 | 24,629,931 | 1,035.26 |
| ID9 | 29,607,480 | 590.11 |
| ID10 | 18,602,600 | 444.18 |
| ID11 | 16,435,155 | 747.40 |
| ID12 | 15,874,855 | 661.86 |
| ID13 | 18,809,965 | 593.97 |
| ID14 | 20,884,547 | 857.64 |
| ID15 | 24,196,481 | 577.83 |
| ID17 | 16,952,017 | 980.23 |
| ID18 | 17,659,021 | 654.20 |
| ID19 | 16,828,720 | 660.11 |
| ID20 | 11,953,207 | 691.67 |
| ID21 | 22,041,961 | 724.78 |
| ID22 | 18,315,176 | 796.57 |
| ID23 | 20,109,824 | 478.09 |
| ID24 | 12,969,751 | 608.33 |
| ID25 | 15,689,390 | 768.99 |
| ID26 | 16,115,376 | 715.36 |
| ID27 | 17,426,417 | 510.32 |
| ID28 | 19,342,429 | 560.69 |
| ID29 | 16,810,034 | 649.40 |
| ID30 | 20,564,262 | 566.45 |
| ID31 | 20,131,436 | 514.49 |
| ID32 | 16,692,118 | 362.39 |
| ID33 | 19,584,613 | 425.51 |
| ID34 | 19,784,835 | 926.02 |
| ID35 | 19,998,719 | 894.64 |
| ID36 | 21,276,003 | 461.17 |
| ID37 | 17,238,353 | 721.42 |
| ID38 | 14,886,009 | 712.41 |
| ID39 | 26,333,018 | 624.51 |
| ID40 | 23,035,976 | 892.38 |
| ID42 | 22,636,086 | 472.86 |
| ID43 | 24,754,664 | 475.23 |
| ID44 | 22,346,689 | 591.78 |
| ID45 | 18,108,284 | 508.71 |
| ID46 | 21,989,564 | 568.33 |
| ID47 | 22,312,848 | 580.03 |
| ID48 | 12,330,455 | 424.35 |
| ID49 | 18,514,353 | 411.00 |
| ID50 | 18,924,756 | 415.88 |
| ID51 | 19,616,917 | 445.49 |
| ID52 | 20,201,051 | 430.50 |
| ID53 | 17,116,373 | 476.77 |
| ID54 | 13,682,295 | 581.16 |
| ID55 | 13,279,323 | 527.31 |
| ID56 | 15,678,861 | 743.69 |
| ID57 | 15,152,109 | 516.73 |
| ID58 | 15,786,398 | 617.58 |
| ID59 | 16,440,482 | 659.03 |
| ID60 | 19,008,779 | 741.25 |
| ID61 | 11,759,787 | 514.33 |
| ID62 | 17,601,659 | 625.27 |
| ID63 | 14,714,852 | 540.26 |
| ID64 | 16,164,284 | 963.04 |
| ID65 | 23,208,677 | 880.35 |
| ID66 | 20,588,101 | 935.29 |
| ID68 | 17,026,922 | 675.05 |
| ID69 | 26,498,342 | 1,123.84 |
| ID70 | 15,470,598 | 660.02 |
| ID71 | 15,690,070 | 690.24 |
| ID72 | 18,511,238 | 785.44 |
| ID73 | 16,797,319 | 830.34 |
| ID74 | 12,186,180 | 476.44 |
| ID75 | 18,401,850 | 773.23 |
| ID76 | 23,552,671 | 438.52 |
| ID77 | 15,590,138 | 651.77 |
| ID78 | 32,425,963 | 1,155.48 |
| ID79 | 16,787,870 | 763.10 |
| ID80 | 17,587,609 | 773.50 |
| ID81 | 15,202,857 | 710.37 |
| ID82 | 19,075,493 | 513.54 |
| ID83 | 15,780,914 | 660.98 |
| ID84 | 14,811,427 | 480.77 |
| ID85 | 18,786,904 | 788.84 |
| ID86 | 26,865,223 | 738.28 |
| ID87 | 18,720,499 | 726.24 |
| ID88 | 16,861,070 | 605.83 |
| ID89 | 19,833,421 | 765.86 |
| ID90 | 38,786,440 | 1,749.42 |
| ID91 | 16,290,439 | 803.91 |
| ID92 | 20,605,637 | 693.00 |
| ID93 | 17,319,321 | 685.55 |
| ID94 | 19,328,672 | 765.85 |
| ID95 | 18,733,298 | 768.73 |
| ID96 | 17,077,895 | 679.79 |
| ID97 | 13,378,492 | 591.48 |
| ID98 | 15,652,820 | 981.91 |
| ID99 | 18,500,737 | 897.83 |
| ID100 | 15,461,797 | 617.75 |
| ID101 | 14,159,039 | 794.76 |
| ID102 | 19,896,442 | 776.41 |
| ID103 | 15,618,283 | 670.77 |
| ID104 | 18,143,887 | 491.09 |
| ID105 | 16,646,566 | 862.83 |
| ID106 | 14,355,827 | 771.30 |
| ID107 | 16,154,856 | 465.40 |
| ID108 | 9,756,976 | 645.23 |
| ID109 | 12,601,930 | 399.70 |
| ID110 | 15,561,310 | 852.40 |
| ID111 | 13,634,162 | 747.92 |
| ID112 | 16,317,139 | 392.15 |
| ID113 | 18,365,796 | 892.56 |
| ID115 | 14,476,053 | 434.83 |
| ID116 | 16,990,447 | 995.74 |
| ID117 | 20,730,119 | 854.70 |
| ID119 | 13,032,551 | 544.59 |
| ID120 | 15,055,915 | 515.67 |
| ID121 | 96,578,775 | 1,435.53 |
| ID124 | 13,744,102 | 868.82 |
| ID125 | 16,999,874 | 872.98 |
| ID127 | 14,586,671 | 756.69 |
| ID129 | 24,639,500 | 248.91 |
| ID130 | 13,502,506 | 147.61 |
| ID131 | 30,822,195 | 448.10 |
| ID132 | 13,447,485 | 693.17 |
| ID133 | 23,665,872 | 415.50 |
| ID134 | 27,169,732 | 307.59 |
| ID135 | 16,955,197 | 429.59 |
| ID136 | 11,650,407 | 636.96 |
| ID137 | 22,413,730 | 696.80 |
| ID138 | 18,611,721 | 846.00 |
| ID141 | 16,299,470 | 726.81 |
| ID142 | 25,465,163 | 382.93 |
| ID143 | 33,363,163 | 249.72 |
| ID145 | 16,031,338 | 526.32 |
| ID146 | 15,686,777 | 592.76 |
| ID147 | 13,868,813 | 264.75 |
| ID148 | 11,135,309 | 371.86 |
| ID149 | 11,894,614 | 505.39 |
| ID150 | 19,567,129 | 884.97 |
| ID151 | 14,164,862 | 374.48 |
| ID152 | 14,899,784 | 427.48 |
| ID153 | 15,599,954 | 788.55 |
| ID156 | 16,449,675 | 337.71 |
| ID157 | 13,956,971 | 553.43 |
| ID158 | 13,685,945 | 409.67 |
| ID159 | 14,833,271 | 330.05 |
| ID160 | 13,403,314 | 468.05 |
| ID161 | 15,345,024 | 629.53 |
| ID162 | 15,327,442 | 614.84 |
| ID163 | 16,443,741 | 781.83 |
| ID164 | 22,250,184 | 875.21 |
| ID165 | 14,330,812 | 435.73 |
| ID166 | 15,816,282 | 377.55 |
| ID167 | 13,065,346 | 364.40 |
| ID168 | 19,688,348 | 726.76 |
| ID169 | 18,629,160 | 644.11 |
| ID170 | 13,960,145 | 472.83 |
| ID171 | 17,964,357 | 614.30 |
| ID173 | 14,552,179 | 566.68 |
| ID174 | 16,440,565 | 976.41 |
| ID175 | 15,983,941 | 463.94 |
| ID176 | 16,377,544 | 587.71 |
| ID177 | 18,787,227 | 867.49 |
| ID178 | 19,693,870 | 1,004.01 |
| ID183 | 18,998,418 | 1,047.62 |
| ID185 | 20,518,523 | 972.35 |
| ID186 | 41,349,601 | 320.55 |
| ID218 | 11,713,719 | 647.66 |
| ID275 | 16,263,485 | 936.51 |

Supplementary Table 1: Targeted sequencing statistics. The number of passing filter reads and average coverage are shown for each sample.

| Supplementary table 2: CHIP related variants characteristics | | | | | | | | | | | |
| --- | --- | --- | --- | --- | --- | --- | --- | --- | --- | --- | --- |
| Sample | Chr | Start | End | Gene | Variant type | DNA change | Protein change | dbSNP ID | VarSome | VAF | DEPTH |
| ID80 | chr17 | 7,674,212 | 7,674,212 | TP53 | nonsynonymous SNV | c.A751T | p.I251F | . | Likely Pathogenic | 0.025 | 929 |
| ID54 | chr17 | 7,674,229 | 7,674,229 | TP53 | nonsynonymous SNV | c.G734A | p.G245D | rs121912656 | Likely Pathogenic | 0.021 | 430 |
| ID113 | chr17 | 7,674,962 | 7,674,962 | TP53 | nonsynonymous SNV | c.C569T | p.P190L | . | Likely Pathogenic | 0.101 | 990 |
| ID151 | chr17 | 7,675,160 | 7,675,160 | TP53 | nonsynonymous SNV | c.C452G | p.P151R | . | Likely Pathogenic | 0.044 | 291 |
| ID18 | chr17 | 31,182,783 | 31,182,783 | NF1 | nonsynonymous SNV | c.G586T | p.D196Y | rs2952999 | Benign | 0.327 | 99 |
| ID44 | chr17 | 31,182,783 | 31,182,783 | NF1 | nonsynonymous SNV | c.G586T | p.D196Y | rs2952999 | Benign | 0.344 | 193 |
| ID130 | chr17 | 31,226,459 | 31,226,459 | NF1 | frameshift insertion | c.2027dupC | p.I679Dfs*20 | rs780157990 | Pathogenic | 0.331 | 80 |
| ID55 | chr17 | 31,229,457 | 31,229,457 | NF1 | nonsynonymous SNV | c.C2842A | p.Q948K | . | Uncertain Significance | 0.02 | 333 |
| ID158 | chr17 | 31,349,207 | 31,349,207 | NF1 | frameshift deletion | c.7277delG | p.C2426Lfs*5 | . | Pathogenic | 0.145 | 361 |
| ID111 | chr2 | 25,234,323 | 25,234,323 | DNMT3A | frameshift deletion | c.2695delC | p.R899Afs*6 | . | Pathogenic | 0.259 | 665 |
| ID125 | chr2 | 25,234,322 | 25,234,322 | DNMT3A | nonsynonymous SNV | c.G2696A | p.R899H | . | Likely Pathogenic | 0.065 | 894 |
| ID160 | chr2 | 25,234,373 | 25,234,373 | DNMT3A | nonsynonymous SNV | c.G2645A | p.R882H | rs147001633 | Pathogenic | 0.021 | 335 |
| ID61 | chr2 | 25,234,373 | 25,234,373 | DNMT3A | nonsynonymous SNV | c.G2645A | p.R882H | rs147001633 | Pathogenic | 0.188 | 488 |
| ID19 | chr2 | 25,234,374 | 25,234,374 | DNMT3A | nonsynonymous SNV | c.C2644T | p.R882C | rs377577594 | Pathogenic | 0.201 | 685 |
| ID56 | chr2 | 25,234,374 | 25,234,374 | DNMT3A | nonsynonymous SNV | c.C2644T | p.R882C | rs377577594 | Pathogenic | 0.049 | 734 |
| ID76 | chr2 | 25,234,374 | 25,234,374 | DNMT3A | nonsynonymous SNV | c.C2644T | p.R882C | rs377577594 | Pathogenic | 0.2 | 431 |
| ID20 | chr2 | 25,234,386 | 25,234,386 | DNMT3A | nonsynonymous SNV | c.T2632C | p.S878P | . | Likely Pathogenic | 0.02 | 711 |
| ID109 | chr2 | 25,235,795 | 25,235,795 | DNMT3A | frameshift deletion | c.2509delT | p.S837Qfs*3 | . | Pathogenic | 0.02 | 432 |
| ID72 | chr2 | 25,236,937 | 25,236,937 | DNMT3A | nonsynonymous SNV | c.A2477G | p.K826R | rs770079872 | Pathogenic | 0.038 | 507 |
| ID98 | chr2 | 25,236,937 | 25,236,937 | DNMT3A | nonsynonymous SNV | c.A2477C | p.K826T | . | Pathogenic | 0.091 | 666 |
| ID7 | chr2 | 25,239,199 | 25,239,199 | DNMT3A | nonsynonymous SNV | c.T2339C | p.I780T | rs370751539 | Likely Pathogenic | 0.232 | 505 |
| ID98 | chr2 | 25,240,306 | 25,240,306 | DNMT3A | nonsynonymous SNV | c.T2318G | p.L773R | rs764303486 | Likely Pathogenic | 0.042 | 1,026 |
| ID76 | chr2 | 25,240,397 | 25,240,397 | DNMT3A | nonsynonymous SNV | c.C2227T | p.P743S | . | Likely Pathogenic | 0.025 | 452 |
| ID56 | chr2 | 25,240,418 | 25,240,418 | DNMT3A | nonsynonymous SNV | c.C2206T | p.R736C | rs761934754 | Likely Pathogenic | 0.038 | 889 |
| ID148 | chr2 | 25,240,420 | 25,240,420 | DNMT3A | nonsynonymous SNV | c.A2204G | p.Y735C | rs147828672 | Likely Pathogenic | 0.06 | 262 |
| ID18 | chr2 | 25,240,431 | 25,240,431 | DNMT3A | nonsynonymous SNV | c.C2193G | p.F731L | . | Likely Pathogenic | 0.261 | 405 |
| ID82 | chr2 | 25,241,687 | 25,241,687 | DNMT3A | nonsynonymous SNV | c.T1957G | p.L653V | . | Likely Pathogenic | 0.346 | 848 |
| ID161 | chr2 | 25,243,933 | 25,243,933 | DNMT3A | nonsynonymous SNV | c.T1901C | p.I634T | . | Likely Pathogenic | 0.111 | 443 |
| ID105 | chr2 | 25,243,965 | 25,243,965 | DNMT3A | stopgain | c.C1869G | p.Y623X | . | Pathogenic | 0.127 | 853 |
| ID42 | chr2 | 25,244,338 | 25,244,338 | DNMT3A | nonsynonymous SNV | c.G1668T | p.R556S | . | Pathogenic | 0.046 | 428 |
| ID111 | chr2 | 25,244,580 | 25,244,580 | DNMT3A | nonsynonymous SNV | c.G1627T | p.G543C | rs752222356 | Likely Pathogenic | 0.051 | 690 |
| ID178 | chr2 | 25,244,612 | 25,244,612 | DNMT3A | nonsynonymous SNV | c.G1595A | p.G532D | . | Likely Pathogenic | 0.192 | 881 |
| ID178 | chr2 | 25,245,290 | 25,245,290 | DNMT3A | nonsynonymous SNV | c.A1517G | p.H506R | . | Likely Pathogenic | 0.18 | 987 |
| ID45 | chr2 | 25,246,176 | 25,246,176 | DNMT3A | frameshift deletion | c.1413delT | p.I471Mfs*179 | . | Pathogenic | 0.096 | 581 |
| ID186 | chr2 | 25,246,270 | 25,246,270 | DNMT3A | stopgain | c.G1319A | p.W440X | rs773260349 | Pathogenic | 0.02 | 330 |
| ID147 | chr2 | 25,247,110 | 25,247,110 | DNMT3A | frameshift deletion | c.1063delC | p.H355Tfs*51 | . | Pathogenic | 0.047 | 212 |
| ID72 | chr2 | 25,247,629 | 25,247,629 | DNMT3A | nonsynonymous SNV | c.C976T | p.R326C | rs747448117 | Uncertain Significance | 0.161 | 547 |
| ID15 | chr2 | 25,247,715 | 25,247,715 | DNMT3A | stopgain | c.G890A | p.W297X | . | Likely Pathogenic | 0.042 | 397 |
| ID133 | chr20 | 32,433,542 | 32,433,558 | ASXL1 | frameshift deletion | c.1329_1345del | p.Y444fs*0 | . | Uncertain Significance | 0.048 | 650 |
| ID116 | chr20 | 32,434,435 | 32,434,435 | ASXL1 | stopgain | c.C1708T | p.Q570X | rs747847938 | Pathogenic | 0.027 | 807 |
| ID10 | chr20 | 32,434,600 | 32,434,622 | ASXL1 | frameshift deletion | c.1873_1895del | p.E630Rfs*13 | rs766433101 | Pathogenic | 0.044 | 671 |
| ID169 | chr20 | 32,434,600 | 32,434,622 | ASXL1 | frameshift deletion | c.1873_1895del | p.E630Rfs*13 | rs766433101 | Pathogenic | 0.032 | 616 |
| ID96 | chr20 | 32,435,579 | 32,435,579 | ASXL1 | frameshift deletion | c.2852delT | p.T952Lfs*26 | . | Likely Pathogenic | 0.08 | 1,160 |
| ID109 | chr4 | 105,234,654 | 105,234,654 | TET2 | frameshift insertion | c.713dupT | p.S239Ffs*14 | . | Pathogenic | 0.046 | 556 |
| ID110 | chr4 | 105,234,654 | 105,234,654 | TET2 | frameshift insertion | c.713dupT | p.S239Ffs*14 | . | Pathogenic | 0.045 | 1,106 |
| ID18 | chr4 | 105,235,163 | 105,235,163 | TET2 | frameshift insertion | c.1222dupC | p.P410Sfs*32 | . | Likely Pathogenic | 0.051 | 351 |
| ID44 | chr4 | 105,235,626 | 105,235,626 | TET2 | frameshift deletion | c.1684delC | p.P562Qfs*5 | . | Pathogenic | 0.02 | 664 |
| ID77 | chr4 | 105,235,713 | 105,235,713 | TET2 | stopgain | c.C1771T | p.Q591X | . | Pathogenic | 0.219 | 746 |
| ID166 | chr4 | 105,236,095 | 105,236,095 | TET2 | frameshift deletion | c.2153delT | p.L719Cfs*31 | . | Pathogenic | 0.149 | 517 |
| ID108 | chr4 | 105,236,166 | 105,236,166 | TET2 | stopgain | c.C2224T | p.Q742X | . | Pathogenic | 0.024 | 461 |
| ID18 | chr4 | 105,236,464 | 105,236,464 | TET2 | frameshift deletion | c.2522delT | p.S842Qfs*30 | . | Likely Pathogenic | 0.043 | 308 |
| ID18 | chr4 | 105,236,568 | 105,236,568 | TET2 | stopgain | c.C2626T | p.Q876X | rs781668759 | Pathogenic | 0.045 | 694 |
| ID164 | chr4 | 105,237,290 | 105,237,290 | TET2 | frameshift deletion | c.3348delA | p.N1118Ifs*18 | . | Pathogenic | 0.058 | 744 |
| ID11 | chr4 | 105,242,912 | 105,242,912 | TET2 | stopgain | c.T3579A | p.C1193X | . | Pathogenic | 0.1 | 288 |
| ID22 | chr4 | 105,242,914 | 105,242,914 | TET2 | nonsynonymous SNV | c.C3581G | p.P1194R | . | Likely Pathogenic | 0.145 | 415 |
| ID61 | chr4 | 105,243,714 | 105,243,714 | TET2 | stopgain | c.G3739T | p.E1247X | . | Pathogenic | 0.026 | 506 |
| ID98 | chr4 | 105,259,626 | 105,259,626 | TET2 | frameshift insertion | c.3812dupG | p.C1271Wfs*28 | rs755283040 | Pathogenic | 0.107 | 949 |
| ID95 | chr4 | 105,259,665 | 105,259,665 | TET2 | nonsynonymous SNV | c.T3850G | p.S1284A | . | Uncertain Significance | 0.058 | 961 |
| ID95 | chr4 | 105,259,669 | 105,259,669 | TET2 | nonsynonymous SNV | c.T3854C | p.F1285S | . | Uncertain Significance | 0.056 | 956 |
| ID66 | chr4 | 105,259,723 | 105,259,723 | TET2 | nonsynonymous SNV | c.G3908T | p.S1303I | . | Likely Pathogenic | 0.032 | 922 |
| ID85 | chr4 | 105,261,774 | 105,261,775 | TET2 | frameshift deletion | c.3970_3971del | p.H1325Ffs*11 | . | Pathogenic | 0.028 | 412 |
| ID113 | chr4 | 105,272,571 | 105,272,571 | TET2 | nonsynonymous SNV | c.C4190T | p.T1397I | . | Likely Pathogenic | 0.02 | 689 |
| ID166 | chr4 | 105,272,597 | 105,272,597 | TET2 | frameshift deletion | c.4216delT | p.F1406Lfs*41 | . | Pathogenic | 0.049 | 435 |
| ID81 | chr4 | 105,272,811 | 105,272,811 | TET2 | frameshift deletion | c.4430delA | p.K1478Sfs*92 | . | Pathogenic | 0.098 | 488 |
| ID94 | chr4 | 105,275,347 | 105,275,347 | TET2 | nonsynonymous SNV | c.A4837G | p.N1613D | . | Likely Pathogenic | 0.025 | 1,047 |
| ID166 | chr4 | 105,276,064 | 105,276,064 | TET2 | stopgain | c.C5554T | p.Q1852X | rs549395077 | Uncertain Significance | 0.083 | 627 |
| ID21 | chr4 | 105,276,203 | 105,276,203 | TET2 | nonsynonymous SNV | c.C5693T | p.S1898F | rs767475870 | Likely Pathogenic | 0.028 | 852 |
| ID11 | chr9 | 5,069,037 | 5,069,037 | JAK2 | stopgain | c.G1342T | p.E448X | . | Pathogenic | 0.022 | 221 |
| ID107 | chr9 | 5,073,770 | 5,073,770 | JAK2 | nonsynonymous SNV | c.G1849T | p.V617F | rs77375493 | Pathogenic | 0.042 | 327 |
| ID165 | chr9 | 5,073,770 | 5,073,770 | JAK2 | nonsynonymous SNV | c.G1849T | p.V617F | rs77375493 | Pathogenic | 0.029 | 382 |
| ID166 | chr9 | 5,073,770 | 5,073,770 | JAK2 | nonsynonymous SNV | c.G1849T | p.V617F | rs77375493 | Pathogenic | 0.021 | 418 |
| ID98 | chr9 | 5,073,770 | 5,073,770 | JAK2 | nonsynonymous SNV | c.G1849T | p.V617F | rs77375493 | Pathogenic | 0.146 | 591 |
| ID82 | chr9 | 136,500,721 | 136,500,721 | NOTCH1 | nonsynonymous SNV | c.A5765G | p.N1922S | rs765708087 | Benign | 0.025 | 885 |
| ID11 | chrX | 45,062,653 | 45,062,653 | KDM6A | stopgain | c.G1432T | p.E478X | . | Uncertain Significance | 0.021 | 331 |
| ID22 | chrX | 45,090,729 | 45,090,729 | KDM6A | nonsynonymous SNV | c.A3743G | p.Q1248R | . | Uncertain Significance | 0.028 | 446 |

Supplementary Table 2: CHIP-related variants characteristics. For each variant, from left to right, the sample name, the genomic coordinate (chromosome, start, end, and gene name), the variant type, the substitution effect (on DNA and protein), the dbSNP ID (when available), the VarSome clinical classification, the Variant Allele Frequency, and the coverage are reported.

| **Supplementary Table 3: Baseline characteristics, echocardiographic and laboratory parameters in SAVR patients with and without CHIP** | | | | | | | |
| --- | --- | --- | --- | --- | --- | --- | --- |
|  | **TOTAL MEAN** | **TOTAL SD** | **No-CHIP MEAN (83)** | **No-CHIP SD** | **CHIP MEAN (29)** | **CHIP SD** | **P-value** |
| Age (112) | 77.29 | 4.43 | 76.70 | 4.57 | 78.96 | 3.60 | **0.020** |
| Weight kg (112) | 72.94 | 11.35 | 73.00 | 11.53 | 72.76 | 11.01 | 0.960 |
| Height cm (112) | 164.30 | 8.41 | 164.36 | 8.20 | 164.10 | 9.13 | 0.837 |
| BMI kg m2 (112) | 26.99 | 3.52 | 26.96 | 3.42 | 27.05 | 3.85 | 0.900 |
| SEX (F-M) (112) | 58 (51.7%)-54 |  | 42 (50.6%)-41 |  | 16 (55.1%)-13 |  | 0.837 |
| Hypertension (YES-NO) (112) | 94 (83.9%)-18 |  | 71 (85.5%)-12 |  | 23 (79.3%)-6 |  | 0.548 |
| Dyslipidemia (YES-NO) (112) | 76 (67.8%)-36 |  | 55 (66.2%)-28 |  | 21 (72.4%)-8 |  | 0.654 |
| Type 2 Diabetes mellitus (YES-NO) (110) | 28 (25.4%)-82 |  | 22 (26.8%)-60 |  | 6 (20.6%)-22 |  | 0.640 |
| Atrial fibrillation (YES-NO) (112) | 22 (19.6%)-90 |  | 16 (19.2%)-67 |  | 6 (20.6%)-23 |  | 1 |
| Peripheral artery disease (YES-NO) (111) | 12 (10.8%)-99 |  | 9 (10.8%)-73 |  | 3 (10.3%)-26 |  | 1 |
| COPD (YES-NO) (112) | 12 (10.7%)-100 |  | 8 (9.6%)-75 |  | 4 (13.7%)-25 |  | 0.721 |
| Prior MI (YES-NO) (111) | 12 (10.8%)-99 |  | 10 (12%)-73 |  | 2 (7.1%)-26 |  | 0.536 |
| Smoking history (YES-NO) (112) | 36 (32%)-76 |  | 26 (31.3%)-57 |  | 10 (34.5%)-19 |  | 0.814 |
| Coronary Artery Disease (YES-NO) (112) | 48 (42.8%)-64 |  | 34 (40.9%)-49 |  | 14 (48.2%)-15 |  | 0.539 |
| ANGINA CLASS (>3 YES-NO) (109) | 4 (3.6%)-105 |  | 3 (3.7%)-77 |  | 1 (3.4%)-28 |  | 0.304 |
| NYHA III+IV (YES-NO) (109) | 24 (22%)-85 |  | 16 (19.7%)-65 |  | 8 (28.5%)-20 |  | 0.435 |
|  |  |  |  |  |  |  |  |
| **Echocardiographic characteristics** |  |  |  |  |  |  |  |
| LV EF (112) | 60.24 | 10.84 | 60.36 | 11.28 | 59.91 | 9.67 | 0.439 |
| LV mass g (104) | 130.43 | 112.34 | 119.36 | 110.23 | 163.63 | 114.22 | 0.085 |
| Mean aortic gradient mmHg (112) | 45.84 | 13.25 | 45.45 | 12.24 | 46.97 | 15.99 | 0.793 |
| Functional AVA cm2 (103) | 0.75 | 0.21 | 0.75 | 0.22 | 0.73 | 0.17 | 0.813 |
| Mitral regurgitation (>3+ YES-NO) (111) | 12 (10.8%)-99 |  | 7 (8.5%)-75 |  | 5 (17.2%)-24 |  | 0.284 |
| Tricuspid regurgitation (>3+ YES-NO) (101) | 5 (4.9%)-96 |  | 3 (4%)-72 |  | 2 (7.7%)-24 |  | 0.605 |
| Aortic regurgitation (>3+ YES-NO) (112) | 13 (11.6%)-99 |  | 10 (12%)-73 |  | 3 (10.3%)-26 |  | 1 |
|  |  |  |  |  |  |  |  |
| **Laboratory** |  |  |  |  |  |  |  |
| White blood count (112) | 7.58 | 2.14 | 7.32 | 1.98 | 8.32 | 2.43 | **0.030** |
| Neutrophils (112) | 5.16 | 2.04 | 4.94 | 1.88 | 5.80 | 2.35 | **0.035** |
| Lymphocytes (112) | 1.61 | 0.57 | 1.59 | 0.53 | 1.67 | 0.68 | 0.785 |
| Monocytes (112) | 0.61 | 0.23 | 0.60 | 0.21 | 0.63 | 0.30 | 0.997 |
| Eosinophils (112) | 0.16 | 0.14 | 0.15 | 0.14 | 0.18 | 0.15 | 0.496 |
| Basophils (112) | 0.03 | 0.05 | 0.02 | 0.04 | 0.04 | 0.06 | 0.048 |
| Hemoglobin g dl (112) | 13.07 | 1.58 | 13.14 | 1.48 | 12.88 | 1.83 | 0.192 |
| Platelets (112) | 216.36 | 70.12 | 214.82 | 67.40 | 220.76 | 78.49 | 0.664 |
| Creatinine mg dl (112) | 0.98 | 0.32 | 0.96 | 0.29 | 1.04 | 0.38 | 0.469 |
| Crockoft-gault eGFR ml min (>60 YES-NO) (112) | 66 (58.9%)-46 |  | 51 (61.4%)-32 |  | 15 (51.7%)-14 |  | 0.486 |
| Total cholesterol mg dl (110) | 163.73 | 33.69 | 165.92 | 34.85 | 157.00 | 29.44 | 0.331 |
| Triglycerides mg dl (110) | 113.29 | 55.38 | 116.92 | 60.11 | 102.15 | 35.94 | 0.399 |
| HDL mg dl (108) | 50.81 | 15.19 | 51.27 | 15.88 | 49.44 | 13.10 | 0.683 |
| Albumin g dl (103) | 4.15 | 0.31 | 4.16 | 0.31 | 4.12 | 0.30 | 0.474 |
| LDL (107) | 90.92 | 27.46 | 92.19 | 28.93 | 87.13 | 22.63 | 0.725 |

Supplementary Table 3: Baseline characteristics, echocardiographic and laboratory parameters in Surgical AVR (SAVR) patients with and without CHIP. Each column displays the mean or the standard deviation (SD) for the continuous variables of both groups (Total), the No-CHIP or the CHIP samples. For dichotomous variables, the total number of each variable and the percentage of the first one are reported. In the last column, the resulting p-value for each No-CHIP vs CHIP test is shown and the significant ones are written in bold.

| **Supplementary Table 4: Baseline characteristics, echocardiographic and laboratory parameters in male patients with and without CHIP** | | | | | | | |
| --- | --- | --- | --- | --- | --- | --- | --- |
|  | **TOTAL MEAN** | **TOTAL SD** | **No-CHIP MEAN (60)** | **No-CHIP SD** | **CHIP MEAN (21)** | **CHIP SD** | **P-value** |
| Age (81) | 79.04 | 4.81 | 78.95 | 5.06 | 79.29 | 4.10 | 0.829 |
| Weight kg (81) | 77.01 | 11.13 | 77.77 | 10.81 | 74.86 | 12.04 | 0.313 |
| Height cm (81) | 169.75 | 5.96 | 169.60 | 5.37 | 170.19 | 7.52 | 0.729 |
| BMI kg m2 (81) | 26.73 | 3.71 | 27.05 | 3.72 | 25.82 | 3.61 | 0.155 |
| AVR Type (SAVR-TAVR) (81) | 54 (66.6%)-27 |  | 41 (68.3%)-19 |  | 13 (61.9%)-8 |  | 0.780 |
| Hypertension (YES-NO) (81) | 68 (83.9%)-13 |  | 49 (81.6%)-11 |  | 19 (90.4%)-2 |  | 0.512 |
| Dyslipidemia (YES-NO) (81) | 62 (76.5%)-19 |  | 42 (70%)-18 |  | 20 (95.2%)-1 |  | **0.033** |
| Type 2 Diabetes mellitus (YES-NO) (80) | 23 (28.7%)-57 |  | 18 (30%)-42 |  | 5 (25%)-15 |  | 0.784 |
| Atrial fibrillation (YES-NO) (81) | 24 (29.6%)-57 |  | 18 (30%)-42 |  | 6 (28.5%)-15 |  | 1 |
| Peripheral artery disease (YES-NO) (81) | 22 (27.1%)-59 |  | 13 (21.6%)-47 |  | 9 (42.8%)-12 |  | 0.093 |
| COPD (YES-NO) (81) | 13 (16%)-68 |  | 8 (13.3%)-52 |  | 5 (23.8%)-16 |  | 0.305 |
| Prior MI (YES-NO) (81) | 12 (14.8%)-69 |  | 8 (13.3%)-52 |  | 4 (19%)-17 |  | 0.740 |
| Smoking history (YES-NO) (81) | 33 (40.7%)-48 |  | 23 (38.3%)-37 |  | 10 (47.6%)-11 |  | 0.596 |
| Coronary Artery Disease (YES-NO) (81) | 43 (53%)-38 |  | 30 (50%)-30 |  | 13 (62%)-8 |  | 0.449 |
| ANGINA CLASS (>3 YES-NO) (71) | 5 (7%)-66 |  | 2 (3.8%)-50 |  | 3 (15.7%)-16 |  | 0.123 |
| NYHA III+IV (YES-NO) (71) | 58 (81.6%)-13 |  | 42 (79.2%)-11 |  | 16 (88.8%)-2 |  | 0.479 |
|  |  |  |  |  |  |  |  |
| **Echocardiographic characteristics** |  |  |  |  |  |  |  |
| LV EF (81) | 57.26 | 10.57 | 58.09 | 10.09 | 54.91 | 11.78 | 0.303 |
| LV mass g (52) | 137.66 | 118.63 | 128.99 | 120.49 | 161.20 | 114.35 | 0.650 |
| Mean aortic gradient mmHg (80) | 43.40 | 9.97 | 43.70 | 10.73 | 42.50 | 7.42 | 0.395 |
| Functional AVA cm2 (60) | 0.82 | 0.21 | 0.82 | 0.22 | 0.81 | 0.16 | 0.978 |
| Mitral regurgitation (>3+ YES-NO) (78) | 6 (7.7%)-72 |  | 3 (5.1%)-55 |  | 3 (15%)-17 |  | 0.325 |
| Tricuspid regurgitation (>3+ YES-NO) (61) | 1 (1.6%)-60 |  | 1 (2.2%)-43 |  | -17 (100%) |  | NaN |
| Aortic regurgitation (>3+ YES-NO) (80) | 10 (12.5%)-70 |  | 7 (11.8%)-52 |  | 3 (14.3%)-18 |  | 1 |
|  |  |  |  |  |  |  |  |
| **Laboratory** |  |  |  |  |  |  |  |
| White blood count (80) | 7.88 | 2.35 | 7.71 | 2.16 | 8.34 | 2.83 | 0.364 |
| Neutrophils (80) | 5.47 | 2.28 | 5.30 | 2.10 | 5.94 | 2.72 | 0.291 |
| Lymphocytes (80) | 1.51 | 0.56 | 1.52 | 0.54 | 1.48 | 0.62 | 0.606 |
| Monocytes (80) | 0.66 | 0.24 | 0.66 | 0.24 | 0.68 | 0.24 | 0.699 |
| Eosinophils (80) | 0.20 | 0.19 | 0.2 | 0.20 | 0.20 | 0.16 | 0.838 |
| Basophils (80) | 0.03 | 0.04 | 0.02 | 0.04 | 0.04 | 0.05 | 0.155 |
| Hemoglobin g dl (81) | 13.25 | 1.56 | 13.43 | 1.60 | 12.73 | 1.38 | 0.089 |
| Platelets (81) | 205.83 | 61.53 | 209.87 | 63.21 | 194.29 | 56.32 | 0.316 |
| Creatinine mg dl (81) | 1.20 | 0.44 | 1.19 | 0.49 | 1.215 | 0.28 | 0.183 |
| Crockoft-gault eGFR ml min (>60 YES-NO) (81) | 28 (34.5%)-53 |  | 24 (40%)-36 |  | 4 (19%)-17 |  | 0.141 |
| Total cholesterol mg dl (80) | 156.51 | 30.48 | 156.35 | 31.06 | 157.00 | 29.45 | 0.942 |
| Triglycerides mg dl (80) | 111.60 | 56.32 | 112.45 | 59.90 | 109.05 | 45.17 | 0.987 |
| HDL mg dl (79) | 49.37 | 13.72 | 49.78 | 13.91 | 48.15 | 13.44 | 0.460 |
| Albumin g dl (7) | 4.03 | 0.39 | 4.04 | 0.41 | 3.98 | 0.34 | 0.512 |
| LDL (78) | 86.02 | 24.91 | 85.66 | 25.82 | 87.04 | 22.65 | 0.697 |

Supplementary Table 4: Baseline characteristics, echocardiographic and laboratory parameters in male patients with and without CHIP. Each column displays the mean or the standard deviation (SD) for the continuous variables of both groups (Total), the No-CHIP or the CHIP samples. For dichotomous variables, the total number of each variable and the percentage of the first one are reported. In the last column, the resulting p-value for each No-CHIP vs CHIP test is shown. The significant p-values are written in bold, instead, “NaN” indicates when it was not possible to calculate it.

| **Supplementary Table 5: Baseline characteristics, echocardiographic and laboratory parameters in female patients with and without CHIP** | | | | | | | |
| --- | --- | --- | --- | --- | --- | --- | --- |
|  | **TOTAL MEAN** | **TOTAL SD** | **No-CHIP MEAN (57)** | **No-CHIP SD** | **CHIP MEAN (30)** | **CHIP SD** | **P-value** |
| Age (87) | 79.31 | 5.59 | 77.91 | 5.60 | 81.97 | 4.57 | **0.001** |
| Weight kg (87) | 68.29 | 11.32 | 67.79 | 11.44 | 69.23 | 11.21 | 0.613 |
| Height cm (87) | 158.61 | 6.77 | 158.74 | 6.87 | 158.37 | 6.68 | 0.628 |
| BMI kg m2 (87) | 27.10 | 4.02 | 26.86 | 4.06 | 27.56 | 3.98 | 0.532 |
| AVR Type (SAVR-TAVR) (87) | 58 (66.6%)-29 |  | 42 (73.6%)-15 |  | 16 (53.3%)-14 |  | 0.088 |
| Hypertension (YES-NO) (87) | 71 (81.6%)-16 |  | 48 (84.2%)-9 |  | 23 (76.6%)-7 |  | 0.553 |
| Dyslipidemia (YES-NO) (87) | 55 (63.2%)-32 |  | 35 (61.4%)-22 |  | 20 (66.6%)-10 |  | 0.644 |
| Type 2 Diabetes mellitus (YES-NO) (85) | 20 (23.5%)-65 |  | 13 (23.6%)-42 |  | 7 (23.3%)-23 |  | 1 |
| Atrial fibrillation (YES-NO) (87) | 18 (20.6%)-69 |  | 12 (21%)-45 |  | 6 (20%)-24 |  | 1 |
| Peripheral artery disease (YES-NO) (86) | 12 (13.9%)-74 |  | 7 (12.5%)-49 |  | 5 (16.6%)-25 |  | 0.738 |
| COPD (YES-NO) (87) | 9 (10.3%)-78 |  | 5 (8.7%)-52 |  | 4 (13.3%)-26 |  | 0.704 |
| Prior MI (YES-NO) (86) | 7 (8.1%)-79 |  | 6 (10.5%)-51 |  | 1 (3.4%)-28 |  | 0.410 |
| Smoking history (YES-NO) (87) | 13 (14.9%)-74 |  | 10 (17.5%)-47 |  | 3 (10%)-27 |  | 0.524 |
| Coronary Artery Disease (YES-NO) (87) | 29 (33.3%)-58 |  | 17 (29.8%)-40 |  | 12 (40%)-18 |  | 0.480 |
| ANGINA CLASS (>3 YES-NO) (81) | 6 (7.4%)-75 |  | 4 (7.4%)-50 |  | 2 (7.4%)-25 |  | 1 |
| NYHA III+IV (YES-NO) (80) | 28 (35%)-52 |  | 16 (30.1%)-37 |  | 12 (44.4%)-15 |  | 0.232 |
|  |  |  |  |  |  |  |  |
| **Echocardiographic characteristics** |  |  |  |  |  |  |  |
| LV EF (86) | 60.93 | 10.86 | 60.82 | 11.63 | 61.13 | 9.44 | 0.502 |
| LV mass g (54) | 125.90 | 105.35 | 110.22 | 100.19 | 170.69 | 110.54 | **0.043** |
| Mean aortic gradient mmHg (87) | 48.28 | 14.42 | 47.91 | 13.13 | 48.97 | 16.81 | 0.802 |
| Functional AVA cm2 (87) | 0.68 | 0.19 | 0.68 | 0.20 | 0.67 | 0.16 | 0.968 |
| Mitral regurgitation (>3+ YES-NO) (86) | 10 (11.6%)-76 |  | 5 (8.9%)-51 |  | 5 (16.6%)-25 |  | 0.303 |
| Tricuspid regurgitation (>3+ YES-NO) (62) | 4 (6.4%)-58 |  | 2 (4.8%)-39 |  | 2 (9.5%)-19 |  | 0.593 |
| Aortic regurgitation (>3+ YES-NO) (86) | 5 (5.8%)-81 |  | 3 (5.3%)-53 |  | 2 (6.6%)-28 |  | 1 |
|  |  |  |  |  |  |  |  |
| **Laboratory** |  |  |  |  |  |  |  |
| White blood count (87) | 7.45 | 2.25 | 7.37 | 2.42 | 7.61 | 1.90 | 0.228 |
| Neutrophils (87) | 5.87 | 7.72 | 5.07 | 2.37 | 7.40 | 12.73 | 0.311 |
| Lymphocytes (87) | 1.78 | 1.65 | 1.58 | 0.59 | 2.17 | 2.68 | 0.463 |
| Monocytes (87) | 0.66 | 0.87 | 0.56 | 0.18 | 0.85 | 1.46 | 0.946 |
| Eosinophils (87) | 0.14 | 0.17 | 0.13 | 0.15 | 0.17 | 0.21 | 0.303 |
| Basophils (87) | 0.03 | 0.06 | 0.03 | 0.04 | 0.05 | 0.09 | 0.248 |
| Hemoglobin g dl (87) | 12.46 | 1.50 | 12.56 | 1.21 | 12.27 | 1.94 | 0.071 |
| Platelets (87) | 226.33 | 79.39 | 232.39 | 75.35 | 214.83 | 86.70 | 0.268 |
| Creatinine mg dl (87) | 0.95 | 0.34 | 0.90 | 0.29 | 1.06 | 0.41 | 0.100 |
| Crockoft-gault eGFR ml min (>60 YES-NO) (87) | 58 (66.6%)-29 |  | 42 (73.6%)-15 |  | 16 (53.3%)-14 |  | 0.094 |
| Total cholesterol mg dl (85) | 169.42 | 36.27 | 176.74 | 36.38 | 154.54 | 31.68 | **0.004** |
| Triglycerides mg dl (85) | 114.72 | 48.81 | 121.54 | 53.33 | 100.82 | 34.86 | 0.145 |
| HDL mg dl (84) | 52.63 | 15.20 | 54.66 | 16.88 | 48.57 | 10.21 | 0.140 |
| Albumin g dl (82) | 4.03 | 0.34 | 4.07 | 0.33 | 3.95 | 0.36 | 0.131 |
| LDL (84) | 93.50 | 29.73 | 97.36 | 31.14 | 85.80 | 25.50 | 0.122 |

Supplementary Table 5: Baseline characteristics, echocardiographic and laboratory parameters in female patients with and without CHIP. Each column displays the mean or the standard deviation (SD) for the continuous variables of both groups (Total), the No-CHIP or the CHIP samples. For dichotomous variables, the total number of each variable and the percentage of the first one are reported. In the last column, the resulting p-value for each No-CHIP vs CHIP test is shown and the significant ones are written in bold.

| Supplementary Table 6: RNA-Seq sequencing statistics | | | |
| --- | --- | --- | --- |
| Sample | Group | Mapped reads | Percentage of aligned reads |
| ID45 | CHIP | 22,263,482 | 85.65% |
| ID72 | CHIP | 4,588,989 | 73.61% |
| ID76 | CHIP | 9,612,871 | 78.23% |
| ID111 | CHIP | 13,853,335 | 80.12% |
| ID125 | CHIP | 9,749,336 | 82.60% |
| ID161 | CHIP | 6,485,491 | 79.55% |
| ID186 | CHIP | 8,934,923 | 82.52% |
| ID66 | CHIP | 6,572,496 | 67.85% |
| ID85 | CHIP | 11,921,087 | 78.98% |
| ID109 | CHIP | 8,357,765 | 82.28% |
| ID110 | CHIP | 12,731,921 | 84.57% |
| ID64 | No-CHIP | 9,704,635 | 79.41% |
| ID65 | No-CHIP | 8,105,644 | 80.96% |
| ID87 | No-CHIP | 11,159,153 | 83.91% |
| ID91 | No-CHIP | 9,247,704 | 74.47% |
| ID145 | No-CHIP | 20,625,906 | 83.86% |
| ID173 | No-CHIP | 6,849,705 | 79.44% |
| ID218 | No-CHIP | 9,828,418 | 86.24% |
| ID275 | No-CHIP | 11,616,654 | 84.52% |
| ID179 | Control | 7,447,958 | 87.39% |
| ID190 | Control | 6,894,232 | 78.60% |
| ID205 | Control | 18,096,943 | 85.88% |
| ID259 | Control | 8,220,733 | 84.32% |
| ID278 | Control | 11,419,798 | 85.84% |

Supplementary Table 6: RNA-Seq sequencing statistics. The number of mapped reads and the percentage of aligned reads are shown for each sample in each group.

| **Supplementary Table 7: Differentially Expressed Genes in No-CHIP vs Control contrast** | | | | | | | |
| --- | --- | --- | --- | --- | --- | --- | --- |
| **Genes** | **baseMean** | **log2FoldChange** | **lfcSE** | **stat** | **pvalue** | **padj** | **FoldChange** |
| ISG15 | 172.46 | 0.97 | 0.26 | 3.70 | 0.00021 | 0.01002 | 1.96 |
| AGRN | 228.09 | 0.60 | 0.20 | 3.10 | 0.00192 | 0.03996 | 1.52 |
| FNDC10 | 13.67 | 1.48 | 0.40 | 3.75 | 0.00018 | 0.00914 | 2.79 |
| KCNAB2 | 138.62 | 1.02 | 0.25 | 4.12 | 3.75E-05 | 0.00321 | 2.03 |
| SLC2A5 | 31.71 | 2.67 | 0.49 | 5.42 | 5.85E-08 | 3.13E-05 | 6.36 |
| PDPN | 83.54 | 1.85 | 0.40 | 4.66 | 3.18E-06 | 0.00058 | 3.62 |
| TMEM51 | 31.71 | 0.86 | 0.28 | 3.08 | 0.00205 | 0.04114 | 1.81 |
| PLA2G2D | 11.20 | 5.09 | 1.39 | 3.66 | 0.00025 | 0.01123 | 34.16 |
| C1QA | 1,673.90 | 0.87 | 0.24 | 3.62 | 0.00029 | 0.01191 | 1.83 |
| C1QC | 1,087.01 | 0.94 | 0.28 | 3.37 | 0.00076 | 0.02278 | 1.92 |
| C1QB | 1,534.44 | 0.85 | 0.25 | 3.37 | 0.00074 | 0.02227 | 1.81 |
| MYOM3 | 65.13 | 0.85 | 0.26 | 3.31 | 0.00092 | 0.02562 | 1.81 |
| RUNX3 | 16.45 | 1.64 | 0.54 | 3.01 | 0.0026 | 0.04841 | 3.11 |
| CD52 | 42.60 | 1.67 | 0.52 | 3.25 | 0.00115 | 0.0294 | 3.19 |
| RPS6KA1 | 100.06 | 0.83 | 0.28 | 3.00 | 0.00272 | 0.04949 | 1.78 |
| IFI6 | 385.85 | 1.12 | 0.26 | 4.24 | 2.22E-05 | 0.00224 | 2.18 |
| PTAFR | 112.13 | 1.06 | 0.25 | 4.24 | 2.27E-05 | 0.00225 | 2.08 |
| LAPTM5 | 883.56 | 1.37 | 0.32 | 4.23 | 2.30E-05 | 0.00227 | 2.58 |
| LCK | 30.72 | 1.79 | 0.52 | 3.43 | 0.00061 | 0.01963 | 3.46 |
| COL8A2 | 594.11 | 0.68 | 0.19 | 3.56 | 0.00037 | 0.01387 | 1.61 |
| BMP8A | 27.62 | 1.85 | 0.60 | 3.08 | 0.00204 | 0.04105 | 3.61 |
| HIVEP3 | 71.40 | 0.84 | 0.23 | 3.62 | 0.00029 | 0.01191 | 1.79 |
| AL603840.1 | 9.44 | -1.59 | 0.41 | -3.90 | 9.60E-05 | 0.00612 | -3.01 |
| CACHD1 | 257.76 | -0.72 | 0.15 | -4.86 | 1.18E-06 | 0.00028 | -1.65 |
| IL12RB2 | 59.89 | 1.28 | 0.31 | 4.18 | 2.96E-05 | 0.00273 | 2.43 |
| COL24A1 | 79.95 | 1.26 | 0.29 | 4.28 | 1.88E-05 | 0.00203 | 2.40 |
| LMO4 | 564.34 | -0.73 | 0.17 | -4.31 | 1.67E-05 | 0.00192 | -1.66 |
| GBP5 | 82.45 | 1.64 | 0.37 | 4.41 | 1.03E-05 | 0.0014 | 3.12 |
| TGFBR3 | 584.39 | -0.77 | 0.11 | -6.91 | 4.69E-12 | 1.25E-08 | -1.71 |
| F3 | 70.17 | 2.02 | 0.41 | 4.98 | 6.49E-07 | 0.00018 | 4.05 |
| SLC44A3-AS1 | 38.35 | 1.95 | 0.36 | 5.38 | 7.27E-08 | 3.41E-05 | 3.86 |
| SLC44A3 | 24.97 | 2.05 | 0.32 | 6.30 | 2.94E-10 | 4.64E-07 | 4.13 |
| TLCD4 | 281.99 | -1.27 | 0.34 | -3.74 | 0.00018 | 0.00922 | -2.42 |
| GPR88 | 36.87 | 2.19 | 0.40 | 5.46 | 4.74E-08 | 2.68E-05 | 4.57 |
| VCAM1 | 820.58 | 2.08 | 0.31 | 6.71 | 2.01E-11 | 3.87E-08 | 4.23 |
| COL11A1 | 215.25 | 6.18 | 0.90 | 6.87 | 6.49E-12 | 1.39E-08 | 72.59 |
| CSF1 | 469.49 | 0.59 | 0.19 | 3.05 | 0.0023 | 0.04451 | 1.50 |
| KCNA2 | 20.82 | -1.17 | 0.37 | -3.13 | 0.00176 | 0.03826 | -2.25 |
| CD53 | 266.38 | 0.90 | 0.24 | 3.68 | 0.00024 | 0.01076 | 1.87 |
| C1orf162 | 99.39 | 0.77 | 0.26 | 2.99 | 0.00275 | 0.04983 | 1.70 |
| TMIGD3 | 15.52 | 2.00 | 0.49 | 4.05 | 5.06E-05 | 0.00389 | 3.99 |
| PTPN22 | 87.58 | 1.14 | 0.21 | 5.41 | 6.25E-08 | 3.16E-05 | 2.21 |
| TSPAN2 | 48.70 | 1.28 | 0.24 | 5.35 | 8.71E-08 | 3.81E-05 | 2.42 |
| CD2 | 38.94 | 1.37 | 0.42 | 3.22 | 0.00129 | 0.03148 | 2.58 |
| TBX15 | 105.65 | 0.61 | 0.20 | 3.01 | 0.00263 | 0.0486 | 1.52 |
| PHGDH | 101.83 | -0.96 | 0.22 | -4.35 | 1.34E-05 | 0.00168 | -1.94 |
| FCGR1B | 28.96 | 1.49 | 0.35 | 4.29 | 1.79E-05 | 0.00197 | 2.81 |
| FCGR1CP | 22.72 | 1.22 | 0.38 | 3.17 | 0.00153 | 0.03454 | 2.33 |
| FCGR1A | 102.24 | 1.99 | 0.42 | 4.71 | 2.48E-06 | 0.00049 | 3.96 |
| SV2A | 87.96 | -0.65 | 0.18 | -3.66 | 0.00026 | 0.01136 | -1.56 |
| CTSS | 619.80 | 0.83 | 0.27 | 3.13 | 0.00174 | 0.03788 | 1.78 |
| SELENBP1 | 316.39 | -0.87 | 0.19 | -4.49 | 6.97E-06 | 0.00107 | -1.82 |
| TCHH | 7.18 | 1.66 | 0.54 | 3.06 | 0.00224 | 0.04384 | 3.16 |
| SH2D2A | 7.46 | 1.87 | 0.60 | 3.12 | 0.00183 | 0.03884 | 3.65 |
| PYHIN1 | 11.08 | 1.91 | 0.59 | 3.24 | 0.00118 | 0.03003 | 3.76 |
| FCRL6 | 3.66 | 3.92 | 1.06 | 3.70 | 0.00021 | 0.01005 | 15.14 |
| SLAMF8 | 113.01 | 2.22 | 0.42 | 5.29 | 1.24E-07 | 4.95E-05 | 4.67 |
| SLAMF6 | 20.15 | 1.99 | 0.50 | 3.98 | 6.82E-05 | 0.00486 | 3.98 |
| CD84 | 389.53 | 1.10 | 0.23 | 4.84 | 1.31E-06 | 0.0003 | 2.14 |
| SLAMF1 | 10.79 | 1.97 | 0.59 | 3.35 | 0.00081 | 0.02385 | 3.92 |
| CD48 | 31.08 | 1.69 | 0.46 | 3.69 | 0.00023 | 0.01049 | 3.23 |
| ARHGAP30 | 183.70 | 0.84 | 0.25 | 3.42 | 0.00062 | 0.01977 | 1.79 |
| NECTIN4 | 17.84 | 1.32 | 0.39 | 3.38 | 0.00072 | 0.02177 | 2.49 |
| FCGR2A | 998.86 | 1.06 | 0.25 | 4.29 | 1.78E-05 | 0.00197 | 2.09 |
| FCGR3A | 576.91 | 1.47 | 0.37 | 3.92 | 8.98E-05 | 0.00586 | 2.76 |
| OLFML2B | 617.79 | 0.73 | 0.23 | 3.24 | 0.00118 | 0.03003 | 1.66 |
| ILDR2 | 20.44 | 2.28 | 0.41 | 5.63 | 1.79E-08 | 1.11E-05 | 4.87 |
| STYXL2 | 17.16 | 1.17 | 0.37 | 3.12 | 0.0018 | 0.03849 | 2.25 |
| MYOC | 124.51 | -1.55 | 0.52 | -3.01 | 0.00265 | 0.04866 | -2.93 |
| TNFSF18 | 5.59 | 3.43 | 0.83 | 4.13 | 3.70E-05 | 0.00319 | 10.81 |
| TNN | 8.53 | 2.46 | 0.61 | 4.02 | 5.77E-05 | 0.00424 | 5.51 |
| TNR | 20.12 | 2.77 | 0.54 | 5.17 | 2.31E-07 | 8.07E-05 | 6.83 |
| AL365357.1 | 16.26 | 4.23 | 1.12 | 3.79 | 0.00015 | 0.00825 | 18.75 |
| NPL | 155.10 | 0.93 | 0.22 | 4.25 | 2.12E-05 | 0.0022 | 1.91 |
| PRG4 | 679.76 | 3.35 | 0.35 | 9.54 | 1.47E-21 | 2.82E-17 | 10.19 |
| PACERR | 8.96 | -1.12 | 0.34 | -3.27 | 0.00109 | 0.02834 | -2.17 |
| PLA2G4A | 219.25 | -0.88 | 0.22 | -4.04 | 5.37E-05 | 0.00408 | -1.84 |
| RGS13 | 6.81 | 2.74 | 0.61 | 4.49 | 7.01E-06 | 0.00107 | 6.68 |
| PTPRC | 677.15 | 0.78 | 0.25 | 3.18 | 0.00146 | 0.03384 | 1.72 |
| PTPN7 | 29.21 | 1.75 | 0.51 | 3.42 | 0.00063 | 0.02007 | 3.37 |
| BTG2 | 968.96 | -0.83 | 0.25 | -3.28 | 0.00103 | 0.02748 | -1.78 |
| LRRN2 | 12.51 | 2.23 | 0.53 | 4.18 | 2.95E-05 | 0.00273 | 4.69 |
| RHEX | 4.83 | 3.23 | 1.03 | 3.12 | 0.00181 | 0.03856 | 9.37 |
| IKBKE | 49.47 | 0.93 | 0.27 | 3.39 | 0.0007 | 0.02152 | 1.91 |
| AL591846.1 | 12.81 | 5.04 | 1.46 | 3.45 | 0.00057 | 0.01874 | 32.98 |
| FCMR | 14.53 | 2.20 | 0.71 | 3.09 | 0.00198 | 0.04031 | 4.59 |
| CR1 | 213.23 | 1.76 | 0.49 | 3.56 | 0.00038 | 0.01406 | 3.38 |
| G0S2 | 81.50 | -0.95 | 0.31 | -3.04 | 0.00237 | 0.04561 | -1.93 |
| IRF6 | 186.81 | -1.13 | 0.22 | -5.21 | 1.90E-07 | 7.18E-05 | -2.19 |
| KCNK2 | 11.81 | 3.53 | 1.01 | 3.48 | 0.0005 | 0.01702 | 11.51 |
| DNAH14 | 21.34 | 1.16 | 0.35 | 3.31 | 0.00092 | 0.02562 | 2.24 |
| SIPA1L2 | 204.91 | 0.68 | 0.20 | 3.42 | 0.00064 | 0.02027 | 1.60 |
| ACTN2 | 116.14 | -0.68 | 0.22 | -3.02 | 0.00256 | 0.04773 | -1.60 |
| RYR2 | 369.89 | -0.86 | 0.28 | -3.12 | 0.00179 | 0.03849 | -1.82 |
| MATN3 | 16.85 | 1.44 | 0.46 | 3.12 | 0.0018 | 0.03851 | 2.71 |
| SDC1 | 42.23 | 2.27 | 0.65 | 3.51 | 0.00046 | 0.01615 | 4.83 |
| CGREF1 | 90.99 | 1.06 | 0.29 | 3.71 | 0.00021 | 0.00995 | 2.08 |
| PLB1 | 105.33 | 0.76 | 0.22 | 3.39 | 0.0007 | 0.02142 | 1.69 |
| LBH | 475.46 | 0.83 | 0.24 | 3.46 | 0.00054 | 0.01804 | 1.78 |
| FSHR | 10.37 | -1.49 | 0.39 | -3.77 | 0.00016 | 0.00852 | -2.80 |
| EFEMP1 | 8,320.29 | 0.64 | 0.18 | 3.56 | 0.00037 | 0.014 | 1.55 |
| AC092155.1 | 3.49 | -2.50 | 0.69 | -3.65 | 0.00027 | 0.01149 | -5.66 |
| PLEK | 287.04 | 1.05 | 0.27 | 3.82 | 0.00014 | 0.00772 | 2.07 |
| ARHGAP25 | 99.20 | 0.78 | 0.21 | 3.73 | 0.00019 | 0.00944 | 1.71 |
| PAIP2B | 77.77 | -1.04 | 0.26 | -3.92 | 8.76E-05 | 0.00579 | -2.05 |
| CAPG | 330.58 | 1.49 | 0.34 | 4.36 | 1.32E-05 | 0.00167 | 2.81 |
| ST3GAL5 | 195.32 | -0.81 | 0.26 | -3.14 | 0.00168 | 0.03693 | -1.75 |
| CD8A | 17.63 | 2.18 | 0.62 | 3.49 | 0.00048 | 0.01679 | 4.54 |
| IGKC | 5,923.50 | 3.58 | 1.10 | 3.26 | 0.00113 | 0.02889 | 11.94 |
| IGKV1-5 | 194.32 | 4.09 | 1.32 | 3.10 | 0.00195 | 0.04015 | 17.01 |
| IGKV3-11 | 340.37 | 4.48 | 1.34 | 3.34 | 0.00083 | 0.02405 | 22.32 |
| IGKV3-15 | 55.92 | 4.95 | 1.65 | 3.01 | 0.00262 | 0.04848 | 31.01 |
| IGKV1-16 | 12.67 | 5.67 | 1.57 | 3.62 | 0.00029 | 0.01193 | 51.04 |
| FAHD2B | 36.81 | -0.70 | 0.21 | -3.32 | 0.0009 | 0.0253 | -1.62 |
| ZAP70 | 35.17 | 1.59 | 0.45 | 3.51 | 0.00044 | 0.01583 | 3.00 |
| SLC20A1 | 709.97 | 1.04 | 0.18 | 5.93 | 3.12E-09 | 2.86E-06 | 2.06 |
| IL1RN | 23.14 | 3.58 | 0.92 | 3.88 | 0.00011 | 0.00646 | 11.95 |
| STEAP3 | 348.65 | 0.76 | 0.25 | 3.07 | 0.00216 | 0.04273 | 1.69 |
| ARHGAP15 | 66.97 | 0.91 | 0.24 | 3.72 | 0.0002 | 0.00982 | 1.87 |
| CACNB4 | 133.42 | 1.00 | 0.26 | 3.80 | 0.00014 | 0.00801 | 2.00 |
| DPP4 | 41.60 | 1.88 | 0.43 | 4.41 | 1.03E-05 | 0.0014 | 3.69 |
| FAP | 603.10 | 1.12 | 0.31 | 3.61 | 0.0003 | 0.0122 | 2.18 |
| GALNT3 | 33.51 | 3.03 | 0.52 | 5.79 | 7.06E-09 | 5.66E-06 | 8.17 |
| SCN7A | 238.79 | -1.56 | 0.50 | -3.13 | 0.00174 | 0.03788 | -2.95 |
| AC010680.1 | 8.64 | -1.19 | 0.38 | -3.12 | 0.0018 | 0.03849 | -2.29 |
| ITGA4 | 277.86 | 0.80 | 0.22 | 3.68 | 0.00023 | 0.01069 | 1.74 |
| FAM171B | 226.18 | -0.63 | 0.20 | -3.10 | 0.0019 | 0.03982 | -1.55 |
| GULP1 | 173.24 | -0.73 | 0.20 | -3.71 | 0.00021 | 0.00993 | -1.66 |
| COL5A2 | 3,853.40 | 0.80 | 0.27 | 3.03 | 0.00246 | 0.04663 | 1.75 |
| C2orf88 | 71.44 | -1.13 | 0.35 | -3.18 | 0.00148 | 0.03393 | -2.18 |
| STAT1 | 1,131.03 | 0.63 | 0.17 | 3.65 | 0.00026 | 0.01142 | 1.55 |
| SATB2 | 75.48 | 1.01 | 0.22 | 4.62 | 3.82E-06 | 0.00067 | 2.01 |
| AOX1 | 262.80 | -0.59 | 0.18 | -3.32 | 0.00089 | 0.02512 | -1.50 |
| CD28 | 176.21 | 1.57 | 0.27 | 5.90 | 3.58E-09 | 3.13E-06 | 2.96 |
| NRP2 | 571.68 | 0.71 | 0.17 | 4.10 | 4.22E-05 | 0.00353 | 1.64 |
| ACADL | 72.02 | -0.64 | 0.19 | -3.40 | 0.00068 | 0.0212 | -1.56 |
| FN1 | 102,162.74 | 1.49 | 0.25 | 6.03 | 1.61E-09 | 1.72E-06 | 2.82 |
| IGFBP2 | 489.70 | 2.42 | 0.47 | 5.14 | 2.68E-07 | 8.87E-05 | 5.35 |
| IGFBP5 | 10,402.36 | -0.80 | 0.23 | -3.45 | 0.00056 | 0.01866 | -1.74 |
| SLC11A1 | 117.04 | 1.69 | 0.56 | 3.02 | 0.00255 | 0.0476 | 3.22 |
| PRKAG3 | 11.38 | 1.64 | 0.47 | 3.47 | 0.00052 | 0.01753 | 3.11 |
| SGPP2 | 7.47 | 2.50 | 0.64 | 3.93 | 8.43E-05 | 0.00563 | 5.66 |
| SCG2 | 305.64 | 1.94 | 0.36 | 5.44 | 5.23E-08 | 2.88E-05 | 3.84 |
| DOCK10 | 302.32 | 0.88 | 0.26 | 3.39 | 0.00069 | 0.02122 | 1.85 |
| IRS1 | 267.35 | -0.65 | 0.15 | -4.25 | 2.17E-05 | 0.00222 | -1.57 |
| SLC19A3 | 15.83 | -1.11 | 0.31 | -3.64 | 0.00027 | 0.01156 | -2.17 |
| NGEF | 129.11 | 1.01 | 0.31 | 3.24 | 0.00119 | 0.03003 | 2.01 |
| INPP5D | 221.44 | 0.97 | 0.25 | 3.90 | 9.51E-05 | 0.00608 | 1.96 |
| ARL4C | 235.14 | 0.73 | 0.23 | 3.20 | 0.00136 | 0.0327 | 1.66 |
| AC112721.2 | 3.23 | 3.63 | 1.14 | 3.20 | 0.00138 | 0.03302 | 12.39 |
| RNPEPL1 | 220.57 | 0.59 | 0.19 | 3.19 | 0.00143 | 0.03358 | 1.51 |
| KIF1A | 36.94 | -1.19 | 0.38 | -3.14 | 0.00167 | 0.03683 | -2.29 |
| SNED1 | 976.69 | 0.80 | 0.22 | 3.55 | 0.00039 | 0.01434 | 1.74 |
| LRRN1 | 30.33 | 0.90 | 0.27 | 3.35 | 0.00082 | 0.02394 | 1.87 |
| LMCD1-AS1 | 15.79 | -1.04 | 0.32 | -3.29 | 0.00102 | 0.02716 | -2.06 |
| HRH1 | 214.21 | 0.71 | 0.19 | 3.73 | 0.00019 | 0.00944 | 1.64 |
| ZNF385D | 89.00 | 0.81 | 0.22 | 3.62 | 0.0003 | 0.01214 | 1.75 |
| MYD88 | 110.12 | 0.97 | 0.32 | 3.07 | 0.00212 | 0.0422 | 1.96 |
| VIPR1 | 105.02 | -1.13 | 0.37 | -3.01 | 0.00263 | 0.0486 | -2.18 |
| CDCP1 | 54.47 | 1.47 | 0.38 | 3.82 | 0.00013 | 0.00766 | 2.76 |
| SLC6A20 | 17.17 | -1.73 | 0.53 | -3.29 | 0.00099 | 0.02672 | -3.32 |
| CCR1 | 169.22 | 1.30 | 0.29 | 4.46 | 8.25E-06 | 0.00118 | 2.46 |
| STAB1 | 4,669.38 | 0.71 | 0.21 | 3.37 | 0.00074 | 0.02227 | 1.63 |
| ITIH3 | 52.07 | 1.49 | 0.47 | 3.17 | 0.00151 | 0.03426 | 2.80 |
| PRKCD | 92.03 | 0.69 | 0.20 | 3.52 | 0.00043 | 0.01545 | 1.62 |
| AC115282.2 | 4.93 | -2.41 | 0.78 | -3.10 | 0.00194 | 0.04004 | -5.32 |
| CACNA2D3 | 28.87 | -0.79 | 0.25 | -3.18 | 0.00148 | 0.03393 | -1.73 |
| CFAP20DC | 15.46 | -0.99 | 0.32 | -3.10 | 0.00192 | 0.03996 | -1.98 |
| CADM2 | 118.57 | -1.38 | 0.36 | -3.82 | 0.00014 | 0.00772 | -2.60 |
| COL8A1 | 2,195.58 | 1.02 | 0.24 | 4.28 | 1.86E-05 | 0.00203 | 2.03 |
| CD96 | 31.71 | 1.98 | 0.53 | 3.71 | 0.00021 | 0.00993 | 3.94 |
| CCDC80 | 7,267.24 | 0.98 | 0.26 | 3.81 | 0.00014 | 0.00774 | 1.97 |
| AC026341.3 | 18.32 | 1.60 | 0.50 | 3.18 | 0.00146 | 0.03384 | 3.04 |
| GAP43 | 239.80 | 0.93 | 0.29 | 3.16 | 0.00159 | 0.0357 | 1.90 |
| CFAP91 | 35.57 | -0.89 | 0.30 | -2.99 | 0.00276 | 0.04992 | -1.85 |
| AC092910.3 | 15.09 | -1.10 | 0.33 | -3.29 | 0.00101 | 0.027 | -2.15 |
| STXBP5L | 86.00 | -1.50 | 0.34 | -4.44 | 9.04E-06 | 0.00128 | -2.83 |
| CD86 | 95.83 | 0.99 | 0.27 | 3.71 | 0.0002 | 0.00989 | 1.99 |
| PARP15 | 51.79 | 1.39 | 0.35 | 3.95 | 7.90E-05 | 0.00533 | 2.62 |
| COL6A6 | 466.87 | -1.47 | 0.48 | -3.09 | 0.00203 | 0.04091 | -2.77 |
| TF | 225.25 | -1.69 | 0.51 | -3.29 | 0.00099 | 0.02672 | -3.22 |
| AC010207.1 | 6.17 | -1.76 | 0.55 | -3.20 | 0.00139 | 0.03308 | -3.38 |
| RBP1 | 85.43 | -1.00 | 0.22 | -4.48 | 7.63E-06 | 0.00113 | -2.00 |
| CLSTN2 | 506.91 | -1.70 | 0.33 | -5.12 | 3.07E-07 | 9.54E-05 | -3.25 |
| CPA3 | 62.86 | 2.25 | 0.60 | 3.78 | 0.00016 | 0.0084 | 4.76 |
| IGSF10 | 484.01 | -1.66 | 0.42 | -3.92 | 8.89E-05 | 0.00584 | -3.15 |
| SUCNR1 | 22.57 | 1.12 | 0.35 | 3.24 | 0.00121 | 0.03025 | 2.17 |
| P2RY1 | 158.05 | -0.86 | 0.22 | -3.88 | 0.00011 | 0.00647 | -1.81 |
| PLCH1 | 130.11 | -1.91 | 0.34 | -5.57 | 2.58E-08 | 1.55E-05 | -3.75 |
| SHOX2 | 6.55 | 1.96 | 0.51 | 3.88 | 0.0001 | 0.00645 | 3.89 |
| AC080013.1 | 85.68 | -0.92 | 0.28 | -3.31 | 0.00094 | 0.02592 | -1.90 |
| PPM1L | 363.69 | -0.63 | 0.15 | -4.25 | 2.17E-05 | 0.00222 | -1.55 |
| CLDN11 | 328.08 | -0.95 | 0.18 | -5.35 | 8.58E-08 | 3.81E-05 | -1.93 |
| SLC7A14-AS1 | 29.85 | -1.06 | 0.33 | -3.26 | 0.0011 | 0.02855 | -2.09 |
| TNFSF10 | 327.91 | 0.73 | 0.22 | 3.26 | 0.00113 | 0.02903 | 1.66 |
| NLGN1 | 41.98 | -0.93 | 0.25 | -3.73 | 0.00019 | 0.00944 | -1.91 |
| AC117453.1 | 23.29 | 2.48 | 0.54 | 4.58 | 4.56E-06 | 0.00075 | 5.57 |
| KLHL6 | 78.65 | 1.23 | 0.33 | 3.66 | 0.00025 | 0.01117 | 2.34 |
| AC007920.2 | 19.47 | -1.18 | 0.26 | -4.51 | 6.54E-06 | 0.00102 | -2.27 |
| BCL6 | 632.78 | -0.72 | 0.16 | -4.57 | 4.98E-06 | 0.00079 | -1.65 |
| TPRG1 | 165.27 | -0.77 | 0.21 | -3.61 | 0.00031 | 0.0123 | -1.71 |
| LRRC15 | 203.65 | 3.28 | 0.69 | 4.75 | 2.00E-06 | 0.00042 | 9.73 |
| ZNF595 | 104.08 | -0.63 | 0.20 | -3.09 | 0.00203 | 0.04094 | -1.54 |
| LAP3 | 452.02 | 0.71 | 0.21 | 3.40 | 0.00068 | 0.0212 | 1.64 |
| LGI2 | 121.67 | 0.68 | 0.22 | 3.06 | 0.00223 | 0.04374 | 1.60 |
| DTHD1 | 4.68 | 2.87 | 0.89 | 3.23 | 0.00122 | 0.03043 | 7.32 |
| ATP8A1 | 431.58 | -1.19 | 0.25 | -4.73 | 2.25E-06 | 0.00046 | -2.28 |
| LNX1 | 79.00 | 0.79 | 0.23 | 3.52 | 0.00043 | 0.0155 | 1.73 |
| KIT | 62.92 | 1.83 | 0.40 | 4.60 | 4.22E-06 | 0.00071 | 3.54 |
| JCHAIN | 649.09 | 3.44 | 1.10 | 3.12 | 0.00179 | 0.03849 | 10.89 |
| CXCL5 | 24.13 | 5.63 | 1.20 | 4.71 | 2.49E-06 | 0.00049 | 49.65 |
| CXCL3 | 5.66 | 2.02 | 0.64 | 3.16 | 0.00159 | 0.0357 | 4.06 |
| BTC | 37.68 | -0.93 | 0.29 | -3.24 | 0.0012 | 0.0301 | -1.91 |
| CXCL10 | 32.14 | 2.31 | 0.55 | 4.18 | 2.96E-05 | 0.00273 | 4.95 |
| CXCL11 | 3.74 | 3.30 | 1.05 | 3.16 | 0.00159 | 0.03571 | 9.86 |
| CXCL13 | 5.58 | 4.20 | 1.40 | 3.00 | 0.00268 | 0.04915 | 18.36 |
| ANXA3 | 108.04 | -0.75 | 0.24 | -3.08 | 0.00209 | 0.04182 | -1.68 |
| LINC01094 | 74.22 | 1.01 | 0.27 | 3.71 | 0.00021 | 0.00993 | 2.01 |
| LINC01088 | 158.32 | -0.79 | 0.25 | -3.20 | 0.0014 | 0.03313 | -1.73 |
| BMP3 | 7.30 | 6.34 | 1.65 | 3.84 | 0.00012 | 0.00732 | 80.85 |
| PLAC8 | 8.30 | 2.23 | 0.68 | 3.26 | 0.0011 | 0.02854 | 4.70 |
| PTPN13 | 1,310.16 | -0.62 | 0.15 | -4.08 | 4.49E-05 | 0.00363 | -1.53 |
| DMP1 | 5.33 | 4.96 | 1.22 | 4.08 | 4.51E-05 | 0.00363 | 31.10 |
| IBSP | 78.16 | 5.52 | 1.05 | 5.27 | 1.38E-07 | 5.41E-05 | 45.78 |
| SPP1 | 2,847.46 | 4.66 | 0.91 | 5.13 | 2.82E-07 | 9.06E-05 | 25.28 |
| GRID2 | 16.55 | -1.96 | 0.54 | -3.63 | 0.00028 | 0.01169 | -3.89 |
| UNC5C | 697.87 | -0.59 | 0.15 | -3.96 | 7.62E-05 | 0.0052 | -1.51 |
| AC106881.1 | 21.50 | -0.94 | 0.25 | -3.73 | 0.00019 | 0.00944 | -1.91 |
| CFI | 27.45 | 1.22 | 0.32 | 3.79 | 0.00015 | 0.00815 | 2.34 |
| SMIM43 | 11.24 | 2.70 | 0.73 | 3.70 | 0.00021 | 0.01005 | 6.48 |
| INTU | 310.12 | -0.60 | 0.17 | -3.50 | 0.00046 | 0.01645 | -1.52 |
| NR3C2 | 198.39 | -0.59 | 0.17 | -3.48 | 0.00049 | 0.01702 | -1.50 |
| AC106865.1 | 23.47 | 1.93 | 0.63 | 3.05 | 0.00232 | 0.04485 | 3.82 |
| TLR2 | 266.26 | 0.83 | 0.23 | 3.59 | 0.00034 | 0.01292 | 1.77 |
| TDO2 | 17.48 | 4.48 | 1.04 | 4.31 | 1.64E-05 | 0.00191 | 22.28 |
| GRIA2 | 6.48 | -2.38 | 0.65 | -3.67 | 0.00024 | 0.01079 | -5.20 |
| MARCHF1 | 220.13 | 0.77 | 0.22 | 3.49 | 0.00048 | 0.01676 | 1.71 |
| APELA | 7.70 | 4.90 | 1.14 | 4.29 | 1.79E-05 | 0.00197 | 29.83 |
| HAND2 | 216.18 | -0.75 | 0.25 | -3.02 | 0.00254 | 0.04756 | -1.68 |
| TENM3 | 80.75 | -1.11 | 0.27 | -4.08 | 4.48E-05 | 0.00363 | -2.16 |
| STOX2 | 100.13 | -0.66 | 0.17 | -3.90 | 9.82E-05 | 0.00619 | -1.58 |
| TPPP | 117.33 | -1.01 | 0.33 | -3.03 | 0.00241 | 0.04587 | -2.02 |
| ZDHHC11B | 64.19 | -2.10 | 0.42 | -4.95 | 7.43E-07 | 0.0002 | -4.29 |
| ZDHHC11 | 33.29 | -1.65 | 0.36 | -4.60 | 4.17E-06 | 0.00071 | -3.14 |
| AC010343.3 | 6.26 | 4.87 | 1.07 | 4.55 | 5.36E-06 | 0.00084 | 29.15 |
| GDNF | 14.95 | 1.32 | 0.37 | 3.61 | 0.0003 | 0.01219 | 2.50 |
| FYB1 | 262.75 | 1.14 | 0.23 | 4.97 | 6.77E-07 | 0.00019 | 2.20 |
| DAB2 | 1,695.15 | 0.61 | 0.16 | 3.90 | 9.81E-05 | 0.00619 | 1.53 |
| FST | 262.13 | 0.90 | 0.29 | 3.06 | 0.00223 | 0.04374 | 1.86 |
| GZMK | 19.95 | 2.31 | 0.64 | 3.61 | 0.00031 | 0.01227 | 4.96 |
| GZMA | 23.08 | 2.32 | 0.50 | 4.62 | 3.91E-06 | 0.00068 | 5.01 |
| HTR1A | 7.93 | -1.97 | 0.63 | -3.14 | 0.00167 | 0.03684 | -3.91 |
| NAIP | 61.76 | 1.07 | 0.28 | 3.83 | 0.00013 | 0.00751 | 2.10 |
| NR2F1-AS1 | 302.39 | -0.77 | 0.26 | -3.02 | 0.00251 | 0.0472 | -1.71 |
| NR2F1 | 66.67 | -0.83 | 0.22 | -3.76 | 0.00017 | 0.00876 | -1.78 |
| MCTP1 | 101.27 | 0.88 | 0.29 | 3.00 | 0.00266 | 0.04876 | 1.83 |
| RHOBTB3 | 413.39 | -0.67 | 0.21 | -3.20 | 0.00137 | 0.03275 | -1.59 |
| HSPD1P11 | 14.71 | -1.09 | 0.36 | -3.00 | 0.00273 | 0.04961 | -2.13 |
| ST8SIA4 | 154.23 | 0.89 | 0.21 | 4.31 | 1.61E-05 | 0.0019 | 1.85 |
| PRDM6 | 21.59 | 1.02 | 0.32 | 3.23 | 0.00123 | 0.03058 | 2.03 |
| PDLIM4 | 42.94 | 1.05 | 0.35 | 3.04 | 0.0024 | 0.04587 | 2.07 |
| MIR3936HG | 43.47 | -0.90 | 0.29 | -3.12 | 0.00179 | 0.03849 | -1.87 |
| CXCL14 | 216.28 | 2.15 | 0.60 | 3.62 | 0.0003 | 0.01217 | 4.44 |
| SPOCK1 | 304.63 | 1.54 | 0.33 | 4.67 | 2.95E-06 | 0.00056 | 2.90 |
| CD14 | 1,122.63 | 0.91 | 0.24 | 3.74 | 0.00019 | 0.00927 | 1.88 |
| PCDHGB4 | 49.05 | -0.65 | 0.21 | -3.18 | 0.00146 | 0.03383 | -1.57 |
| PCDH1 | 204.66 | 1.03 | 0.30 | 3.40 | 0.00068 | 0.02109 | 2.04 |
| PCDH12 | 235.58 | 0.82 | 0.27 | 3.03 | 0.00246 | 0.04663 | 1.77 |
| STK32A | 162.21 | -0.66 | 0.20 | -3.26 | 0.00111 | 0.02861 | -1.58 |
| CSF1R | 1,034.41 | 0.91 | 0.29 | 3.18 | 0.00149 | 0.03404 | 1.88 |
| CD74 | 7,848.91 | 1.14 | 0.27 | 4.26 | 2.00E-05 | 0.00214 | 2.20 |
| GRIA1 | 6.25 | 5.61 | 1.28 | 4.38 | 1.21E-05 | 0.00153 | 48.99 |
| SGCD | 336.71 | 0.62 | 0.19 | 3.21 | 0.00133 | 0.03208 | 1.54 |
| TIMD4 | 44.16 | 2.69 | 0.64 | 4.17 | 3.04E-05 | 0.00277 | 6.44 |
| DOCK2 | 413.13 | 0.72 | 0.21 | 3.48 | 0.00049 | 0.01702 | 1.65 |
| LCP2 | 193.07 | 1.21 | 0.27 | 4.47 | 7.99E-06 | 0.00116 | 2.32 |
| SH3PXD2B | 372.00 | 0.60 | 0.18 | 3.43 | 0.00061 | 0.01958 | 1.52 |
| HRH2 | 45.76 | 1.01 | 0.31 | 3.22 | 0.00129 | 0.03148 | 2.01 |
| HK3 | 79.65 | 1.29 | 0.36 | 3.57 | 0.00036 | 0.01355 | 2.44 |
| RGS14 | 40.43 | 1.09 | 0.27 | 3.97 | 7.34E-05 | 0.00508 | 2.13 |
| DOK3 | 58.13 | 1.47 | 0.36 | 4.06 | 4.93E-05 | 0.00386 | 2.77 |
| TUBB2B | 64.20 | -1.31 | 0.32 | -4.13 | 3.68E-05 | 0.00319 | -2.47 |
| F13A1 | 4,679.05 | 0.96 | 0.29 | 3.34 | 0.00083 | 0.02411 | 1.95 |
| LY86 | 37.25 | 1.20 | 0.38 | 3.13 | 0.00177 | 0.03836 | 2.30 |
| ADTRP | 9.81 | 1.42 | 0.41 | 3.45 | 0.00056 | 0.01856 | 2.68 |
| PHACTR1 | 49.89 | 0.70 | 0.19 | 3.64 | 0.00027 | 0.01158 | 1.63 |
| CASC15 | 257.11 | -0.84 | 0.21 | -3.95 | 7.84E-05 | 0.00531 | -1.79 |
| H1-3 | 98.12 | 0.98 | 0.29 | 3.42 | 0.00062 | 0.01977 | 1.97 |
| H4C12 | 5.17 | 1.72 | 0.54 | 3.18 | 0.00149 | 0.03404 | 3.30 |
| TNF | 12.98 | 1.37 | 0.43 | 3.17 | 0.00151 | 0.03426 | 2.59 |
| C2 | 121.80 | 0.90 | 0.21 | 4.30 | 1.73E-05 | 0.00195 | 1.87 |
| CFB | 52.76 | 0.70 | 0.20 | 3.49 | 0.00049 | 0.01698 | 1.62 |
| C4A | 91.56 | 0.99 | 0.32 | 3.12 | 0.00183 | 0.03876 | 1.98 |
| GPSM3 | 84.22 | 0.90 | 0.27 | 3.30 | 0.00096 | 0.02607 | 1.87 |
| HLA-DRA | 3,926.94 | 0.99 | 0.28 | 3.55 | 0.00039 | 0.01434 | 1.99 |
| HLA-DRB1 | 2,439.55 | 1.09 | 0.27 | 3.98 | 6.99E-05 | 0.00493 | 2.13 |
| HLA-DQA1 | 626.13 | 1.76 | 0.33 | 5.40 | 6.74E-08 | 3.24E-05 | 3.40 |
| HLA-DQB1 | 747.23 | 2.17 | 0.49 | 4.42 | 1.00E-05 | 0.00138 | 4.50 |
| HLA-DMB | 285.37 | 0.83 | 0.23 | 3.54 | 0.0004 | 0.0148 | 1.78 |
| HLA-DMA | 350.71 | 0.79 | 0.21 | 3.74 | 0.00019 | 0.00927 | 1.73 |
| HLA-DPA1 | 2,276.01 | 0.96 | 0.25 | 3.92 | 9.03E-05 | 0.00586 | 1.94 |
| HLA-DPB1 | 1,242.87 | 0.85 | 0.25 | 3.41 | 0.00066 | 0.02075 | 1.80 |
| SCUBE3 | 28.66 | 2.29 | 0.48 | 4.76 | 1.97E-06 | 0.00042 | 4.89 |
| MAPK13 | 43.00 | 2.06 | 0.33 | 6.20 | 5.50E-10 | 6.61E-07 | 4.17 |
| KCNK17 | 133.94 | -0.93 | 0.20 | -4.73 | 2.29E-06 | 0.00046 | -1.90 |
| TREM2 | 67.28 | 1.41 | 0.37 | 3.83 | 0.00013 | 0.00754 | 2.66 |
| TREM1 | 61.33 | 2.22 | 0.67 | 3.30 | 0.00096 | 0.02607 | 4.67 |
| HMGCLL1 | 55.23 | -1.41 | 0.34 | -4.18 | 2.94E-05 | 0.00273 | -2.66 |
| AL391807.1 | 13.40 | -1.23 | 0.41 | -3.00 | 0.00272 | 0.04944 | -2.35 |
| COL12A1 | 5,250.82 | 0.85 | 0.19 | 4.40 | 1.07E-05 | 0.00141 | 1.80 |
| IRAK1BP1 | 90.06 | -0.62 | 0.17 | -3.70 | 0.00021 | 0.01002 | -1.54 |
| TBX18 | 357.80 | -0.78 | 0.26 | -3.02 | 0.00255 | 0.04764 | -1.72 |
| CNR1 | 130.37 | -0.97 | 0.24 | -3.98 | 7.02E-05 | 0.00493 | -1.96 |
| EPHA7 | 59.65 | -0.98 | 0.32 | -3.03 | 0.00242 | 0.04598 | -1.98 |
| CRYBG1 | 208.99 | 1.09 | 0.25 | 4.41 | 1.05E-05 | 0.0014 | 2.14 |
| SLC22A16 | 2.96 | 2.68 | 0.89 | 3.01 | 0.00263 | 0.0486 | 6.40 |
| SLC16A10 | 83.12 | 1.72 | 0.44 | 3.92 | 8.85E-05 | 0.00583 | 3.30 |
| COL10A1 | 126.01 | 2.85 | 0.85 | 3.34 | 0.00083 | 0.02404 | 7.23 |
| ROS1 | 5.71 | 5.06 | 1.26 | 4.03 | 5.62E-05 | 0.00418 | 33.42 |
| TRDN | 12.71 | -2.05 | 0.47 | -4.33 | 1.50E-05 | 0.00183 | -4.15 |
| TRDN-AS1 | 6.82 | -3.86 | 0.75 | -5.13 | 2.95E-07 | 9.29E-05 | -14.49 |
| NKAIN2 | 18.71 | -2.12 | 0.46 | -4.66 | 3.20E-06 | 0.00058 | -4.36 |
| AL365259.1 | 20.91 | -0.97 | 0.32 | -3.04 | 0.00233 | 0.04495 | -1.95 |
| HEY2 | 218.88 | -0.67 | 0.17 | -4.08 | 4.46E-05 | 0.00363 | -1.60 |
| AL356124.1 | 45.61 | 0.91 | 0.23 | 3.85 | 0.00012 | 0.00694 | 1.87 |
| AL356124.2 | 3.17 | 2.26 | 0.70 | 3.21 | 0.00132 | 0.03191 | 4.79 |
| ARHGAP18 | 433.86 | 0.64 | 0.19 | 3.43 | 0.00061 | 0.01961 | 1.55 |
| TMEM200A | 52.72 | 1.61 | 0.40 | 4.07 | 4.63E-05 | 0.00367 | 3.06 |
| ENPP1 | 399.37 | 2.17 | 0.31 | 6.99 | 2.70E-12 | 1.25E-08 | 4.49 |
| CCN2 | 4,197.71 | 0.88 | 0.23 | 3.88 | 0.0001 | 0.00645 | 1.84 |
| MOXD1 | 36.34 | 1.77 | 0.47 | 3.73 | 0.00019 | 0.00944 | 3.40 |
| AL121933.2 | 10.82 | 1.22 | 0.38 | 3.18 | 0.00147 | 0.03392 | 2.32 |
| PLAGL1 | 255.41 | 0.72 | 0.16 | 4.49 | 7.26E-06 | 0.00109 | 1.65 |
| UST | 233.96 | -0.71 | 0.20 | -3.60 | 0.00032 | 0.0124 | -1.64 |
| AKAP12 | 2,065.26 | -0.60 | 0.14 | -4.14 | 3.42E-05 | 0.003 | -1.52 |
| FNDC1 | 550.50 | 1.81 | 0.43 | 4.22 | 2.48E-05 | 0.00241 | 3.50 |
| LPAL2 | 10.62 | 1.34 | 0.40 | 3.37 | 0.00074 | 0.02235 | 2.54 |
| SMOC2 | 291.53 | 1.71 | 0.28 | 6.11 | 1.02E-09 | 1.16E-06 | 3.28 |
| THBS2 | 6,911.45 | 1.12 | 0.27 | 4.16 | 3.17E-05 | 0.00286 | 2.17 |
| AC215522.2 | 3.68 | 2.39 | 0.75 | 3.18 | 0.00146 | 0.03384 | 5.26 |
| FAM20C | 258.79 | 0.83 | 0.22 | 3.81 | 0.00014 | 0.00786 | 1.78 |
| TTYH3 | 338.36 | 0.86 | 0.27 | 3.13 | 0.00175 | 0.03812 | 1.81 |
| CARD11 | 30.23 | 1.25 | 0.38 | 3.26 | 0.00111 | 0.02857 | 2.39 |
| MPP6 | 384.45 | -0.97 | 0.21 | -4.59 | 4.52E-06 | 0.00075 | -1.96 |
| SNX10 | 54.03 | 1.28 | 0.30 | 4.29 | 1.79E-05 | 0.00197 | 2.43 |
| CPVL | 292.55 | 0.97 | 0.27 | 3.60 | 0.00031 | 0.01239 | 1.96 |
| MTURN | 437.35 | -0.60 | 0.14 | -4.43 | 9.64E-06 | 0.00133 | -1.52 |
| INMT | 1,587.48 | -0.80 | 0.17 | -4.68 | 2.83E-06 | 0.00055 | -1.74 |
| AOAH | 86.47 | 1.09 | 0.31 | 3.53 | 0.00041 | 0.01501 | 2.12 |
| TRG-AS1 | 10.25 | 2.08 | 0.60 | 3.46 | 0.00054 | 0.01809 | 4.22 |
| POU6F2 | 7.80 | 2.59 | 0.74 | 3.48 | 0.0005 | 0.01702 | 6.00 |
| MYO1G | 80.30 | 1.48 | 0.41 | 3.61 | 0.0003 | 0.01219 | 2.78 |
| IGFBP1 | 4.96 | 3.66 | 0.98 | 3.73 | 0.00019 | 0.00944 | 12.68 |
| IKZF1 | 142.19 | 1.29 | 0.30 | 4.21 | 2.50E-05 | 0.00241 | 2.44 |
| LIMK1 | 155.00 | 0.61 | 0.19 | 3.21 | 0.00135 | 0.03247 | 1.52 |
| LAT2 | 73.34 | 1.13 | 0.29 | 3.88 | 0.0001 | 0.00645 | 2.19 |
| PHTF2 | 265.14 | 0.84 | 0.16 | 5.16 | 2.52E-07 | 8.50E-05 | 1.79 |
| SEMA3A | 300.03 | -0.98 | 0.26 | -3.80 | 0.00015 | 0.00814 | -1.97 |
| AC005076.2 | 4.26 | -1.63 | 0.50 | -3.27 | 0.00106 | 0.02802 | -3.08 |
| STEAP1 | 102.10 | 1.47 | 0.23 | 6.46 | 1.06E-10 | 1.86E-07 | 2.78 |
| STEAP2 | 338.22 | 0.78 | 0.19 | 4.11 | 3.91E-05 | 0.00331 | 1.72 |
| PPP1R9A | 39.86 | -1.13 | 0.27 | -4.15 | 3.37E-05 | 0.00297 | -2.19 |
| DLX5 | 4.80 | 5.31 | 1.32 | 4.02 | 5.73E-05 | 0.00422 | 39.64 |
| PILRA | 76.52 | 1.13 | 0.25 | 4.43 | 9.60E-06 | 0.00133 | 2.19 |
| LRRC17 | 367.65 | 0.87 | 0.23 | 3.75 | 0.00018 | 0.00914 | 1.83 |
| PIK3CG | 172.27 | 0.86 | 0.22 | 3.96 | 7.36E-05 | 0.00508 | 1.82 |
| PRKAR2B | 110.84 | -0.62 | 0.20 | -3.08 | 0.00208 | 0.04162 | -1.54 |
| LEP | 24.76 | 1.88 | 0.59 | 3.18 | 0.00148 | 0.03393 | 3.69 |
| ATP6V1F | 142.78 | 0.60 | 0.18 | 3.29 | 0.00101 | 0.027 | 1.52 |
| TSPAN33 | 116.34 | 0.90 | 0.22 | 4.15 | 3.28E-05 | 0.00292 | 1.87 |
| STRIP2 | 11.03 | 1.26 | 0.41 | 3.10 | 0.00194 | 0.04004 | 2.40 |
| DGKI | 206.09 | 1.04 | 0.23 | 4.56 | 5.11E-06 | 0.00081 | 2.05 |
| DENND2A | 166.29 | -0.60 | 0.19 | -3.17 | 0.00153 | 0.03443 | -1.52 |
| TRBC1 | 21.34 | 1.90 | 0.57 | 3.35 | 0.00082 | 0.02395 | 3.74 |
| TRBC2 | 38.94 | 2.10 | 0.55 | 3.79 | 0.00015 | 0.00815 | 4.28 |
| EPHB6 | 114.41 | -0.77 | 0.25 | -3.13 | 0.00173 | 0.03771 | -1.70 |
| GIMAP1 | 51.00 | 0.79 | 0.25 | 3.21 | 0.00134 | 0.03221 | 1.73 |
| MSR1 | 932.46 | 1.00 | 0.28 | 3.63 | 0.00028 | 0.01169 | 2.00 |
| SLC7A2 | 165.42 | 1.73 | 0.34 | 5.03 | 4.98E-07 | 0.00015 | 3.31 |
| PDGFRL | 27.24 | 1.20 | 0.37 | 3.23 | 0.00125 | 0.03094 | 2.30 |
| AC100849.1 | 10.73 | 1.38 | 0.44 | 3.11 | 0.00184 | 0.03892 | 2.60 |
| HR | 65.84 | -0.99 | 0.22 | -4.46 | 8.08E-06 | 0.00116 | -1.98 |
| SLC39A14 | 602.39 | 0.67 | 0.20 | 3.32 | 0.00089 | 0.02512 | 1.59 |
| ADAM28 | 119.56 | 0.94 | 0.27 | 3.54 | 0.0004 | 0.0146 | 1.92 |
| EBF2 | 10.21 | 2.53 | 0.63 | 4.02 | 5.89E-05 | 0.00429 | 5.76 |
| EPHX2 | 111.70 | -0.63 | 0.18 | -3.53 | 0.00042 | 0.01527 | -1.55 |
| SCARA5 | 736.43 | -1.00 | 0.31 | -3.22 | 0.00127 | 0.03141 | -2.00 |
| ZNF395 | 247.48 | -0.68 | 0.13 | -5.16 | 2.49E-07 | 8.50E-05 | -1.61 |
| IDO1 | 6.18 | 3.53 | 0.91 | 3.88 | 0.0001 | 0.00645 | 11.52 |
| LYN | 220.85 | 0.65 | 0.19 | 3.47 | 0.00051 | 0.01744 | 1.57 |
| AC104051.2 | 27.66 | -1.77 | 0.57 | -3.13 | 0.00176 | 0.0382 | -3.42 |
| FAM110B | 149.26 | -0.70 | 0.20 | -3.49 | 0.00048 | 0.01676 | -1.62 |
| PKIA | 49.31 | -0.99 | 0.26 | -3.80 | 0.00014 | 0.00793 | -1.98 |
| STMN2 | 78.27 | 3.95 | 0.56 | 7.07 | 1.53E-12 | 9.84E-09 | 15.42 |
| MATN2 | 2,205.57 | -0.91 | 0.22 | -4.09 | 4.24E-05 | 0.00353 | -1.88 |
| BAALC | 5.40 | 2.41 | 0.71 | 3.41 | 0.00065 | 0.02057 | 5.32 |
| BAALC-AS1 | 53.30 | 0.71 | 0.23 | 3.12 | 0.00183 | 0.03876 | 1.63 |
| CTHRC1 | 241.05 | 1.33 | 0.36 | 3.68 | 0.00023 | 0.0106 | 2.51 |
| RSPO2 | 63.73 | 1.34 | 0.38 | 3.49 | 0.00049 | 0.01698 | 2.53 |
| SAMD12 | 32.03 | -0.96 | 0.31 | -3.06 | 0.00222 | 0.04359 | -1.94 |
| TNFRSF11B | 779.98 | 1.84 | 0.27 | 6.90 | 5.20E-12 | 1.25E-08 | 3.57 |
| CCN3 | 547.01 | 1.15 | 0.28 | 4.08 | 4.54E-05 | 0.00363 | 2.22 |
| COL14A1 | 9,092.95 | 0.63 | 0.15 | 4.28 | 1.84E-05 | 0.00201 | 1.54 |
| AC100858.2 | 8.87 | 1.24 | 0.41 | 3.03 | 0.00241 | 0.04587 | 2.36 |
| LINC00861 | 14.87 | 3.11 | 0.76 | 4.09 | 4.38E-05 | 0.0036 | 8.62 |
| LRATD2 | 214.62 | -0.60 | 0.20 | -3.03 | 0.00241 | 0.04587 | -1.52 |
| ASAP1 | 486.13 | 0.75 | 0.13 | 5.74 | 9.75E-09 | 7.21E-06 | 1.68 |
| TG | 15.73 | 1.52 | 0.44 | 3.41 | 0.00064 | 0.02032 | 2.86 |
| CCN4 | 362.57 | 1.84 | 0.34 | 5.41 | 6.43E-08 | 3.17E-05 | 3.57 |
| LY6E | 340.31 | 0.60 | 0.20 | 3.01 | 0.00265 | 0.04866 | 1.52 |
| EPPK1 | 14.35 | 1.39 | 0.42 | 3.34 | 0.00083 | 0.02413 | 2.62 |
| DOCK8 | 337.38 | 0.84 | 0.22 | 3.75 | 0.00018 | 0.00914 | 1.79 |
| JAK2 | 530.90 | 0.72 | 0.12 | 6.01 | 1.82E-09 | 1.84E-06 | 1.65 |
| MTCO1P11 | 8.52 | 1.70 | 0.46 | 3.69 | 0.00023 | 0.01049 | 3.25 |
| MTCO3P11 | 6.83 | 1.99 | 0.56 | 3.58 | 0.00034 | 0.01301 | 3.97 |
| CD72 | 28.19 | 1.29 | 0.30 | 4.31 | 1.66E-05 | 0.00192 | 2.45 |
| ALDH1B1 | 137.34 | 0.68 | 0.21 | 3.22 | 0.00128 | 0.03143 | 1.61 |
| PGM5P2 | 13.59 | -1.33 | 0.35 | -3.81 | 0.00014 | 0.0078 | -2.51 |
| FP325317.1 | 4.45 | -2.40 | 0.68 | -3.52 | 0.00044 | 0.01583 | -5.29 |
| FAM189A2 | 65.95 | -0.71 | 0.20 | -3.61 | 0.0003 | 0.01219 | -1.64 |
| ALDH1A1 | 432.09 | -1.19 | 0.29 | -4.10 | 4.09E-05 | 0.00344 | -2.28 |
| GCNT1 | 157.36 | 0.69 | 0.22 | 3.12 | 0.00178 | 0.03849 | 1.61 |
| PSAT1 | 44.14 | -0.97 | 0.29 | -3.36 | 0.00077 | 0.02304 | -1.96 |
| GKAP1 | 34.74 | -0.83 | 0.22 | -3.82 | 0.00014 | 0.00772 | -1.78 |
| SLC28A3 | 5.18 | 3.37 | 0.99 | 3.42 | 0.00063 | 0.02008 | 10.32 |
| SEMA4D | 114.51 | 0.99 | 0.31 | 3.20 | 0.00139 | 0.03308 | 1.99 |
| ROR2 | 53.41 | 1.23 | 0.33 | 3.73 | 0.00019 | 0.00944 | 2.34 |
| OGN | 592.76 | 1.48 | 0.21 | 6.94 | 3.98E-12 | 1.25E-08 | 2.79 |
| OMD | 807.47 | 0.84 | 0.25 | 3.40 | 0.00067 | 0.02104 | 1.78 |
| ASPN | 2,044.13 | 1.17 | 0.27 | 4.32 | 1.55E-05 | 0.00185 | 2.25 |
| FGD3 | 73.09 | 0.89 | 0.28 | 3.19 | 0.00141 | 0.03334 | 1.86 |
| FBP1 | 22.44 | 1.38 | 0.45 | 3.07 | 0.00212 | 0.04225 | 2.59 |
| TRIM14 | 104.11 | 0.80 | 0.24 | 3.35 | 0.0008 | 0.0237 | 1.74 |
| TBC1D2 | 81.84 | 0.88 | 0.20 | 4.39 | 1.14E-05 | 0.00147 | 1.85 |
| COL15A1 | 2,796.40 | 0.77 | 0.24 | 3.22 | 0.00128 | 0.03143 | 1.71 |
| EPB41L4B | 30.50 | -1.86 | 0.49 | -3.80 | 0.00014 | 0.00793 | -3.63 |
| SLC31A2 | 96.18 | 0.73 | 0.23 | 3.15 | 0.00164 | 0.0364 | 1.66 |
| TNC | 6,181.37 | 2.56 | 0.35 | 7.26 | 3.89E-13 | 3.74E-09 | 5.90 |
| NEK6 | 392.33 | 0.64 | 0.16 | 4.05 | 5.13E-05 | 0.00393 | 1.56 |
| ANGPTL2 | 693.38 | 1.06 | 0.31 | 3.45 | 0.00057 | 0.01868 | 2.08 |
| CERCAM | 553.09 | 0.66 | 0.17 | 3.97 | 7.10E-05 | 0.00496 | 1.58 |
| LINC01503 | 29.15 | 0.90 | 0.24 | 3.66 | 0.00025 | 0.01117 | 1.86 |
| LAMC3 | 372.23 | -1.10 | 0.33 | -3.33 | 0.00087 | 0.02484 | -2.15 |
| RAPGEF1 | 363.71 | 0.63 | 0.18 | 3.44 | 0.00058 | 0.01906 | 1.55 |
| AKR1C1 | 1,070.76 | -0.74 | 0.21 | -3.53 | 0.00042 | 0.01535 | -1.67 |
| CUBN | 166.48 | -0.64 | 0.16 | -3.96 | 7.55E-05 | 0.00519 | -1.56 |
| TMEM236 | 23.79 | 1.26 | 0.28 | 4.57 | 4.97E-06 | 0.00079 | 2.39 |
| KIAA1217 | 104.45 | 1.66 | 0.34 | 4.92 | 8.77E-07 | 0.00023 | 3.15 |
| PRTFDC1 | 258.47 | -0.64 | 0.21 | -3.04 | 0.00238 | 0.04571 | -1.56 |
| APBB1IP | 61.58 | 1.03 | 0.30 | 3.44 | 0.00058 | 0.01906 | 2.04 |
| BAMBI | 75.46 | -0.67 | 0.20 | -3.39 | 0.0007 | 0.02138 | -1.60 |
| LINC00841 | 4.94 | 2.33 | 0.72 | 3.23 | 0.00125 | 0.03101 | 5.03 |
| CXCL12 | 1,856.22 | 0.66 | 0.21 | 3.21 | 0.00131 | 0.03191 | 1.58 |
| DEPP1 | 2,187.25 | -0.85 | 0.24 | -3.57 | 0.00036 | 0.01353 | -1.80 |
| ALOX5 | 200.45 | 1.10 | 0.24 | 4.66 | 3.10E-06 | 0.00058 | 2.14 |
| WDFY4 | 197.17 | 0.82 | 0.26 | 3.13 | 0.00174 | 0.03788 | 1.77 |
| SLC16A9 | 74.08 | -1.13 | 0.33 | -3.44 | 0.00058 | 0.01891 | -2.19 |
| LINC01515 | 40.44 | -2.03 | 0.42 | -4.80 | 1.62E-06 | 0.00035 | -4.07 |
| TSPAN15 | 79.12 | 1.08 | 0.26 | 4.25 | 2.18E-05 | 0.00222 | 2.12 |
| NPFFR1 | 14.55 | -1.50 | 0.44 | -3.39 | 0.00069 | 0.0212 | -2.82 |
| PRF1 | 19.78 | 2.20 | 0.57 | 3.87 | 0.00011 | 0.00658 | 4.58 |
| MYOZ1 | 11.68 | 2.64 | 0.68 | 3.90 | 9.78E-05 | 0.00619 | 6.21 |
| PLAU | 332.80 | 1.29 | 0.31 | 4.23 | 2.34E-05 | 0.00229 | 2.45 |
| MAT1A | 11.01 | 4.95 | 1.28 | 3.88 | 0.00011 | 0.00648 | 30.88 |
| SLC16A12 | 104.22 | -1.11 | 0.32 | -3.49 | 0.00048 | 0.01679 | -2.15 |
| PIK3AP1 | 285.03 | 0.83 | 0.23 | 3.61 | 0.0003 | 0.01219 | 1.78 |
| CRTAC1 | 1,520.41 | 1.46 | 0.24 | 5.98 | 2.22E-09 | 2.13E-06 | 2.74 |
| SH3PXD2A | 719.44 | 0.67 | 0.17 | 3.98 | 6.93E-05 | 0.0049 | 1.60 |
| ADRA2A | 70.80 | 3.39 | 0.63 | 5.35 | 8.91E-08 | 3.81E-05 | 10.45 |
| PLEKHS1 | 20.06 | 5.88 | 0.93 | 6.29 | 3.19E-10 | 4.64E-07 | 58.70 |
| NANOS1 | 121.50 | -1.28 | 0.34 | -3.73 | 0.00019 | 0.0095 | -2.43 |
| HTRA1 | 1,684.03 | 0.85 | 0.18 | 4.68 | 2.91E-06 | 0.00055 | 1.81 |
| ADAM12 | 59.65 | 1.48 | 0.47 | 3.12 | 0.00179 | 0.03849 | 2.79 |
| PTPRE | 250.59 | 0.62 | 0.19 | 3.30 | 0.00096 | 0.02611 | 1.54 |
| ADAM8 | 60.41 | 1.67 | 0.49 | 3.45 | 0.00056 | 0.01866 | 3.19 |
| IFITM10 | 35.73 | 1.35 | 0.40 | 3.34 | 0.00084 | 0.02418 | 2.54 |
| AC068580.4 | 55.02 | 1.67 | 0.39 | 4.24 | 2.19E-05 | 0.00222 | 3.18 |
| LSP1 | 344.59 | 1.15 | 0.29 | 4.04 | 5.45E-05 | 0.00412 | 2.22 |
| KCNQ1 | 53.71 | 0.88 | 0.25 | 3.56 | 0.00037 | 0.014 | 1.85 |
| HBB | 905.12 | 2.39 | 0.77 | 3.12 | 0.00179 | 0.03849 | 5.25 |
| TPP1 | 368.66 | 0.84 | 0.23 | 3.64 | 0.00027 | 0.01158 | 1.79 |
| AMPD3 | 77.12 | 1.03 | 0.30 | 3.46 | 0.00053 | 0.01788 | 2.04 |
| PDE3B | 366.37 | -0.65 | 0.21 | -3.03 | 0.00241 | 0.04587 | -1.57 |
| INSC | 4.83 | 5.07 | 1.43 | 3.55 | 0.00038 | 0.01426 | 33.49 |
| ABCC8 | 5.63 | -3.71 | 0.90 | -4.14 | 3.51E-05 | 0.00307 | -13.10 |
| LDHA | 1,566.71 | 0.59 | 0.18 | 3.27 | 0.00106 | 0.02793 | 1.50 |
| CD44 | 1,867.04 | 0.67 | 0.19 | 3.61 | 0.00031 | 0.01232 | 1.59 |
| TP53I11 | 95.67 | 1.50 | 0.35 | 4.22 | 2.40E-05 | 0.00234 | 2.82 |
| SLC35C1 | 74.37 | 0.61 | 0.20 | 3.09 | 0.00201 | 0.04076 | 1.53 |
| SPI1 | 134.47 | 0.96 | 0.28 | 3.40 | 0.00066 | 0.02093 | 1.94 |
| MS4A2 | 19.34 | 1.94 | 0.61 | 3.19 | 0.00142 | 0.0334 | 3.85 |
| MS4A6A | 677.48 | 0.73 | 0.19 | 3.89 | 0.0001 | 0.0064 | 1.66 |
| CD5 | 15.03 | 2.19 | 0.62 | 3.52 | 0.00043 | 0.01562 | 4.57 |
| PLAAT4 | 126.86 | 0.63 | 0.19 | 3.27 | 0.00106 | 0.02793 | 1.55 |
| TM7SF2 | 42.40 | -1.62 | 0.41 | -3.95 | 7.96E-05 | 0.00535 | -3.07 |
| SYT12 | 205.96 | 1.79 | 0.39 | 4.66 | 3.23E-06 | 0.00058 | 3.47 |
| RHOD | 50.51 | 0.84 | 0.19 | 4.41 | 1.04E-05 | 0.0014 | 1.79 |
| P2RY6 | 58.58 | 1.13 | 0.30 | 3.79 | 0.00015 | 0.00815 | 2.19 |
| UCP2 | 72.19 | 1.08 | 0.28 | 3.79 | 0.00015 | 0.00815 | 2.11 |
| P4HA3 | 46.15 | 0.98 | 0.30 | 3.22 | 0.00128 | 0.03143 | 1.98 |
| CHRDL2 | 28.92 | 2.92 | 0.72 | 4.04 | 5.30E-05 | 0.00405 | 7.57 |
| SLCO2B1 | 1,294.51 | 0.84 | 0.25 | 3.34 | 0.00083 | 0.02405 | 1.79 |
| TENM4 | 118.55 | 1.19 | 0.34 | 3.51 | 0.00045 | 0.01598 | 2.28 |
| DLG2 | 184.44 | 1.10 | 0.27 | 4.07 | 4.72E-05 | 0.00372 | 2.14 |
| CTSC | 1,006.34 | 0.83 | 0.26 | 3.18 | 0.00146 | 0.03383 | 1.77 |
| CFAP300 | 34.53 | -0.77 | 0.20 | -3.81 | 0.00014 | 0.00779 | -1.71 |
| MMP7 | 10.39 | 4.20 | 1.21 | 3.47 | 0.00051 | 0.01734 | 18.43 |
| MMP1 | 7.81 | 5.05 | 1.16 | 4.35 | 1.37E-05 | 0.0017 | 33.12 |
| MMP12 | 18.29 | 4.29 | 1.03 | 4.18 | 2.92E-05 | 0.00273 | 19.54 |
| MMP13 | 35.80 | 6.84 | 1.53 | 4.48 | 7.54E-06 | 0.00112 | 114.48 |
| IL18 | 35.98 | 0.93 | 0.28 | 3.31 | 0.00095 | 0.02592 | 1.90 |
| IL10RA | 430.53 | 0.68 | 0.21 | 3.31 | 0.00093 | 0.02577 | 1.60 |
| CD3E | 17.56 | 2.01 | 0.59 | 3.39 | 0.00071 | 0.02153 | 4.02 |
| TLCD5 | 87.00 | -0.64 | 0.18 | -3.55 | 0.00038 | 0.01415 | -1.55 |
| ADAMTS15 | 130.17 | -0.82 | 0.24 | -3.45 | 0.00056 | 0.01864 | -1.77 |
| WNT5B | 37.62 | 1.36 | 0.37 | 3.64 | 0.00027 | 0.01158 | 2.57 |
| CACNA2D4 | 34.39 | 1.15 | 0.31 | 3.65 | 0.00026 | 0.01142 | 2.22 |
| LPAR5 | 37.70 | 0.89 | 0.27 | 3.27 | 0.00107 | 0.02802 | 1.85 |
| CD4 | 528.87 | 0.87 | 0.22 | 4.02 | 5.72E-05 | 0.00422 | 1.82 |
| PTPN6 | 149.49 | 0.89 | 0.29 | 3.06 | 0.00218 | 0.04297 | 1.86 |
| CD163L1 | 287.34 | 1.05 | 0.34 | 3.10 | 0.00194 | 0.04004 | 2.08 |
| C3AR1 | 265.14 | 0.83 | 0.24 | 3.43 | 0.0006 | 0.01945 | 1.78 |
| CLEC4D | 3.37 | 3.90 | 1.18 | 3.30 | 0.00097 | 0.02626 | 14.88 |
| MFAP5 | 104.51 | 1.95 | 0.62 | 3.14 | 0.00168 | 0.03693 | 3.88 |
| A2M | 5,817.86 | 0.83 | 0.19 | 4.41 | 1.06E-05 | 0.0014 | 1.78 |
| ARHGDIB | 766.19 | 0.59 | 0.15 | 3.93 | 8.54E-05 | 0.00567 | 1.51 |
| RERG | 311.76 | -0.70 | 0.17 | -3.98 | 6.87E-05 | 0.00487 | -1.62 |
| LMO3 | 216.61 | -0.90 | 0.29 | -3.11 | 0.0019 | 0.03982 | -1.87 |
| TSPAN11 | 121.41 | 1.48 | 0.41 | 3.59 | 0.00033 | 0.01267 | 2.80 |
| KIF21A | 226.15 | -0.70 | 0.19 | -3.70 | 0.00021 | 0.01002 | -1.62 |
| NELL2 | 32.95 | 3.13 | 0.86 | 3.64 | 0.00027 | 0.01156 | 8.77 |
| PCED1B-AS1 | 32.93 | 1.24 | 0.33 | 3.77 | 0.00016 | 0.00858 | 2.37 |
| VDR | 82.90 | 1.68 | 0.31 | 5.37 | 7.82E-08 | 3.58E-05 | 3.21 |
| COL2A1 | 13.38 | 2.87 | 0.80 | 3.60 | 0.00032 | 0.0124 | 7.33 |
| BIN2 | 77.25 | 1.15 | 0.27 | 4.24 | 2.27E-05 | 0.00225 | 2.22 |
| GALNT6 | 46.21 | 1.15 | 0.30 | 3.79 | 0.00015 | 0.00815 | 2.22 |
| ITGB7 | 29.54 | 1.29 | 0.36 | 3.64 | 0.00027 | 0.01158 | 2.45 |
| ESPL1 | 11.40 | 1.22 | 0.41 | 3.00 | 0.00269 | 0.04918 | 2.33 |
| SP7 | 8.98 | 6.16 | 1.27 | 4.86 | 1.15E-06 | 0.00028 | 71.32 |
| NCKAP1L | 511.65 | 0.72 | 0.20 | 3.65 | 0.00026 | 0.01142 | 1.64 |
| LRP1 | 14,866.26 | 0.65 | 0.18 | 3.51 | 0.00045 | 0.01607 | 1.57 |
| ARHGAP9 | 76.02 | 1.04 | 0.29 | 3.55 | 0.00038 | 0.01415 | 2.06 |
| AGAP2 | 31.84 | 1.18 | 0.34 | 3.49 | 0.00048 | 0.01679 | 2.27 |
| CYP27B1 | 3.36 | 3.44 | 0.90 | 3.82 | 0.00013 | 0.00766 | 10.86 |
| AVIL | 96.34 | 1.14 | 0.24 | 4.76 | 1.95E-06 | 0.00042 | 2.21 |
| LYZ | 486.43 | 1.50 | 0.44 | 3.39 | 0.00071 | 0.02156 | 2.83 |
| TSPAN8 | 154.50 | -1.10 | 0.34 | -3.24 | 0.0012 | 0.03009 | -2.14 |
| TRHDE | 155.63 | -1.23 | 0.25 | -4.83 | 1.40E-06 | 0.00031 | -2.34 |
| TRHDE-AS1 | 89.98 | -1.44 | 0.39 | -3.69 | 0.00023 | 0.01049 | -2.72 |
| CAPS2 | 101.30 | -0.70 | 0.23 | -3.11 | 0.00186 | 0.03928 | -1.63 |
| PTPRQ | 14.52 | -1.55 | 0.48 | -3.22 | 0.00128 | 0.03143 | -2.94 |
| LIN7A | 155.19 | -0.89 | 0.25 | -3.60 | 0.00031 | 0.01236 | -1.85 |
| MGAT4C | 10.19 | 2.00 | 0.64 | 3.11 | 0.0019 | 0.03982 | 4.00 |
| LUM | 12,067.03 | 1.11 | 0.28 | 3.89 | 9.94E-05 | 0.00625 | 2.16 |
| PLXNC1 | 250.61 | 0.74 | 0.20 | 3.70 | 0.00021 | 0.01002 | 1.67 |
| USP44 | 10.78 | -1.15 | 0.37 | -3.15 | 0.00163 | 0.03628 | -2.23 |
| BTBD11 | 31.78 | 1.06 | 0.28 | 3.83 | 0.00013 | 0.00748 | 2.08 |
| WSCD2 | 38.45 | 1.71 | 0.38 | 4.49 | 7.23E-06 | 0.00109 | 3.28 |
| CMKLR1 | 722.13 | 1.01 | 0.18 | 5.67 | 1.42E-08 | 9.40E-06 | 2.01 |
| TMEM119 | 177.38 | 1.20 | 0.28 | 4.25 | 2.09E-05 | 0.00219 | 2.29 |
| SELPLG | 138.72 | 0.95 | 0.26 | 3.64 | 0.00028 | 0.01159 | 1.93 |
| TRPV4 | 26.55 | 1.39 | 0.29 | 4.75 | 1.99E-06 | 0.00042 | 2.62 |
| SH2B3 | 441.40 | 0.62 | 0.17 | 3.64 | 0.00027 | 0.01156 | 1.54 |
| ALDH2 | 460.73 | -0.99 | 0.24 | -4.08 | 4.56E-05 | 0.00363 | -1.98 |
| OAS3 | 364.87 | 0.73 | 0.23 | 3.20 | 0.00138 | 0.03284 | 1.66 |
| OAS2 | 313.51 | 0.73 | 0.21 | 3.50 | 0.00047 | 0.01645 | 1.65 |
| SPRING1 | 166.95 | 0.63 | 0.16 | 4.03 | 5.49E-05 | 0.00413 | 1.55 |
| BICDL1 | 7.08 | 3.20 | 0.76 | 4.19 | 2.84E-05 | 0.00268 | 9.17 |
| OASL | 17.73 | 1.61 | 0.45 | 3.56 | 0.00038 | 0.01406 | 3.05 |
| RHOF | 35.30 | 0.84 | 0.26 | 3.29 | 0.00102 | 0.02716 | 1.79 |
| LINC00944 | 4.48 | 2.24 | 0.70 | 3.18 | 0.00148 | 0.03393 | 4.72 |
| TMEM132C | 85.71 | -1.29 | 0.41 | -3.19 | 0.00141 | 0.03327 | -2.45 |
| FZD10-AS1 | 107.19 | -0.92 | 0.25 | -3.62 | 0.0003 | 0.01217 | -1.89 |
| GJA3 | 3.02 | 3.51 | 1.05 | 3.34 | 0.00082 | 0.02401 | 11.38 |
| TNFRSF19 | 446.74 | -0.88 | 0.23 | -3.88 | 0.0001 | 0.00645 | -1.84 |
| BRCA2 | 78.66 | 0.65 | 0.21 | 3.07 | 0.00217 | 0.04288 | 1.57 |
| RGCC | 86.43 | 1.00 | 0.27 | 3.64 | 0.00027 | 0.01156 | 2.00 |
| TNFSF11 | 11.01 | 3.97 | 0.77 | 5.14 | 2.74E-07 | 8.95E-05 | 15.69 |
| EPSTI1 | 96.38 | 1.04 | 0.27 | 3.91 | 9.11E-05 | 0.00586 | 2.06 |
| LCP1 | 831.23 | 0.89 | 0.28 | 3.12 | 0.00183 | 0.03876 | 1.85 |
| CYSLTR2 | 240.65 | -0.68 | 0.21 | -3.20 | 0.0014 | 0.03313 | -1.60 |
| ARL11 | 19.18 | 1.07 | 0.30 | 3.58 | 0.00034 | 0.01299 | 2.10 |
| SLAIN1 | 52.69 | -0.92 | 0.21 | -4.32 | 1.58E-05 | 0.00187 | -1.90 |
| SLITRK5 | 30.10 | -1.78 | 0.39 | -4.63 | 3.71E-06 | 0.00065 | -3.44 |
| F10 | 144.47 | -0.75 | 0.23 | -3.27 | 0.00108 | 0.02825 | -1.68 |
| TMEM255B | 28.03 | 0.94 | 0.27 | 3.48 | 0.0005 | 0.01705 | 1.91 |
| LINC00565 | 8.46 | 1.38 | 0.43 | 3.22 | 0.00127 | 0.03141 | 2.60 |
| NDRG2 | 612.51 | -0.95 | 0.24 | -4.03 | 5.60E-05 | 0.00417 | -1.93 |
| SLC7A8 | 100.07 | 0.90 | 0.24 | 3.81 | 0.00014 | 0.00781 | 1.86 |
| RIPK3 | 38.89 | 0.89 | 0.26 | 3.47 | 0.00052 | 0.01753 | 1.85 |
| CMA1 | 7.89 | 4.30 | 1.04 | 4.12 | 3.87E-05 | 0.00329 | 19.63 |
| CTSG | 20.28 | 2.98 | 0.63 | 4.75 | 2.08E-06 | 0.00043 | 7.87 |
| GZMH | 5.92 | 1.86 | 0.62 | 3.02 | 0.00254 | 0.04756 | 3.64 |
| AL121821.2 | 17.65 | -1.26 | 0.30 | -4.16 | 3.19E-05 | 0.00287 | -2.39 |
| LRFN5 | 48.57 | -1.35 | 0.29 | -4.57 | 4.84E-06 | 0.00078 | -2.55 |
| RN7SL1 | 21,965.85 | 0.97 | 0.32 | 3.05 | 0.0023 | 0.04451 | 1.96 |
| GCH1 | 36.64 | 0.95 | 0.27 | 3.44 | 0.00057 | 0.01879 | 1.93 |
| HIF1A | 979.01 | 0.82 | 0.26 | 3.09 | 0.00197 | 0.0403 | 1.76 |
| SUSD6 | 373.81 | 0.70 | 0.19 | 3.65 | 0.00026 | 0.01144 | 1.62 |
| SMOC1 | 182.92 | 1.23 | 0.37 | 3.35 | 0.00082 | 0.02394 | 2.35 |
| SLC8A3 | 4.06 | 3.28 | 1.00 | 3.28 | 0.00104 | 0.02761 | 9.73 |
| NPC2 | 937.43 | 0.70 | 0.18 | 3.93 | 8.49E-05 | 0.00565 | 1.63 |
| PGF | 117.81 | 1.26 | 0.35 | 3.64 | 0.00028 | 0.01159 | 2.40 |
| BATF | 19.52 | 1.07 | 0.34 | 3.10 | 0.00196 | 0.04015 | 2.10 |
| FLVCR2 | 47.88 | 0.81 | 0.26 | 3.15 | 0.00162 | 0.03624 | 1.75 |
| LINC02289 | 7.10 | -1.70 | 0.47 | -3.63 | 0.00028 | 0.01166 | -3.25 |
| GPR65 | 52.27 | 0.82 | 0.27 | 3.07 | 0.00212 | 0.04227 | 1.76 |
| GPR68 | 56.18 | 2.66 | 0.47 | 5.71 | 1.11E-08 | 7.88E-06 | 6.34 |
| AL135818.1 | 3.43 | 3.03 | 0.98 | 3.09 | 0.00197 | 0.0403 | 8.18 |
| TC2N | 364.68 | -0.82 | 0.20 | -4.08 | 4.52E-05 | 0.00363 | -1.77 |
| PRIMA1 | 40.15 | -1.00 | 0.33 | -3.05 | 0.00228 | 0.04433 | -2.00 |
| CLMN | 148.40 | 0.72 | 0.20 | 3.55 | 0.00038 | 0.01415 | 1.65 |
| BCL11B | 21.11 | 2.11 | 0.58 | 3.66 | 0.00025 | 0.01128 | 4.31 |
| AHNAK2 | 1,541.78 | 0.74 | 0.20 | 3.72 | 0.0002 | 0.00982 | 1.67 |
| IGHG4 | 483.40 | 8.92 | 1.84 | 4.85 | 1.23E-06 | 0.00029 | 483.02 |
| IGHG2 | 2,277.83 | 4.22 | 1.29 | 3.27 | 0.00106 | 0.02802 | 18.64 |
| IGHA1 | 1,032.46 | 3.81 | 1.10 | 3.46 | 0.00053 | 0.01788 | 14.07 |
| IGHG1 | 3,712.26 | 3.72 | 1.18 | 3.14 | 0.0017 | 0.0373 | 13.13 |
| IGHV1-2 | 16.79 | 5.23 | 1.56 | 3.36 | 0.00079 | 0.02342 | 37.43 |
| IGHV4-34 | 18.02 | 6.23 | 1.79 | 3.48 | 0.00049 | 0.01702 | 75.00 |
| FAM189A1 | 38.45 | -0.98 | 0.26 | -3.74 | 0.00018 | 0.00914 | -1.98 |
| GREM1 | 38.20 | 3.45 | 1.03 | 3.33 | 0.00085 | 0.02456 | 10.92 |
| MEIS2 | 482.60 | -0.67 | 0.17 | -3.86 | 0.00011 | 0.00683 | -1.60 |
| LINC02345 | 6.48 | 2.68 | 0.76 | 3.52 | 0.00044 | 0.01574 | 6.40 |
| RASGRP1 | 30.80 | 1.91 | 0.47 | 4.08 | 4.42E-05 | 0.00362 | 3.76 |
| PLCB2 | 165.19 | 0.87 | 0.26 | 3.32 | 0.00089 | 0.02521 | 1.83 |
| SEMA6D | 167.53 | -0.86 | 0.15 | -5.85 | 5.06E-09 | 4.24E-06 | -1.81 |
| FGF7 | 766.17 | 1.54 | 0.31 | 4.96 | 6.91E-07 | 0.00019 | 2.92 |
| HDC | 18.40 | 2.70 | 0.60 | 4.47 | 7.68E-06 | 0.00113 | 6.51 |
| CYP19A1 | 10.97 | 1.37 | 0.39 | 3.50 | 0.00047 | 0.01646 | 2.59 |
| TEX9 | 99.16 | 1.02 | 0.25 | 4.16 | 3.13E-05 | 0.00284 | 2.03 |
| CA12 | 84.21 | 2.28 | 0.41 | 5.50 | 3.87E-08 | 2.25E-05 | 4.86 |
| CORO2B | 120.39 | -0.70 | 0.20 | -3.45 | 0.00056 | 0.01856 | -1.62 |
| THSD4 | 278.53 | -0.97 | 0.27 | -3.66 | 0.00026 | 0.01136 | -1.96 |
| STRA6 | 77.14 | -0.98 | 0.29 | -3.38 | 0.00072 | 0.02183 | -1.98 |
| SEMA7A | 41.19 | 1.39 | 0.43 | 3.23 | 0.00123 | 0.03058 | 2.63 |
| CRABP1 | 7.29 | 2.31 | 0.68 | 3.40 | 0.00069 | 0.0212 | 4.95 |
| CTSH | 333.66 | 0.90 | 0.25 | 3.66 | 0.00025 | 0.01132 | 1.87 |
| CEMIP | 497.06 | 2.23 | 0.47 | 4.70 | 2.57E-06 | 0.0005 | 4.69 |
| TM6SF1 | 76.22 | 0.64 | 0.21 | 3.02 | 0.00254 | 0.04756 | 1.56 |
| LINC00933 | 8.55 | -1.09 | 0.35 | -3.12 | 0.00183 | 0.03876 | -2.12 |
| ALPK3 | 169.17 | 0.70 | 0.17 | 4.01 | 6.16E-05 | 0.00447 | 1.63 |
| ACAN | 596.23 | 2.86 | 0.54 | 5.29 | 1.23E-07 | 4.95E-05 | 7.28 |
| HAPLN3 | 123.93 | 1.49 | 0.32 | 4.66 | 3.20E-06 | 0.00058 | 2.80 |
| ANPEP | 265.77 | 1.58 | 0.28 | 5.65 | 1.64E-08 | 1.05E-05 | 2.99 |
| SLCO3A1 | 186.16 | 0.60 | 0.13 | 4.64 | 3.46E-06 | 0.00062 | 1.52 |
| MCTP2 | 56.13 | 1.30 | 0.27 | 4.88 | 1.04E-06 | 0.00026 | 2.46 |
| HBA2 | 212.30 | 2.37 | 0.73 | 3.23 | 0.00122 | 0.03049 | 5.16 |
| HBA1 | 48.97 | 2.44 | 0.73 | 3.33 | 0.00086 | 0.0247 | 5.44 |
| TPSB2 | 31.41 | 3.57 | 0.74 | 4.81 | 1.52E-06 | 0.00034 | 11.88 |
| TPSAB1 | 31.42 | 2.56 | 0.60 | 4.25 | 2.18E-05 | 0.00222 | 5.90 |
| TPSD1 | 3.52 | 3.60 | 1.17 | 3.07 | 0.00211 | 0.0422 | 12.14 |
| MEFV | 18.53 | 1.56 | 0.47 | 3.32 | 0.0009 | 0.0253 | 2.94 |
| NLRC3 | 36.45 | 1.50 | 0.48 | 3.10 | 0.00194 | 0.04004 | 2.83 |
| AC099489.1 | 23.91 | 0.93 | 0.28 | 3.34 | 0.00084 | 0.02421 | 1.91 |
| XYLT1 | 352.73 | 0.78 | 0.15 | 5.20 | 1.99E-07 | 7.36E-05 | 1.72 |
| IL21R | 35.49 | 2.46 | 0.57 | 4.30 | 1.68E-05 | 0.00193 | 5.50 |
| CORO1A | 167.04 | 0.92 | 0.30 | 3.09 | 0.00201 | 0.04065 | 1.89 |
| ITGAM | 328.85 | 0.70 | 0.18 | 3.97 | 7.31E-05 | 0.00508 | 1.63 |
| ITGAX | 128.65 | 1.89 | 0.61 | 3.12 | 0.00181 | 0.03865 | 3.71 |
| MYLK3 | 11.92 | -1.84 | 0.41 | -4.47 | 7.92E-06 | 0.00115 | -3.59 |
| NETO2 | 58.19 | -0.91 | 0.29 | -3.11 | 0.00188 | 0.03961 | -1.88 |
| SNX20 | 39.40 | 1.29 | 0.31 | 4.21 | 2.51E-05 | 0.00241 | 2.44 |
| NOD2 | 32.62 | 0.92 | 0.30 | 3.09 | 0.00198 | 0.04032 | 1.89 |
| MMP2 | 3,712.52 | 0.79 | 0.21 | 3.78 | 0.00016 | 0.00832 | 1.73 |
| LPCAT2 | 206.34 | 0.66 | 0.16 | 4.13 | 3.69E-05 | 0.00319 | 1.58 |
| NUDT7 | 26.40 | -0.82 | 0.25 | -3.32 | 0.00091 | 0.02542 | -1.77 |
| CLEC3A | 5.66 | 4.62 | 1.21 | 3.83 | 0.00013 | 0.00748 | 24.57 |
| PKD1L2 | 5.57 | -1.71 | 0.53 | -3.26 | 0.0011 | 0.02855 | -3.28 |
| PLCG2 | 113.38 | 0.95 | 0.31 | 3.09 | 0.00197 | 0.0403 | 1.93 |
| WFDC1 | 54.45 | 1.14 | 0.34 | 3.32 | 0.00089 | 0.02512 | 2.21 |
| IRF8 | 97.47 | 0.61 | 0.20 | 3.01 | 0.00264 | 0.04866 | 1.53 |
| CYBA | 425.28 | 0.67 | 0.20 | 3.32 | 0.00089 | 0.02512 | 1.59 |
| CPNE7 | 4.69 | 3.19 | 1.05 | 3.03 | 0.00241 | 0.04587 | 9.15 |
| CXCL16 | 215.73 | 1.10 | 0.29 | 3.83 | 0.00013 | 0.00754 | 2.15 |
| VMO1 | 35.58 | 1.37 | 0.40 | 3.44 | 0.00059 | 0.01925 | 2.59 |
| ACAP1 | 41.31 | 1.51 | 0.47 | 3.19 | 0.00141 | 0.03327 | 2.86 |
| PIK3R5 | 139.85 | 0.76 | 0.24 | 3.21 | 0.00132 | 0.03203 | 1.70 |
| HS3ST3B1 | 20.41 | 1.46 | 0.47 | 3.10 | 0.00195 | 0.04015 | 2.74 |
| MEIS3P1 | 65.83 | -0.66 | 0.18 | -3.73 | 0.00019 | 0.00952 | -1.58 |
| LGALS9 | 95.13 | 0.83 | 0.25 | 3.40 | 0.00067 | 0.02104 | 1.78 |
| EVI2B | 42.54 | 1.08 | 0.31 | 3.49 | 0.00049 | 0.01699 | 2.11 |
| MYO1D | 2,236.15 | 0.99 | 0.26 | 3.82 | 0.00013 | 0.00766 | 1.99 |
| CCL11 | 4.86 | 3.20 | 0.99 | 3.25 | 0.00117 | 0.02988 | 9.21 |
| CCL8 | 32.51 | 2.77 | 0.44 | 6.24 | 4.38E-10 | 5.62E-07 | 6.81 |
| CCL5 | 69.25 | 2.13 | 0.53 | 4.00 | 6.40E-05 | 0.00461 | 4.39 |
| CCL3 | 28.08 | 1.97 | 0.55 | 3.60 | 0.00032 | 0.0124 | 3.93 |
| CCL4 | 17.72 | 1.76 | 0.52 | 3.36 | 0.00079 | 0.02342 | 3.38 |
| AC243829.1 | 6.96 | 1.96 | 0.64 | 3.08 | 0.00208 | 0.04162 | 3.90 |
| CCL4L2 | 10.22 | 2.64 | 0.70 | 3.75 | 0.00018 | 0.00901 | 6.24 |
| STAC2 | 10.56 | 1.80 | 0.53 | 3.39 | 0.00069 | 0.0212 | 3.49 |
| IKZF3 | 68.38 | 2.05 | 0.64 | 3.19 | 0.00145 | 0.0338 | 4.14 |
| KRT16 | 12.30 | 3.10 | 0.63 | 4.90 | 9.57E-07 | 0.00024 | 8.58 |
| KRT17 | 58.25 | 1.56 | 0.38 | 4.06 | 5.00E-05 | 0.00389 | 2.94 |
| AOC2 | 14.06 | 1.02 | 0.34 | 2.99 | 0.00278 | 0.04996 | 2.03 |
| RND2 | 32.55 | -0.85 | 0.26 | -3.24 | 0.0012 | 0.03009 | -1.80 |
| FMNL1 | 131.66 | 1.07 | 0.32 | 3.31 | 0.00095 | 0.02592 | 2.10 |
| MAPT | 108.66 | -1.35 | 0.28 | -4.90 | 9.48E-07 | 0.00024 | -2.55 |
| RPRML | 3.28 | 3.31 | 1.05 | 3.15 | 0.00162 | 0.03619 | 9.95 |
| TBX21 | 3.48 | 2.73 | 0.87 | 3.12 | 0.00182 | 0.03876 | 6.61 |
| SKAP1 | 7.73 | 2.52 | 0.65 | 3.87 | 0.00011 | 0.00666 | 5.74 |
| HOXB6 | 12.60 | 1.59 | 0.37 | 4.30 | 1.69E-05 | 0.00193 | 3.01 |
| DLX3 | 5.63 | 2.33 | 0.60 | 3.92 | 9.01E-05 | 0.00586 | 5.03 |
| ABCC3 | 280.62 | 0.87 | 0.26 | 3.37 | 0.00076 | 0.02282 | 1.82 |
| TMEM100 | 139.14 | -1.10 | 0.33 | -3.30 | 0.00098 | 0.02653 | -2.15 |
| ACE | 19.88 | 1.86 | 0.43 | 4.38 | 1.20E-05 | 0.00153 | 3.64 |
| LIMD2 | 103.33 | 0.80 | 0.26 | 3.08 | 0.0021 | 0.04199 | 1.74 |
| MILR1 | 95.13 | 0.71 | 0.22 | 3.19 | 0.00142 | 0.03337 | 1.64 |
| RGS9 | 34.34 | -0.81 | 0.25 | -3.21 | 0.00131 | 0.03182 | -1.75 |
| AXIN2 | 145.66 | -1.11 | 0.18 | -6.28 | 3.38E-10 | 4.64E-07 | -2.16 |
| SLC16A6 | 8.82 | 2.03 | 0.52 | 3.91 | 9.08E-05 | 0.00586 | 4.07 |
| FAM20A | 190.76 | 1.63 | 0.28 | 5.74 | 9.57E-09 | 7.21E-06 | 3.10 |
| ABCA8 | 2,669.63 | -1.02 | 0.31 | -3.26 | 0.0011 | 0.02847 | -2.02 |
| ABCA9 | 955.99 | -0.85 | 0.17 | -4.92 | 8.82E-07 | 0.00023 | -1.80 |
| ABCA6 | 1,923.80 | -0.73 | 0.20 | -3.65 | 0.00026 | 0.01142 | -1.66 |
| ABCA5 | 244.15 | -0.63 | 0.20 | -3.20 | 0.00138 | 0.03284 | -1.55 |
| MAP2K6 | 147.62 | -1.03 | 0.21 | -4.84 | 1.28E-06 | 0.0003 | -2.04 |
| CD300C | 23.65 | 1.32 | 0.37 | 3.61 | 0.0003 | 0.01219 | 2.50 |
| RHBDF2 | 159.18 | 0.91 | 0.26 | 3.54 | 0.00039 | 0.01453 | 1.87 |
| PRCD | 77.60 | -1.07 | 0.36 | -3.01 | 0.00262 | 0.04848 | -2.10 |
| SYNGR2 | 202.57 | 0.81 | 0.19 | 4.24 | 2.27E-05 | 0.00225 | 1.75 |
| PYCR1 | 122.05 | 1.07 | 0.27 | 3.95 | 7.73E-05 | 0.00525 | 2.10 |
| SLC16A3 | 155.03 | 1.30 | 0.38 | 3.40 | 0.00067 | 0.02104 | 2.46 |
| CD7 | 7.00 | 2.51 | 0.69 | 3.63 | 0.00028 | 0.01164 | 5.70 |
| SECTM1 | 44.10 | 1.25 | 0.34 | 3.64 | 0.00027 | 0.01158 | 2.38 |
| METRNL | 225.75 | 0.63 | 0.20 | 3.14 | 0.00167 | 0.03689 | 1.55 |
| ADCYAP1 | 6.41 | 4.44 | 1.22 | 3.64 | 0.00027 | 0.01156 | 21.77 |
| GAPLINC | 8.90 | 1.51 | 0.41 | 3.69 | 0.00023 | 0.01049 | 2.85 |
| APCDD1 | 1,368.49 | -1.26 | 0.25 | -5.11 | 3.25E-07 | 9.94E-05 | -2.39 |
| GREB1L | 4.14 | -2.41 | 0.58 | -4.17 | 3.03E-05 | 0.00277 | -5.32 |
| GATA6-AS1 | 33.24 | -0.82 | 0.24 | -3.36 | 0.00077 | 0.02296 | -1.77 |
| ASXL3 | 72.72 | -1.14 | 0.28 | -4.09 | 4.33E-05 | 0.00357 | -2.20 |
| FHOD3 | 65.31 | 0.97 | 0.32 | 3.07 | 0.00217 | 0.04288 | 1.95 |
| SIGLEC15 | 6.51 | 2.40 | 0.79 | 3.05 | 0.00227 | 0.04418 | 5.29 |
| MYO5B | 22.54 | 1.18 | 0.33 | 3.58 | 0.00035 | 0.01327 | 2.27 |
| RAB27B | 13.73 | 2.30 | 0.54 | 4.30 | 1.73E-05 | 0.00195 | 4.93 |
| DOK6 | 136.54 | -0.97 | 0.23 | -4.26 | 2.04E-05 | 0.00216 | -1.96 |
| CD226 | 36.17 | 1.13 | 0.32 | 3.48 | 0.0005 | 0.01719 | 2.19 |
| AC016588.2 | 4.08 | -2.08 | 0.69 | -3.02 | 0.00251 | 0.0472 | -4.24 |
| ARID3A | 45.25 | 0.73 | 0.21 | 3.38 | 0.00071 | 0.02163 | 1.65 |
| ARHGAP45 | 205.95 | 0.83 | 0.27 | 3.05 | 0.0023 | 0.04451 | 1.78 |
| DIRAS1 | 22.44 | 1.43 | 0.36 | 3.96 | 7.61E-05 | 0.0052 | 2.69 |
| MFSD12 | 200.99 | 0.78 | 0.18 | 4.43 | 9.40E-06 | 0.00132 | 1.71 |
| MATK | 20.49 | 2.25 | 0.56 | 4.05 | 5.04E-05 | 0.00389 | 4.77 |
| SEMA6B | 30.10 | 2.25 | 0.67 | 3.33 | 0.00086 | 0.02457 | 4.74 |
| VAV1 | 102.46 | 0.72 | 0.22 | 3.32 | 0.0009 | 0.02528 | 1.65 |
| ADGRE4P | 6.40 | 3.12 | 0.90 | 3.45 | 0.00055 | 0.01839 | 8.69 |
| STXBP2 | 27.28 | 1.21 | 0.35 | 3.42 | 0.00062 | 0.01994 | 2.31 |
| CD209 | 126.86 | 1.77 | 0.48 | 3.72 | 0.0002 | 0.00961 | 3.41 |
| MYO1F | 181.46 | 1.09 | 0.29 | 3.76 | 0.00017 | 0.00885 | 2.13 |
| COL5A3 | 260.65 | -0.71 | 0.21 | -3.48 | 0.0005 | 0.01702 | -1.64 |
| ACP5 | 198.33 | 1.68 | 0.46 | 3.67 | 0.00025 | 0.01107 | 3.20 |
| ADGRE2 | 62.39 | 2.17 | 0.60 | 3.60 | 0.00032 | 0.01251 | 4.52 |
| RASAL3 | 40.15 | 1.50 | 0.39 | 3.81 | 0.00014 | 0.0079 | 2.83 |
| CYP4F12 | 55.29 | -1.09 | 0.30 | -3.63 | 0.00028 | 0.01169 | -2.13 |
| HSH2D | 9.11 | 2.42 | 0.80 | 3.03 | 0.00246 | 0.04663 | 5.36 |
| TMEM38A | 29.72 | -1.04 | 0.29 | -3.57 | 0.00036 | 0.01368 | -2.06 |
| NWD1 | 59.39 | -1.14 | 0.36 | -3.20 | 0.00136 | 0.03256 | -2.21 |
| JAK3 | 170.42 | 1.21 | 0.32 | 3.72 | 0.0002 | 0.00961 | 2.31 |
| IL12RB1 | 22.13 | 1.13 | 0.34 | 3.33 | 0.00088 | 0.02511 | 2.19 |
| CRLF1 | 370.18 | 1.52 | 0.28 | 5.42 | 6.06E-08 | 3.15E-05 | 2.87 |
| COMP | 2,826.05 | 1.84 | 0.36 | 5.10 | 3.34E-07 | 0.0001 | 3.57 |
| CILP2 | 77.81 | 2.27 | 0.47 | 4.86 | 1.17E-06 | 0.00028 | 4.84 |
| GMIP | 84.74 | 0.76 | 0.24 | 3.09 | 0.00197 | 0.0403 | 1.69 |
| HCST | 53.16 | 1.11 | 0.29 | 3.77 | 0.00016 | 0.00857 | 2.15 |
| TYROBP | 343.38 | 1.10 | 0.27 | 4.04 | 5.46E-05 | 0.00412 | 2.15 |
| SPINT2 | 65.05 | 0.69 | 0.19 | 3.59 | 0.00033 | 0.01292 | 1.62 |
| FCGBP | 1,969.37 | 1.79 | 0.47 | 3.83 | 0.00013 | 0.00754 | 3.47 |
| POU2F2 | 42.55 | 1.13 | 0.33 | 3.38 | 0.00073 | 0.02208 | 2.18 |
| ETHE1 | 61.98 | 0.74 | 0.20 | 3.61 | 0.00031 | 0.01236 | 1.67 |
| KCNN4 | 68.25 | 2.05 | 0.36 | 5.69 | 1.29E-08 | 8.89E-06 | 4.15 |
| C5AR2 | 68.52 | 0.85 | 0.22 | 3.94 | 8.17E-05 | 0.00548 | 1.80 |
| CD37 | 52.88 | 1.12 | 0.33 | 3.39 | 0.00071 | 0.02156 | 2.18 |
| IL4I1 | 8.78 | 2.52 | 0.66 | 3.84 | 0.00012 | 0.00733 | 5.72 |
| CD33 | 69.22 | 0.83 | 0.25 | 3.31 | 0.00093 | 0.02569 | 1.78 |
| NKG7 | 10.55 | 1.72 | 0.54 | 3.21 | 0.00132 | 0.03198 | 3.30 |
| LILRB3 | 42.98 | 1.25 | 0.40 | 3.10 | 0.00194 | 0.04004 | 2.38 |
| LAIR1 | 309.37 | 1.08 | 0.25 | 4.31 | 1.64E-05 | 0.00191 | 2.11 |
| LILRB1 | 51.80 | 1.40 | 0.43 | 3.29 | 0.001 | 0.02699 | 2.65 |
| LILRB4 | 124.37 | 1.24 | 0.34 | 3.60 | 0.00032 | 0.01245 | 2.35 |
| IL11 | 9.69 | 3.10 | 0.85 | 3.65 | 0.00026 | 0.01138 | 8.55 |
| SLC4A11 | 5.40 | 2.31 | 0.63 | 3.64 | 0.00028 | 0.01159 | 4.95 |
| SIGLEC1 | 470.81 | 1.07 | 0.31 | 3.41 | 0.00066 | 0.02083 | 2.10 |
| ISM1 | 36.13 | 2.37 | 0.50 | 4.73 | 2.24E-06 | 0.00046 | 5.17 |
| CD93 | 1,269.85 | 1.43 | 0.29 | 5.01 | 5.54E-07 | 0.00016 | 2.70 |
| SYNDIG1 | 46.38 | 1.39 | 0.43 | 3.24 | 0.00119 | 0.03003 | 2.62 |
| CST7 | 11.70 | 2.24 | 0.61 | 3.65 | 0.00026 | 0.01137 | 4.73 |
| MAFB | 294.99 | 0.77 | 0.25 | 3.12 | 0.0018 | 0.03849 | 1.71 |
| EMILIN3 | 124.39 | -1.16 | 0.32 | -3.68 | 0.00023 | 0.01069 | -2.24 |
| RIMS4 | 101.17 | -0.94 | 0.25 | -3.71 | 0.00021 | 0.00993 | -1.92 |
| SLPI | 96.09 | 2.91 | 0.42 | 6.92 | 4.53E-12 | 1.25E-08 | 7.53 |
| MATN4 | 23.11 | 1.84 | 0.59 | 3.14 | 0.0017 | 0.03724 | 3.58 |
| NEURL2 | 30.83 | 1.13 | 0.37 | 3.02 | 0.0025 | 0.04716 | 2.19 |
| AL008726.1 | 25.38 | 1.74 | 0.58 | 2.99 | 0.00275 | 0.04983 | 3.34 |
| PLTP | 2,444.23 | 1.21 | 0.30 | 3.98 | 6.79E-05 | 0.00486 | 2.31 |
| MMP9 | 229.46 | 4.72 | 0.98 | 4.84 | 1.31E-06 | 0.0003 | 26.32 |
| KCNG1 | 16.08 | -0.83 | 0.26 | -3.17 | 0.00151 | 0.03426 | -1.78 |
| CBLN4 | 6.64 | 4.52 | 1.23 | 3.68 | 0.00023 | 0.01069 | 22.99 |
| PCK1 | 5.39 | -3.11 | 0.62 | -5.04 | 4.78E-07 | 0.00014 | -8.65 |
| APCDD1L | 33.18 | 1.77 | 0.56 | 3.18 | 0.00146 | 0.03383 | 3.40 |
| CTSZ | 1493.52 | 0.70 | 0.18 | 3.86 | 0.00012 | 0.00694 | 1.62 |
| ZNF831 | 18.38 | 2.35 | 0.59 | 3.97 | 7.11E-05 | 0.00496 | 5.11 |
| RGS19 | 70.19 | 0.84 | 0.23 | 3.58 | 0.00035 | 0.01333 | 1.79 |
| CHODL | 8.28 | -1.67 | 0.46 | -3.64 | 0.00027 | 0.01156 | -3.17 |
| RUNX1 | 768.24 | 1.04 | 0.19 | 5.34 | 9.37E-08 | 3.92E-05 | 2.06 |
| KCNJ6 | 13.49 | 6.02 | 1.15 | 5.22 | 1.78E-07 | 6.85E-05 | 64.72 |
| KCNJ15 | 23.43 | 1.93 | 0.47 | 4.09 | 4.27E-05 | 0.00354 | 3.82 |
| SPATA20P1 | 4.02 | 5.17 | 1.17 | 4.40 | 1.08E-05 | 0.00142 | 35.89 |
| UBASH3A | 9.96 | 1.97 | 0.64 | 3.08 | 0.00209 | 0.04188 | 3.92 |
| CSTB | 197.39 | 0.71 | 0.19 | 3.65 | 0.00026 | 0.01137 | 1.64 |
| TRPM2 | 111.84 | 1.30 | 0.27 | 4.88 | 1.05E-06 | 0.00026 | 2.46 |
| ITGB2 | 564.34 | 1.41 | 0.31 | 4.58 | 4.67E-06 | 0.00076 | 2.66 |
| S100B | 257.25 | -0.90 | 0.28 | -3.23 | 0.00124 | 0.03078 | -1.86 |
| TMEM121B | 6.38 | 1.93 | 0.54 | 3.59 | 0.00034 | 0.01295 | 3.80 |
| BID | 56.47 | 0.73 | 0.25 | 2.99 | 0.00277 | 0.04992 | 1.66 |
| IGLV1-40 | 68.99 | 4.82 | 1.56 | 3.09 | 0.00197 | 0.0403 | 28.30 |
| IGLV3-25 | 51.58 | 6.16 | 1.98 | 3.11 | 0.00187 | 0.03943 | 71.67 |
| IGLV2-11 | 37.35 | 4.55 | 1.39 | 3.28 | 0.00103 | 0.02748 | 23.49 |
| IGLC3 | 270.79 | 5.70 | 1.41 | 4.03 | 5.55E-05 | 0.00416 | 51.85 |
| SGSM1 | 111.48 | -0.85 | 0.24 | -3.51 | 0.00045 | 0.01598 | -1.80 |
| EMID1 | 60.18 | 0.82 | 0.27 | 3.02 | 0.00253 | 0.04746 | 1.76 |
| AP1B1 | 601.54 | 0.59 | 0.19 | 3.10 | 0.00193 | 0.03998 | 1.51 |
| HMOX1 | 297.52 | 1.94 | 0.54 | 3.60 | 0.00031 | 0.01236 | 3.84 |
| APOL1 | 234.13 | 0.66 | 0.16 | 4.20 | 2.62E-05 | 0.00251 | 1.58 |
| NCF4 | 144.31 | 0.71 | 0.22 | 3.20 | 0.00136 | 0.03257 | 1.63 |
| IL2RB | 24.23 | 1.63 | 0.53 | 3.09 | 0.00198 | 0.04032 | 3.10 |
| RAC2 | 73.25 | 1.24 | 0.37 | 3.36 | 0.00079 | 0.02342 | 2.37 |
| CYTH4 | 428.51 | 1.02 | 0.20 | 5.19 | 2.13E-07 | 7.72E-05 | 2.03 |
| KDELR3 | 235.31 | 0.84 | 0.23 | 3.66 | 0.00025 | 0.01132 | 1.79 |
| LINC01315 | 9.57 | -1.23 | 0.32 | -3.83 | 0.00013 | 0.00751 | -2.35 |
| NFAM1 | 89.19 | 1.15 | 0.33 | 3.47 | 0.00052 | 0.01766 | 2.22 |
| SCUBE1 | 6.04 | 3.95 | 0.96 | 4.12 | 3.71E-05 | 0.00319 | 15.50 |
| LINC00899 | 57.48 | -0.86 | 0.27 | -3.18 | 0.00146 | 0.03383 | -1.82 |
| TYMP | 331.98 | 0.80 | 0.26 | 3.06 | 0.0022 | 0.04319 | 1.75 |
| MXRA5 | 4,057.20 | 0.92 | 0.24 | 3.85 | 0.00012 | 0.00715 | 1.89 |
| TLR7 | 123.99 | 1.07 | 0.24 | 4.39 | 1.11E-05 | 0.00144 | 2.10 |
| TLR8 | 67.26 | 1.34 | 0.31 | 4.33 | 1.52E-05 | 0.00184 | 2.54 |
| FANCB | 16.27 | -0.79 | 0.26 | -2.99 | 0.00276 | 0.04992 | -1.73 |
| PIR | 100.51 | -0.62 | 0.20 | -3.04 | 0.00238 | 0.04572 | -1.53 |
| SH3KBP1 | 201.25 | 0.87 | 0.18 | 4.96 | 7.09E-07 | 0.00019 | 1.83 |
| MIR222HG | 30.55 | 0.85 | 0.26 | 3.33 | 0.00086 | 0.02475 | 1.80 |
| TIMP1 | 5,581.74 | 1.10 | 0.29 | 3.77 | 0.00016 | 0.00848 | 2.15 |
| WAS | 53.08 | 1.18 | 0.35 | 3.40 | 0.00067 | 0.02104 | 2.26 |
| VSIG4 | 668.59 | 0.71 | 0.22 | 3.18 | 0.00149 | 0.03404 | 1.64 |
| AR | 330.80 | -0.62 | 0.15 | -4.02 | 5.83E-05 | 0.00426 | -1.54 |
| ITGB1BP2 | 8.04 | 1.41 | 0.47 | 3.02 | 0.00256 | 0.04774 | 2.65 |
| NHSL2 | 272.96 | 0.67 | 0.17 | 3.91 | 9.04E-05 | 0.00586 | 1.60 |
| GPR174 | 18.18 | 1.98 | 0.60 | 3.30 | 0.00097 | 0.02632 | 3.93 |
| ZNF711 | 56.63 | -0.85 | 0.20 | -4.26 | 2.00E-05 | 0.00214 | -1.81 |
| SYTL4 | 99.17 | -0.77 | 0.15 | -5.17 | 2.28E-07 | 8.07E-05 | -1.70 |
| BTK | 70.33 | 0.92 | 0.21 | 4.38 | 1.18E-05 | 0.00151 | 1.90 |
| RADX | 29.04 | -0.85 | 0.28 | -3.03 | 0.00241 | 0.04587 | -1.80 |
| LONRF3 | 91.94 | -0.94 | 0.27 | -3.54 | 0.00039 | 0.01455 | -1.92 |
| ELF4 | 82.36 | 0.82 | 0.26 | 3.17 | 0.0015 | 0.03416 | 1.76 |
| GPC4 | 160.04 | 0.66 | 0.18 | 3.64 | 0.00028 | 0.01159 | 1.58 |
| SMIM10L2A | 27.77 | -0.86 | 0.24 | -3.60 | 0.00031 | 0.01236 | -1.81 |
| BGN | 6,189.20 | 0.89 | 0.29 | 3.10 | 0.00194 | 0.04006 | 1.85 |
| ARHGAP4 | 202.23 | 0.72 | 0.23 | 3.19 | 0.00142 | 0.0334 | 1.65 |
| MT-CO2 | 1,658.01 | 0.60 | 0.19 | 3.23 | 0.00125 | 0.03094 | 1.52 |

Supplementary Table 7: Differentially Expressed Genes in No-CHIP vs Control contrast. Only the genes with a p-value adjusted < 0.05 and a Fold Change < -1.5 and > 1.5 are shown.

| Supplementary Table 8: Differentially Expressed Genes in CHIP vs Control contrast | | | | | | | |
| --- | --- | --- | --- | --- | --- | --- | --- |
| Genes | baseMean | log2FoldChange | lfcSE | stat | pvalue | padj | FoldChange |
| SLC2A5 | 31.71 | 2.02 | 0.47 | 4.26 | 2.00E-05 | 0.004043 | 4.06 |
| SLC9A1 | 96.46 | 0.79 | 0.22 | 3.65 | 0.000258 | 0.023634 | 1.73 |
| HIVEP3 | 71.40 | 0.88 | 0.22 | 3.95 | 7.92E-05 | 0.011042 | 1.83 |
| NFIA-AS2 | 40.32 | -0.98 | 0.26 | -3.76 | 0.000169 | 0.017803 | -1.97 |
| LEPR | 416.78 | -0.82 | 0.22 | -3.67 | 0.000247 | 0.02281 | -1.76 |
| NEXN | 154.44 | 1.14 | 0.28 | 4.15 | 3.40E-05 | 0.005831 | 2.21 |
| GBP1P1 | 52.36 | -0.59 | 0.17 | -3.42 | 0.000619 | 0.041724 | -1.50 |
| F3 | 70.17 | 1.73 | 0.39 | 4.48 | 7.59E-06 | 0.002133 | 3.32 |
| SLC44A3-AS1 | 38.35 | 1.55 | 0.35 | 4.46 | 8.31E-06 | 0.002233 | 2.93 |
| SLC44A3 | 24.97 | 1.43 | 0.32 | 4.49 | 7.03E-06 | 0.002005 | 2.70 |
| VCAM1 | 820.58 | 1.05 | 0.29 | 3.56 | 0.000365 | 0.029788 | 2.07 |
| COL11A1 | 215.25 | 5.58 | 0.86 | 6.51 | 7.38E-11 | 2.72E-07 | 47.93 |
| TCHH | 7.18 | 2.02 | 0.52 | 3.86 | 0.000112 | 0.013834 | 4.05 |
| FCRL5 | 31.89 | 6.04 | 1.42 | 4.25 | 2.09E-05 | 0.004043 | 65.78 |
| SLAMF8 | 113.01 | 1.63 | 0.4 | 4.09 | 4.39E-05 | 0.007201 | 3.10 |
| SLAMF6 | 20.15 | 1.66 | 0.48 | 3.44 | 0.000581 | 0.040514 | 3.15 |
| SLAMF7 | 30.33 | 3.3 | 0.77 | 4.31 | 1.66E-05 | 0.003612 | 9.84 |
| ITLN1 | 20.72 | -1.9 | 0.5 | -3.83 | 0.000127 | 0.015336 | -3.72 |
| ILDR2 | 20.44 | 1.4 | 0.4 | 3.51 | 0.00045 | 0.034037 | 2.63 |
| PRG4 | 679.76 | 2.35 | 0.33 | 7.07 | 1.58E-12 | 1.47E-08 | 5.11 |
| PLA2G4A | 219.25 | -0.86 | 0.21 | -4.2 | 2.64E-05 | 0.004942 | -1.82 |
| LAX1 | 17.14 | 2.57 | 0.65 | 3.96 | 7.65E-05 | 0.010848 | 5.92 |
| LRRN2 | 12.51 | 1.91 | 0.52 | 3.68 | 0.000232 | 0.022205 | 3.76 |
| CR2 | 5.88 | 3.74 | 1.08 | 3.45 | 0.000555 | 0.039118 | 13.41 |
| G0S2 | 81.50 | -1.15 | 0.3 | -3.88 | 0.000105 | 0.013443 | -2.21 |
| IRF6 | 186.81 | -0.76 | 0.2 | -3.7 | 0.000213 | 0.020478 | -1.69 |
| SDC1 | 42.23 | 2.48 | 0.62 | 4.03 | 5.66E-05 | 0.008747 | 5.58 |
| LINC02580 | 79.58 | 0.73 | 0.2 | 3.71 | 0.00021 | 0.020439 | 1.66 |
| ANKRD36BP2 | 49.53 | 6.76 | 1.28 | 5.27 | 1.38E-07 | 9.85E-05 | 108.59 |
| IGKC | 5,923.50 | 7.45 | 1.04 | 7.17 | 7.45E-13 | 1.38E-08 | 175.18 |
| IGKV4-1 | 217.88 | 4.93 | 1.21 | 4.06 | 4.93E-05 | 0.007892 | 30.48 |
| IGKV1-5 | 194.32 | 7.96 | 1.26 | 6.34 | 2.35E-10 | 5.44E-07 | 249.56 |
| IGKV1-9 | 62.88 | 6.9 | 1.57 | 4.39 | 1.14E-05 | 0.002709 | 119.53 |
| IGKV3-11 | 340.37 | 7.85 | 1.27 | 6.17 | 6.74E-10 | 1.39E-06 | 231.34 |
| IGKV1-12 | 44.61 | 7.86 | 1.53 | 5.13 | 2.83E-07 | 0.00017 | 232.90 |
| IGKV3-15 | 55.92 | 7.54 | 1.58 | 4.78 | 1.74E-06 | 0.000674 | 185.81 |
| IGKV1-16 | 12.67 | 6.89 | 1.51 | 4.58 | 4.74E-06 | 0.001568 | 118.76 |
| IGKV3-20 | 527.93 | 7.71 | 1.19 | 6.49 | 8.79E-11 | 2.72E-07 | 209.67 |
| IGKV1-27 | 30.92 | 6.38 | 1.81 | 3.53 | 0.000419 | 0.032543 | 83.31 |
| IGKV1-39 | 52.69 | 8.74 | 1.89 | 4.62 | 3.75E-06 | 0.001265 | 427.94 |
| IGKV1D-39 | 24.62 | 8.2 | 1.93 | 4.26 | 2.08E-05 | 0.004043 | 293.18 |
| IGKV3D-11 | 4.06 | 4.87 | 1.4 | 3.47 | 0.000529 | 0.038429 | 29.16 |
| IL1RL1 | 55.23 | -1.32 | 0.35 | -3.8 | 0.000146 | 0.016348 | -2.49 |
| SLC20A1 | 709.97 | 0.69 | 0.17 | 4.13 | 3.58E-05 | 0.006039 | 1.61 |
| CACNB4 | 133.42 | 0.86 | 0.25 | 3.43 | 0.000599 | 0.041166 | 1.81 |
| KCNH7 | 6.34 | -2.27 | 0.65 | -3.46 | 0.000537 | 0.038561 | -4.81 |
| GALNT3 | 33.51 | 2.37 | 0.5 | 4.71 | 2.47E-06 | 0.000916 | 5.19 |
| CD28 | 176.21 | 0.92 | 0.25 | 3.65 | 0.000259 | 0.023634 | 1.90 |
| ACADL | 72.02 | -0.69 | 0.18 | -3.81 | 0.000139 | 0.016161 | -1.61 |
| FN1 | 102,162.74 | 1.16 | 0.23 | 4.95 | 7.50E-07 | 0.000357 | 2.23 |
| IGFBP2 | 489.70 | 1.73 | 0.45 | 3.89 | 0.000102 | 0.013263 | 3.32 |
| SCG2 | 305.64 | 1.27 | 0.34 | 3.75 | 0.000174 | 0.01805 | 2.41 |
| SLC19A3 | 15.83 | -1.52 | 0.3 | -5.1 | 3.41E-07 | 0.000198 | -2.86 |
| ITIH3 | 52.07 | 1.73 | 0.44 | 3.9 | 9.76E-05 | 0.01292 | 3.32 |
| PRKCD | 92.03 | 0.72 | 0.19 | 3.85 | 0.000118 | 0.014296 | 1.65 |
| COL8A1 | 2,195.58 | 0.83 | 0.23 | 3.68 | 0.000234 | 0.022205 | 1.78 |
| PARP15 | 51.79 | 1.18 | 0.34 | 3.52 | 0.000424 | 0.03279 | 2.27 |
| PLCH1 | 130.11 | -1.16 | 0.32 | -3.61 | 0.000307 | 0.026306 | -2.24 |
| AC117453.1 | 23.29 | 1.83 | 0.52 | 3.52 | 0.000433 | 0.033182 | 3.56 |
| KLHL6 | 78.65 | 1.15 | 0.32 | 3.61 | 0.000304 | 0.026188 | 2.22 |
| AC007920.2 | 19.47 | -1.21 | 0.25 | -4.85 | 1.23E-06 | 0.000531 | -2.31 |
| LRRC15 | 203.65 | 2.5 | 0.65 | 3.82 | 0.000135 | 0.015788 | 5.65 |
| FGFR3 | 73.60 | -0.92 | 0.27 | -3.46 | 0.000535 | 0.038561 | -1.90 |
| ABLIM2 | 25.65 | 1.01 | 0.27 | 3.75 | 0.000176 | 0.018136 | 2.02 |
| LGI2 | 121.67 | 0.71 | 0.21 | 3.36 | 0.000786 | 0.047912 | 1.64 |
| ATP8A1 | 431.58 | -0.87 | 0.24 | -3.68 | 0.000235 | 0.02226 | -1.83 |
| KIT | 62.92 | 1.43 | 0.38 | 3.78 | 0.00016 | 0.017383 | 2.69 |
| JCHAIN | 649.09 | 5.68 | 1.04 | 5.44 | 5.38E-08 | 4.99E-05 | 51.17 |
| RASSF6 | 9.45 | 6.77 | 1.73 | 3.92 | 8.88E-05 | 0.011931 | 109.29 |
| CXCL13 | 5.58 | 5.06 | 1.35 | 3.76 | 0.000173 | 0.018039 | 33.39 |
| ANXA3 | 108.04 | -1.02 | 0.23 | -4.42 | 9.94E-06 | 0.002526 | -2.03 |
| IBSP | 78.16 | 4.19 | 1 | 4.2 | 2.72E-05 | 0.005049 | 18.24 |
| SPP1 | 2,847.46 | 2.97 | 0.86 | 3.45 | 0.000554 | 0.039118 | 7.81 |
| AC106881.1 | 21.50 | -0.8 | 0.24 | -3.4 | 0.000682 | 0.043904 | -1.74 |
| TDO2 | 17.48 | 3.66 | 1 | 3.65 | 0.000263 | 0.023634 | 12.62 |
| APELA | 7.70 | 4.22 | 1.12 | 3.77 | 0.000164 | 0.017383 | 18.66 |
| ZDHHC11B | 64.19 | -1.86 | 0.4 | -4.65 | 3.31E-06 | 0.001137 | -3.64 |
| ZDHHC11 | 33.29 | -1.29 | 0.34 | -3.83 | 0.000129 | 0.015478 | -2.44 |
| MYO10 | 155.82 | 1.01 | 0.3 | 3.4 | 0.000678 | 0.043792 | 2.02 |
| AC010343.3 | 6.26 | 3.71 | 1.06 | 3.5 | 0.000466 | 0.034878 | 13.09 |
| GDNF | 14.95 | 1.18 | 0.35 | 3.34 | 0.000832 | 0.049752 | 2.27 |
| FYB1 | 262.75 | 0.76 | 0.22 | 3.5 | 0.00046 | 0.034677 | 1.70 |
| SPOCK1 | 304.63 | 1.26 | 0.31 | 4.06 | 4.89E-05 | 0.007892 | 2.40 |
| MZB1 | 35.07 | 6.5 | 1.4 | 4.65 | 3.25E-06 | 0.001137 | 90.77 |
| GRIA1 | 6.25 | 5.34 | 1.25 | 4.27 | 1.93E-05 | 0.004012 | 40.62 |
| ATP10B | 8.63 | 4.65 | 1.34 | 3.48 | 0.000493 | 0.036273 | 25.17 |
| DOK3 | 58.13 | 1.2 | 0.35 | 3.47 | 0.000515 | 0.037777 | 2.30 |
| IRF4 | 43.42 | 2.33 | 0.68 | 3.43 | 0.000597 | 0.041139 | 5.04 |
| TUBB2B | 64.20 | -1.07 | 0.3 | -3.58 | 0.000345 | 0.028571 | -2.10 |
| PHACTR1 | 49.89 | 0.84 | 0.18 | 4.57 | 4.85E-06 | 0.001578 | 1.79 |
| C2 | 121.80 | 0.71 | 0.2 | 3.57 | 0.000363 | 0.029743 | 1.64 |
| C4A | 91.56 | 1.27 | 0.3 | 4.22 | 2.49E-05 | 0.004718 | 2.41 |
| SCUBE3 | 28.66 | 1.7 | 0.46 | 3.67 | 0.000246 | 0.02281 | 3.25 |
| MAPK13 | 43.00 | 1.68 | 0.32 | 5.24 | 1.63E-07 | 0.000108 | 3.20 |
| PRDM1 | 57.98 | 1.41 | 0.32 | 4.46 | 8.26E-06 | 0.002233 | 2.65 |
| CRYBG1 | 208.99 | 0.84 | 0.24 | 3.55 | 0.000388 | 0.030972 | 1.79 |
| ROS1 | 5.71 | 5.5 | 1.22 | 4.51 | 6.55E-06 | 0.001898 | 45.32 |
| TMEM200A | 52.72 | 1.51 | 0.38 | 4.01 | 6.20E-05 | 0.009266 | 2.85 |
| ENPP1 | 399.37 | 1.49 | 0.29 | 5.06 | 4.22E-07 | 0.000215 | 2.80 |
| UST | 233.96 | -0.66 | 0.19 | -3.49 | 0.000476 | 0.03517 | -1.58 |
| SMOC2 | 291.53 | 1.58 | 0.27 | 5.93 | 2.99E-09 | 4.26E-06 | 2.99 |
| THBS2 | 6,911.45 | 0.98 | 0.25 | 3.86 | 0.000114 | 0.013851 | 1.97 |
| MPP6 | 384.45 | -0.69 | 0.2 | -3.46 | 0.000548 | 0.039095 | -1.61 |
| TRG-AS1 | 10.25 | 1.98 | 0.58 | 3.42 | 0.000634 | 0.042382 | 3.95 |
| STEAP1 | 102.10 | 0.75 | 0.22 | 3.39 | 0.000689 | 0.044022 | 1.68 |
| PDK4 | 852.63 | -1.05 | 0.31 | -3.42 | 0.000635 | 0.042382 | -2.07 |
| BHLHA15 | 8.99 | 5.46 | 1.6 | 3.42 | 0.000632 | 0.042382 | 44.02 |
| AC018638.4 | 40.19 | 0.98 | 0.29 | 3.4 | 0.000677 | 0.043792 | 1.97 |
| SLC7A2 | 165.42 | 1.65 | 0.33 | 5.06 | 4.09E-07 | 0.000215 | 3.14 |
| EBF2 | 10.21 | 2.15 | 0.61 | 3.51 | 0.000443 | 0.033765 | 4.44 |
| SLCO5A1 | 11.10 | 1.61 | 0.44 | 3.65 | 0.00026 | 0.023634 | 3.04 |
| STMN2 | 78.27 | 2.93 | 0.53 | 5.48 | 4.14E-08 | 4.79E-05 | 7.62 |
| TNFRSF11B | 779.98 | 1.04 | 0.25 | 4.13 | 3.55E-05 | 0.006039 | 2.06 |
| COLEC10 | 7.49 | 2.93 | 0.71 | 4.11 | 3.94E-05 | 0.006515 | 7.61 |
| LINC00861 | 14.87 | 2.52 | 0.73 | 3.43 | 0.000607 | 0.041349 | 5.72 |
| ASAP1 | 486.13 | 0.69 | 0.12 | 5.56 | 2.64E-08 | 3.26E-05 | 1.62 |
| CCN4 | 362.57 | 1.27 | 0.32 | 3.94 | 8.32E-05 | 0.011508 | 2.41 |
| PAX5 | 3.51 | 4.67 | 1.36 | 3.43 | 0.000603 | 0.041271 | 25.37 |
| OGN | 592.76 | 0.98 | 0.2 | 4.85 | 1.22E-06 | 0.000531 | 1.97 |
| CTNNAL1 | 244.35 | -0.65 | 0.18 | -3.54 | 0.000403 | 0.031496 | -1.57 |
| TNC | 6,181.37 | 1.67 | 0.33 | 5 | 5.74E-07 | 0.00028 | 3.18 |
| CERCAM | 553.09 | 0.59 | 0.16 | 3.76 | 0.000171 | 0.017906 | 1.51 |
| LINC01503 | 29.15 | 0.98 | 0.24 | 4.18 | 2.93E-05 | 0.005218 | 1.98 |
| RAPGEF1 | 363.71 | 0.69 | 0.17 | 3.96 | 7.43E-05 | 0.010676 | 1.61 |
| TMEM236 | 23.79 | 0.93 | 0.27 | 3.47 | 0.00052 | 0.037964 | 1.91 |
| KIAA1217 | 104.45 | 1.24 | 0.32 | 3.86 | 0.000113 | 0.013851 | 2.36 |
| LINC01515 | 40.44 | -1.59 | 0.4 | -4 | 6.26E-05 | 0.009289 | -3.00 |
| MYOZ1 | 11.68 | 2.89 | 0.65 | 4.42 | 9.76E-06 | 0.002514 | 7.40 |
| CRTAC1 | 1,520.41 | 0.96 | 0.23 | 4.19 | 2.84E-05 | 0.005164 | 1.95 |
| SH3PXD2A | 719.44 | 0.66 | 0.16 | 4.13 | 3.69E-05 | 0.006166 | 1.58 |
| ADRA2A | 70.80 | 2.57 | 0.6 | 4.26 | 2.07E-05 | 0.004043 | 5.94 |
| PLEKHS1 | 20.06 | 5.06 | 0.92 | 5.47 | 4.39E-08 | 4.79E-05 | 33.25 |
| NANOS1 | 121.50 | -1.24 | 0.33 | -3.8 | 0.000146 | 0.016348 | -2.36 |
| IFITM10 | 35.73 | 1.59 | 0.38 | 4.15 | 3.36E-05 | 0.005831 | 3.02 |
| AC068580.4 | 55.02 | 1.9 | 0.37 | 5.09 | 3.65E-07 | 0.000205 | 3.74 |
| TPH1 | 13.35 | 1.04 | 0.31 | 3.38 | 0.000728 | 0.045288 | 2.06 |
| TP53I11 | 95.67 | 1.21 | 0.34 | 3.58 | 0.000338 | 0.028222 | 2.31 |
| SYT12 | 205.96 | 1.71 | 0.37 | 4.66 | 3.09E-06 | 0.001122 | 3.26 |
| CHRDL2 | 28.92 | 2.51 | 0.69 | 3.63 | 0.000279 | 0.024507 | 5.70 |
| MMP1 | 7.81 | 4.41 | 1.14 | 3.88 | 0.000106 | 0.013514 | 21.32 |
| POU2AF1 | 14.50 | 6.25 | 1.43 | 4.36 | 1.29E-05 | 0.002943 | 76.13 |
| CD27 | 11.60 | 3.12 | 0.83 | 3.77 | 0.000162 | 0.017383 | 8.69 |
| A2M | 5,817.86 | 0.81 | 0.18 | 4.52 | 6.31E-06 | 0.001857 | 1.75 |
| PTHLH | 99.35 | -1.42 | 0.37 | -3.82 | 0.000135 | 0.015788 | -2.68 |
| VDR | 82.90 | 1.16 | 0.3 | 3.89 | 0.000102 | 0.013263 | 2.24 |
| ITGB7 | 29.54 | 1.2 | 0.34 | 3.51 | 0.000445 | 0.033786 | 2.29 |
| SP7 | 8.98 | 4.41 | 1.24 | 3.55 | 0.000386 | 0.03095 | 21.32 |
| AVIL | 96.34 | 1.01 | 0.23 | 4.39 | 1.13E-05 | 0.002709 | 2.01 |
| IFNG-AS1 | 6.76 | 2.69 | 0.8 | 3.37 | 0.000757 | 0.046614 | 6.44 |
| TRHDE | 155.63 | -1.03 | 0.24 | -4.27 | 1.91E-05 | 0.004012 | -2.04 |
| ALDH1L2 | 282.45 | 0.59 | 0.15 | 4.05 | 5.11E-05 | 0.008101 | 1.51 |
| WSCD2 | 38.45 | 1.24 | 0.37 | 3.38 | 0.000714 | 0.044902 | 2.36 |
| CMKLR1 | 722.13 | 0.81 | 0.17 | 4.81 | 1.49E-06 | 0.000602 | 1.75 |
| TMEM119 | 177.38 | 1.08 | 0.27 | 4.06 | 4.94E-05 | 0.007892 | 2.12 |
| BICDL1 | 7.08 | 2.56 | 0.75 | 3.4 | 0.000674 | 0.043792 | 5.88 |
| TNFSF11 | 11.01 | 2.62 | 0.76 | 3.44 | 0.000577 | 0.040383 | 6.16 |
| SLAIN1 | 52.69 | -0.75 | 0.2 | -3.71 | 0.000211 | 0.020439 | -1.68 |
| TMEM255B | 28.03 | 0.97 | 0.26 | 3.77 | 0.000162 | 0.017383 | 1.96 |
| SLC7A8 | 100.07 | 0.93 | 0.22 | 4.15 | 3.31E-05 | 0.005831 | 1.91 |
| LRFN5 | 48.57 | -0.93 | 0.28 | -3.38 | 0.000722 | 0.045126 | -1.91 |
| GPR68 | 56.18 | 2.02 | 0.45 | 4.53 | 5.89E-06 | 0.001833 | 4.06 |
| TC2N | 364.68 | -0.7 | 0.19 | -3.7 | 0.000213 | 0.020478 | -1.63 |
| CLMN | 148.40 | 0.84 | 0.19 | 4.37 | 1.24E-05 | 0.002913 | 1.79 |
| AHNAK2 | 1,541.78 | 0.74 | 0.19 | 3.93 | 8.65E-05 | 0.011767 | 1.67 |
| IGHG4 | 483.40 | 10.11 | 1.76 | 5.76 | 8.62E-09 | 1.14E-05 | 1,102.67 |
| IGHG2 | 2,277.83 | 8.32 | 1.22 | 6.82 | 9.31E-12 | 4.31E-08 | 320.07 |
| IGHGP | 273.40 | 8.35 | 1.62 | 5.16 | 2.42E-07 | 0.00015 | 325.97 |
| IGHA1 | 1,032.46 | 7.3 | 1.04 | 7 | 2.53E-12 | 1.56E-08 | 157.87 |
| IGHG1 | 3,712.26 | 7.17 | 1.12 | 6.4 | 1.55E-10 | 4.09E-07 | 144.18 |
| IGHG3 | 317.65 | 7.06 | 1.18 | 5.96 | 2.47E-09 | 3.82E-06 | 133.51 |
| FAM30A | 36.12 | 8.12 | 1.61 | 5.06 | 4.28E-07 | 0.000215 | 278.53 |
| IGHV1-2 | 16.79 | 6.8 | 1.5 | 4.54 | 5.63E-06 | 0.0018 | 111.36 |
| IGHV1-3 | 16.91 | 7.58 | 1.89 | 4.02 | 5.87E-05 | 0.008979 | 191.70 |
| IGHV3-7 | 36.43 | 7.08 | 1.47 | 4.82 | 1.41E-06 | 0.000593 | 134.93 |
| IGHV3-11 | 15.03 | 5.57 | 1.64 | 3.4 | 0.000685 | 0.043967 | 47.48 |
| IGHV3-15 | 27.82 | 6.42 | 1.46 | 4.39 | 1.13E-05 | 0.002709 | 85.41 |
| IGHV1-18 | 18.49 | 4.81 | 1.31 | 3.67 | 0.00024 | 0.022433 | 28.03 |
| IGHV3-21 | 18.35 | 4.73 | 1.39 | 3.41 | 0.000655 | 0.043327 | 26.51 |
| IGHV3-23 | 236.29 | 8.23 | 1.37 | 6 | 1.92E-09 | 3.24E-06 | 301.15 |
| IGHV1-24 | 18.07 | 7.68 | 1.95 | 3.95 | 7.89E-05 | 0.011042 | 204.88 |
| IGHV3-30 | 54.44 | 5.96 | 1.4 | 4.27 | 1.95E-05 | 0.004012 | 62.22 |
| IGHV4-31 | 9.69 | 6.08 | 1.81 | 3.35 | 0.0008 | 0.048601 | 67.69 |
| IGHV3-33 | 23.74 | 7.47 | 1.55 | 4.82 | 1.47E-06 | 0.000602 | 177.46 |
| IGHV4-34 | 18.02 | 7.39 | 1.71 | 4.31 | 1.62E-05 | 0.003578 | 167.90 |
| IGHV4-39 | 43.89 | 7.38 | 1.38 | 5.35 | 8.60E-08 | 6.93E-05 | 166.07 |
| IGHV1-46 | 6.97 | 6.21 | 1.67 | 3.72 | 0.0002 | 0.019654 | 73.99 |
| IGHV3-48 | 36.97 | 8.23 | 1.82 | 4.52 | 6.07E-06 | 0.001846 | 300.09 |
| IGHV3-49 | 10.95 | 6.38 | 1.65 | 3.87 | 0.000111 | 0.013834 | 83.35 |
| IGHV5-51 | 63.41 | 8.05 | 1.68 | 4.8 | 1.57E-06 | 0.000621 | 264.25 |
| IGHV4-59 | 75.55 | 8.01 | 1.48 | 5.4 | 6.57E-08 | 5.80E-05 | 258.28 |
| HERC2P3 | 73.51 | 1.48 | 0.4 | 3.73 | 0.000189 | 0.018893 | 2.79 |
| HDC | 18.40 | 1.97 | 0.58 | 3.38 | 0.000723 | 0.045126 | 3.93 |
| CA12 | 84.21 | 1.55 | 0.4 | 3.92 | 9.03E-05 | 0.012044 | 2.93 |
| ANPEP | 265.77 | 1.34 | 0.27 | 5.06 | 4.25E-07 | 0.000215 | 2.54 |
| MCTP2 | 56.13 | 0.93 | 0.26 | 3.65 | 0.000267 | 0.023759 | 1.91 |
| AC104260.2 | 14.14 | 1.4 | 0.41 | 3.4 | 0.000677 | 0.043792 | 2.64 |
| TPSB2 | 31.41 | 3.14 | 0.71 | 4.41 | 1.03E-05 | 0.002589 | 8.80 |
| TPSAB1 | 31.42 | 2.16 | 0.58 | 3.74 | 0.000185 | 0.018782 | 4.46 |
| AC099489.1 | 23.91 | 0.95 | 0.27 | 3.55 | 0.000391 | 0.030992 | 1.94 |
| IL21R | 35.49 | 2.05 | 0.55 | 3.75 | 0.00018 | 0.018441 | 4.13 |
| NETO2 | 58.19 | -1.19 | 0.28 | -4.23 | 2.29E-05 | 0.004382 | -2.27 |
| WFDC1 | 54.45 | 1.18 | 0.33 | 3.61 | 0.000308 | 0.026306 | 2.26 |
| SMTNL2 | 16.84 | -1.77 | 0.52 | -3.39 | 0.000699 | 0.044374 | -3.40 |
| DHRS13 | 24.75 | -0.69 | 0.21 | -3.35 | 0.000813 | 0.048922 | -1.62 |
| EVI2B | 42.54 | 1.01 | 0.3 | 3.41 | 0.000647 | 0.042985 | 2.01 |
| MYO1D | 2,236.15 | 0.85 | 0.25 | 3.46 | 0.000545 | 0.039014 | 1.80 |
| CCL8 | 32.51 | 1.49 | 0.43 | 3.45 | 0.000566 | 0.039753 | 2.80 |
| IKZF3 | 68.38 | 2.16 | 0.61 | 3.54 | 0.000395 | 0.031063 | 4.48 |
| KRT16 | 12.30 | 2.59 | 0.62 | 4.19 | 2.78E-05 | 0.005111 | 6.03 |
| AOC3 | 115.82 | 1.38 | 0.26 | 5.29 | 1.21E-07 | 9.37E-05 | 2.61 |
| HOXB6 | 12.60 | 1.51 | 0.36 | 4.18 | 2.90E-05 | 0.005218 | 2.84 |
| ABCC3 | 280.62 | 0.88 | 0.24 | 3.6 | 0.00032 | 0.027098 | 1.84 |
| FAM20A | 190.76 | 1.09 | 0.27 | 4.02 | 5.91E-05 | 0.008979 | 2.12 |
| GREB1L | 4.14 | -1.97 | 0.52 | -3.8 | 0.000144 | 0.016348 | -3.92 |
| DCC | 12.17 | 7.17 | 1.47 | 4.88 | 1.09E-06 | 0.000504 | 143.89 |
| ARID3A | 45.25 | 0.87 | 0.2 | 4.26 | 2.06E-05 | 0.004043 | 1.83 |
| DIRAS1 | 22.44 | 1.38 | 0.35 | 3.99 | 6.63E-05 | 0.009678 | 2.61 |
| MFSD12 | 200.99 | 0.66 | 0.17 | 3.96 | 7.43E-05 | 0.010676 | 1.58 |
| STXBP2 | 27.28 | 1.2 | 0.34 | 3.54 | 0.000395 | 0.031063 | 2.29 |
| PDE4A | 127.11 | 0.67 | 0.19 | 3.44 | 0.000585 | 0.040601 | 1.59 |
| HSH2D | 9.11 | 2.8 | 0.77 | 3.64 | 0.000274 | 0.024256 | 6.95 |
| CRLF1 | 370.18 | 1.45 | 0.27 | 5.44 | 5.29E-08 | 4.99E-05 | 2.73 |
| TMEM59L | 35.25 | 0.91 | 0.23 | 4.01 | 6.12E-05 | 0.009227 | 1.88 |
| COMP | 2,826.05 | 1.48 | 0.34 | 4.35 | 1.38E-05 | 0.003123 | 2.79 |
| CILP2 | 77.81 | 1.69 | 0.45 | 3.8 | 0.000147 | 0.016359 | 3.23 |
| FCGBP | 1,969.37 | 1.96 | 0.44 | 4.41 | 1.05E-05 | 0.0026 | 3.88 |
| CD79A | 21.43 | 4.69 | 1.09 | 4.29 | 1.81E-05 | 0.003849 | 25.88 |
| POU2F2 | 42.55 | 1.36 | 0.32 | 4.29 | 1.76E-05 | 0.003804 | 2.57 |
| KCNN4 | 68.25 | 1.64 | 0.35 | 4.76 | 1.89E-06 | 0.000716 | 3.13 |
| LRRC4B | 37.23 | 1.16 | 0.31 | 3.8 | 0.000142 | 0.016346 | 2.24 |
| CD93 | 1,269.85 | 0.93 | 0.27 | 3.42 | 0.000615 | 0.041724 | 1.90 |
| SLPI | 96.09 | 1.82 | 0.4 | 4.52 | 6.24E-06 | 0.001857 | 3.53 |
| KCNG1 | 16.08 | -0.85 | 0.25 | -3.41 | 0.000657 | 0.043327 | -1.80 |
| CBLN4 | 6.64 | 4.19 | 1.2 | 3.5 | 0.000472 | 0.035099 | 18.25 |
| PCK1 | 5.39 | -3.74 | 0.62 | -6.04 | 1.52E-09 | 2.81E-06 | -13.35 |
| RUNX1 | 768.24 | 0.74 | 0.18 | 4 | 6.38E-05 | 0.009389 | 1.67 |
| KCNJ6 | 13.49 | 5.96 | 1.13 | 5.28 | 1.30E-07 | 9.62E-05 | 62.36 |
| SPATA20P1 | 4.02 | 4.43 | 1.16 | 3.82 | 0.000132 | 0.015645 | 21.50 |
| TRPM2 | 111.84 | 0.96 | 0.25 | 3.8 | 0.000146 | 0.016348 | 1.95 |
| IGLV6-57 | 28.02 | 6.51 | 1.69 | 3.86 | 0.000112 | 0.013834 | 91.16 |
| IGLV1-47 | 28.96 | 6.12 | 1.64 | 3.74 | 0.000186 | 0.018783 | 69.61 |
| IGLV1-44 | 53.33 | 6.24 | 1.6 | 3.89 | 9.89E-05 | 0.013006 | 75.56 |
| IGLV1-40 | 68.99 | 5.54 | 1.48 | 3.74 | 0.000183 | 0.018654 | 46.53 |
| IGLV3-25 | 51.58 | 9.19 | 1.89 | 4.85 | 1.22E-06 | 0.000531 | 584.38 |
| IGLV2-23 | 64.15 | 5.95 | 1.52 | 3.92 | 8.69E-05 | 0.011767 | 61.90 |
| IGLV3-21 | 83.61 | 7.66 | 1.46 | 5.25 | 1.49E-07 | 0.000102 | 202.81 |
| IGLV3-19 | 76.63 | 7.92 | 1.7 | 4.65 | 3.31E-06 | 0.001137 | 242.06 |
| IGLV2-14 | 106.06 | 6.6 | 1.23 | 5.36 | 8.43E-08 | 6.93E-05 | 97.04 |
| IGLV2-11 | 37.35 | 5.88 | 1.33 | 4.43 | 9.23E-06 | 0.00241 | 58.96 |
| IGLV3-10 | 8.96 | 6.7 | 1.69 | 3.95 | 7.66E-05 | 0.010848 | 103.96 |
| IGLV3-1 | 29.12 | 5.08 | 1.37 | 3.72 | 0.0002 | 0.019654 | 33.90 |
| IGLL5 | 8.65 | 6.56 | 1.83 | 3.59 | 0.000333 | 0.02795 | 94.68 |
| IGLC2 | 514.97 | 6.3 | 1.15 | 5.46 | 4.83E-08 | 4.98E-05 | 78.63 |
| IGLC3 | 270.79 | 6.94 | 1.34 | 5.18 | 2.26E-07 | 0.000145 | 122.97 |
| DERL3 | 24.23 | 3.95 | 0.9 | 4.37 | 1.26E-05 | 0.002928 | 15.42 |
| MIF-AS1 | 11.75 | 2.08 | 0.59 | 3.52 | 0.000432 | 0.033182 | 4.23 |
| C22orf46 | 60.54 | 0.69 | 0.19 | 3.73 | 0.00019 | 0.018905 | 1.62 |
| SH3KBP1 | 201.25 | 0.7 | 0.17 | 4.15 | 3.37E-05 | 0.005831 | 1.62 |
| TSPAN7 | 53.42 | -1.16 | 0.33 | -3.5 | 0.000473 | 0.035099 | -2.24 |
| PIM2 | 103.91 | 2.24 | 0.58 | 3.88 | 0.000104 | 0.013443 | 4.73 |
| NHSL2 | 272.96 | 0.73 | 0.16 | 4.44 | 9.08E-06 | 0.002406 | 1.65 |
| BGN | 6,189.20 | 0.93 | 0.27 | 3.42 | 0.000617 | 0.041724 | 1.90 |
| MT-ATP8 | 58.48 | 0.97 | 0.25 | 3.86 | 0.000112 | 0.013834 | 1.97 |
| MT-ATP6 | 757.32 | 0.82 | 0.24 | 3.4 | 0.00067 | 0.043792 | 1.77 |
| MT-ND4 | 1,535.88 | 0.8 | 0.24 | 3.35 | 0.000802 | 0.048601 | 1.74 |
| AC233755.1 | 14.20 | 7.42 | 1.97 | 3.77 | 0.000164 | 0.017383 | 171.39 |

Supplementary Table 8: Differentially Expressed Genes in CHIP vs Control contrast. Only the genes with a p-value adjusted < 0.05 and a Fold Change < -1.5 and > 1.5 are shown.

| **Supplementary Table 9: Differentially Expressed Genes in CHIP vs No-CHIP contrast** | | | | | | | |
| --- | --- | --- | --- | --- | --- | --- | --- |
| **Genes** | **baseMean** | **log2FoldChange** | **lfcSE** | **stat** | **pvalue** | **padj** | **FoldChange** |
| IGKC | 5,923.50 | 3.88 | 0.89 | 4.34 | 1.43E-05 | 0.03853 | 14.68 |
| IGKV1-12 | 44.61 | 4.92 | 1.12 | 4.38 | 1.18E-05 | 0.03853 | 30.33 |
| COL12A1 | 5,250.82 | -0.67 | 0.16 | -4.25 | 2.16E-05 | 0.04205 | -1.59 |
| AXIN2 | 145.66 | 0.62 | 0.15 | 4.22 | 2.40E-05 | 0.04205 | 1.54 |
| PCSK1N | 9.50 | -2.15 | 0.50 | -4.33 | 1.47E-05 | 0.03853 | -4.44 |

Supplementary Table 9: Differentially Expressed Genes in CHIP vs No-CHIP contrast. Only the genes with a p-value adjusted < 0.05 and a Fold Change < -1.5 and > 1.5 are shown.

**Supplementary Figures**

Supplementary Figure 1


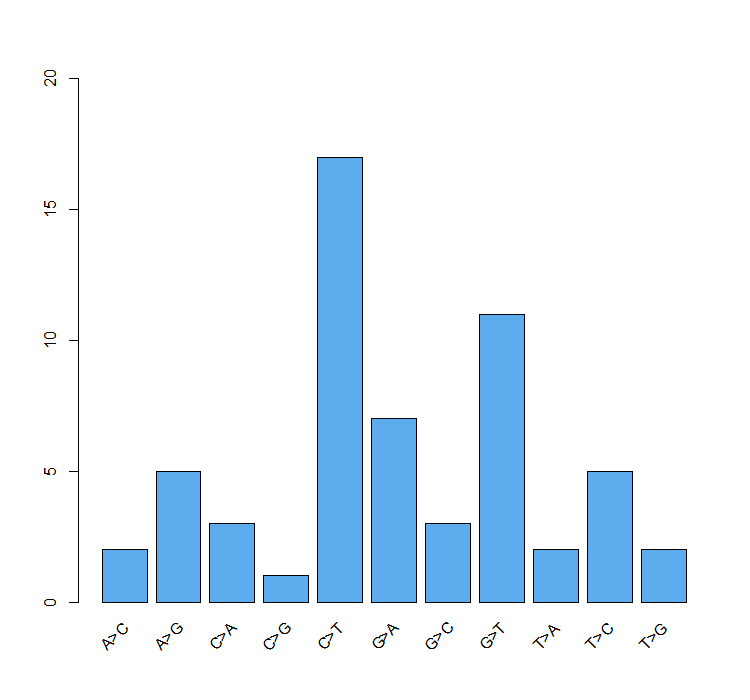


Supplementary Figure 1: Bar-Plot of the nucleotide substitutions of all the identified variants.

Supplementary Figure 2


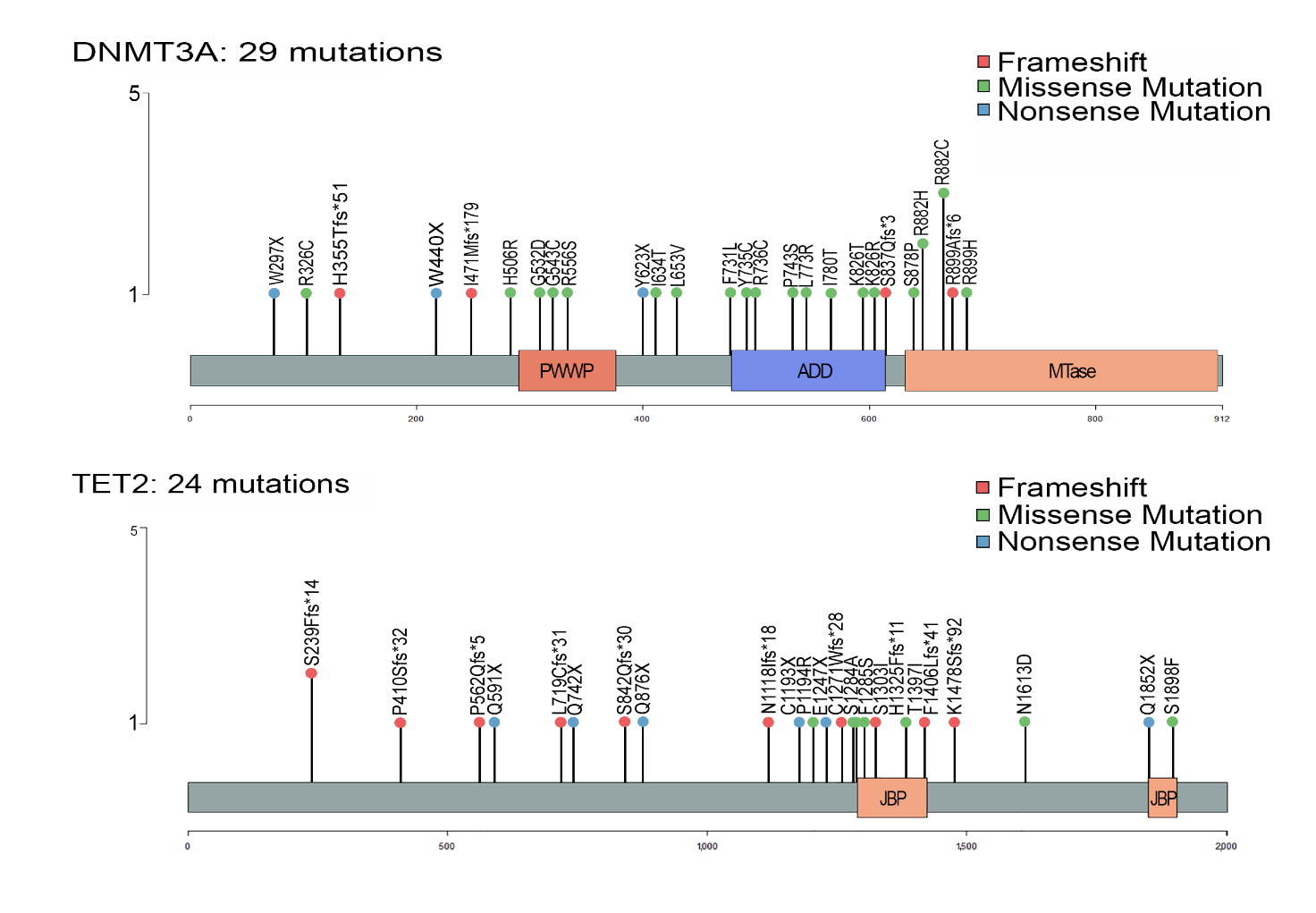


Supplementary Figure 2: DNMT3A and TET2 variants associated with CHIP. Each variant is represented with different colors depending on the type of mutation..

Supplementary Figure 3


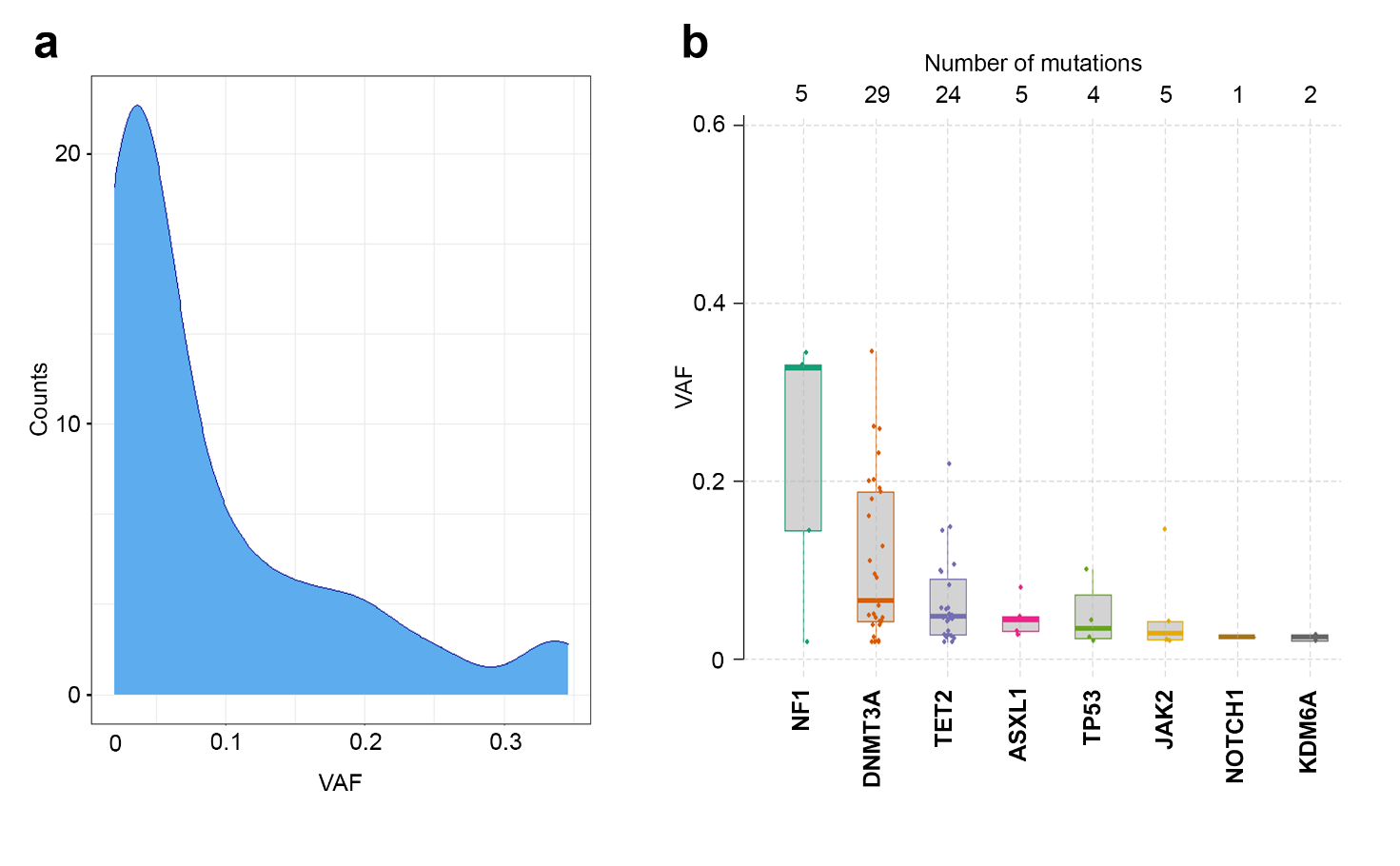


Supplementary Figure 3: Variant Allele Frequency distribution. a) Box plot of VAF for each sequenced gene. On the top, the total number of mutations per gene is shown. b) VAF distribution in the CHIP-related variants.

Supplementary Figure 4


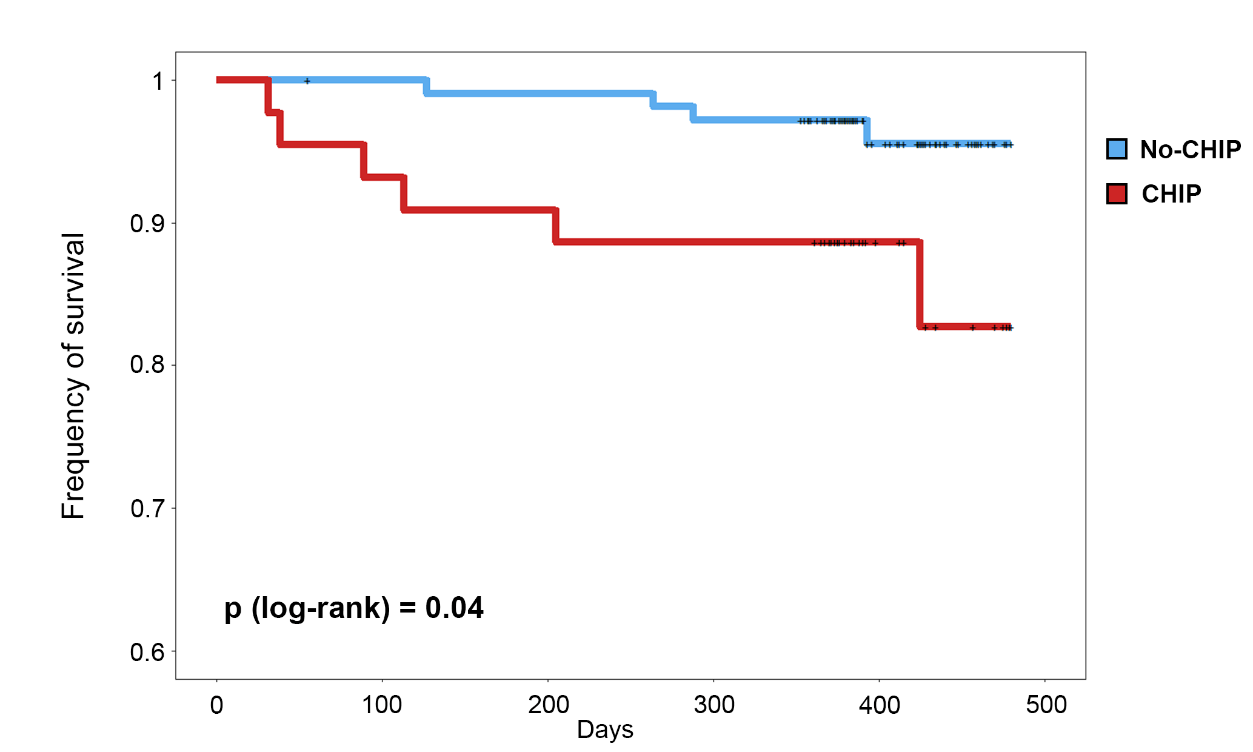


Supplementary Figure 4: Kaplan-Meier plot excluding deaths for non-cardiovascular events. CHIP and no-CHIP that are represented in red and blue, respectively. P-value of the log-rank test is shown in the lower left corner.

Supplementary Figure 5


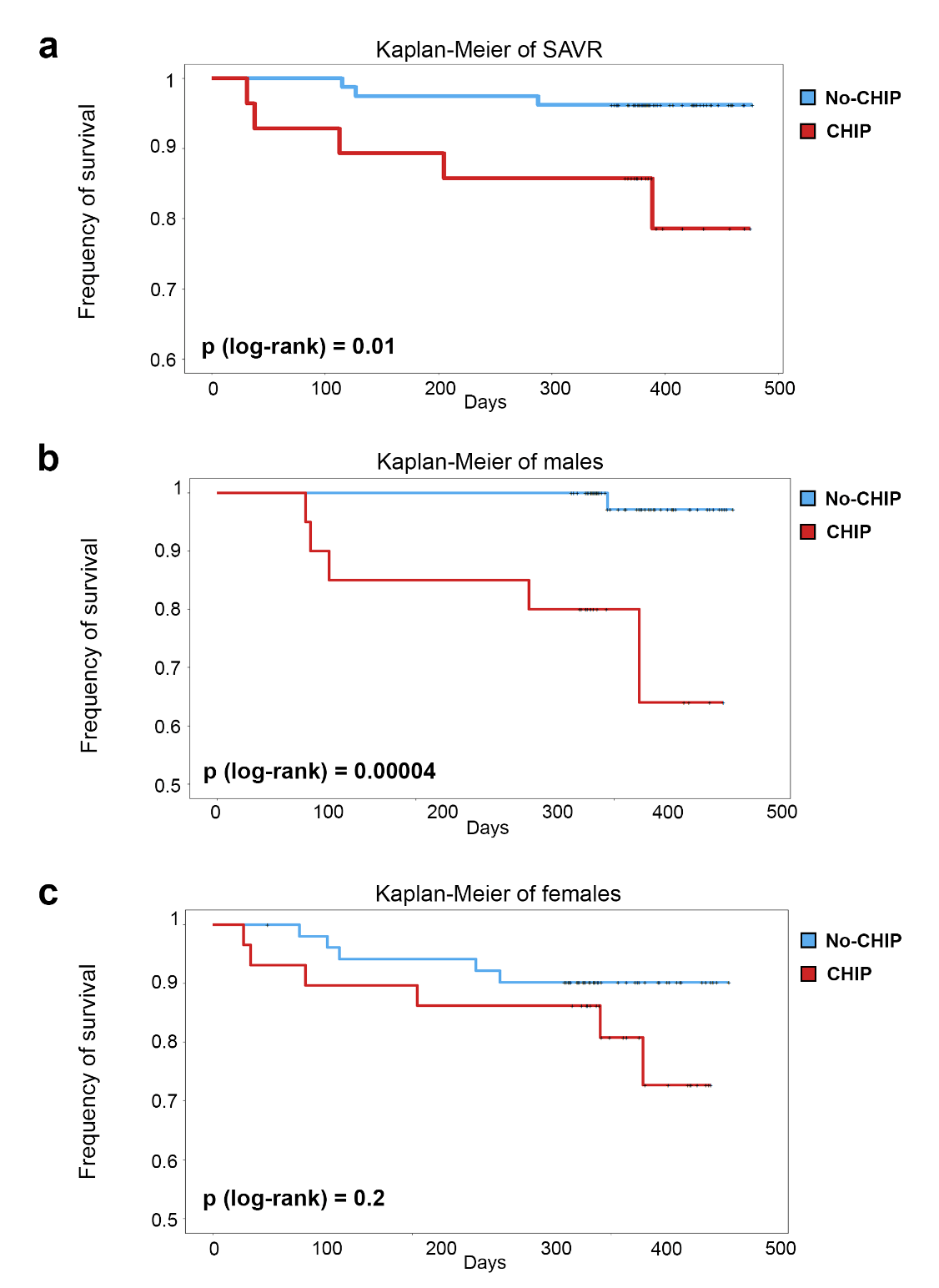


Supplementary Figure 5: Kaplan-Meier plots for subgroups of the original cohort. CHIP and no-CHIP are represented in red and blue, respectively. P-value of the log-rank test is shown in the lower left corner. a) Survival plot of patients that removed the aortic valve through a surgical approach. b) Survival plot of only males. c) Survival plot of only females.

Supplementary Figure 6


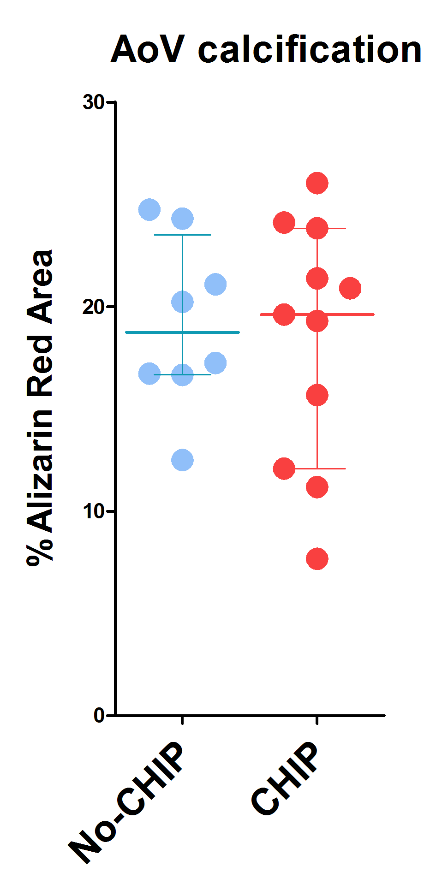


Supplementary Figure 6: Calcification levels in the aortic valves of (AoV) of samples from CHIP or no-CHIP patients. The level of calcification was expressed as the % of area of Alizarin red staining.

Supplementary Figure 7


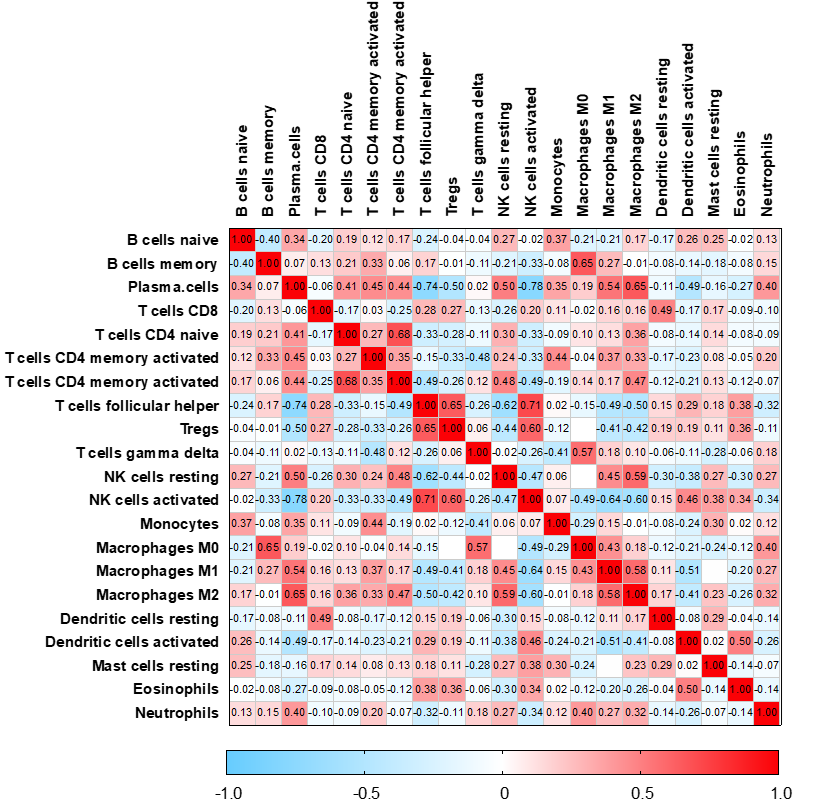


Supplementary Figure 7: Correlation matrix of CIBERSORTx estimated counts. Colors range from blue (anti-correlated) to red (correlated).

Supplementary Figure 8


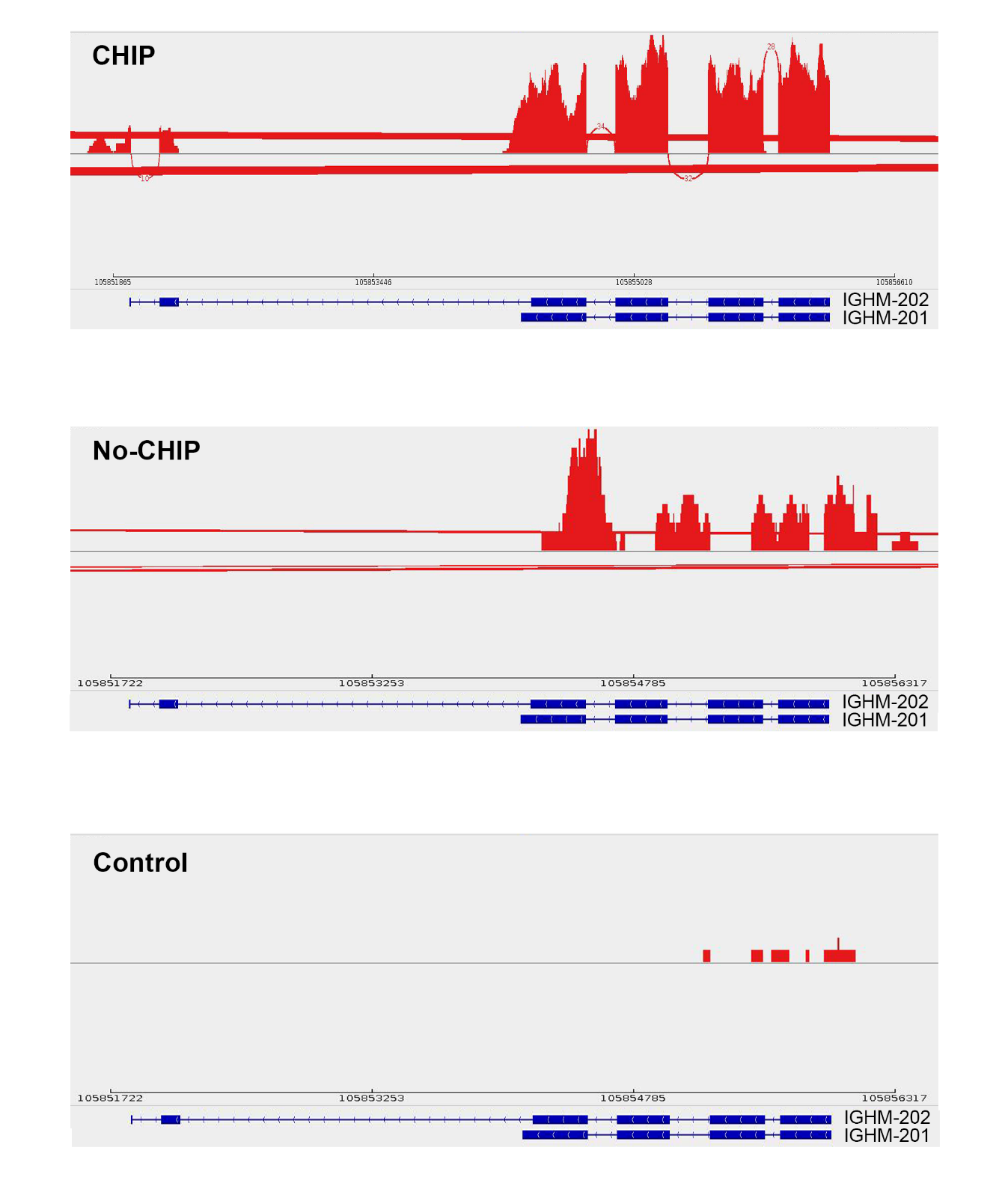


Supplementary Figure 8: Representative Sashimi plot of three samples (one for each group). The two isoforms of IGHM (IGHM-202 and IGHM-201) gene are shown in blue. The red peaks represent the coverage, and the red lines the presence of junctions. Only the CHIP and No-CHIP have high coverage on IGHM gene, but only CHIP samples show the presence of coverage in the extreme exon of the IGHM-202 isoform.

Supplementary Figure 9


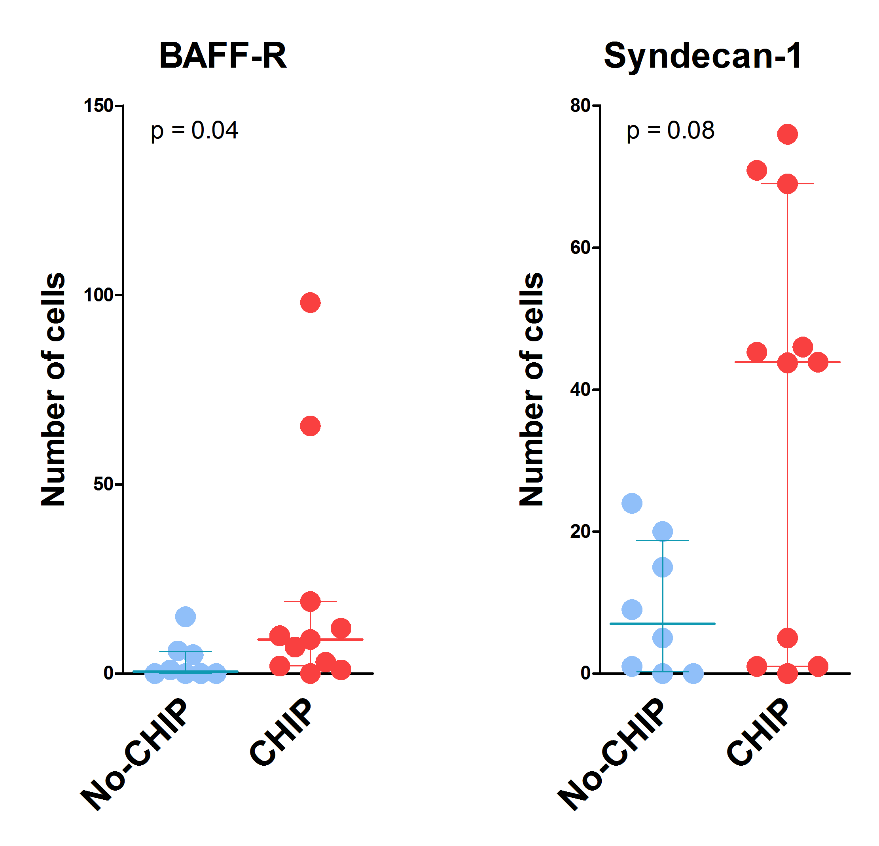


Supplementary Figure 9. Quantification of BAFF-R and Syndecan-1 positive cells in immunohistochemistry samples of aortic valves from CHIP or No-CHIP patients.

**Supplementary Methods**

1. **Detection of CHIP**

After genomic DNA (gDNA) extraction, quality control checks were performed: gDNA concentration was assessed using the Qubit dsDNA BR Assay Kit (ThermoFisher Scientific, Waltham, MA, USA) on a Qubit 2.0 Fluorometer (ThermoFisher Scientific, Waltham, MA, USA); the DIN (DNA Integrity Number) was determined at Agilent 2200 TapeStation System (Agilent Technologies, Santa Clara, CA, USA) using the DNA genomic assay. The 260/230 and 260/280 ratios were determined by the NanoDrop spectrophotometer to exclude the presence of organic compounds and protein contamination, respectively. Libraries were prepared using SureSelectXT Low Input Target Enrichment System for Illumina Paired-End Multiplexed Sequencing Library protocol (Agilent Technologies, Santa Clara, CA, USA), starting from 200 ng of gDNA and according to the manufacturer’s instructions. Hybridization capture of DNA libraries was performed through a custom RNA probes panel designed to enrich the 9 genes commonly mutated in CHIP. To improve the identification of low allele frequency variants and exclude PCR duplicates, 10-bp unique molecular identifiers (UMIs) were incorporated into each DNA fragment during library preparation. Libraries’ quality and size distribution were assessed on Agilent 2200 TapeStation System (Agilent Technologies, Santa Clara, CA, USA) with the DNA1000 Kit. Before sequencing, the pooled libraries were diluted at a final concentration of 2nM and denatured according to the NextSeq System Denature and Dilute Libraries Guide (Illumina, San Diego, CA, USA); 1% of PhiX DNA was also added to the mix. The pool was sequenced at a loading concentration of 1.8 pM on Illumina NextSeq-500 System in a 2x150 bp paired-end reads format, obtaining an average coverage of 646X. Paired-end demultiplexed reads were produced using bcl2fastq and the quality control was performed using FastQC. The reads were trimmed from the adapters using Trimmomatic ^1^ (v0.39) with a minimum length of 30 bp, and a leading and trailing of 10 bp. The UMI were extracted using UMITools ^2^ and the resulting reads were mapped on the human genome (hg38) using BWA MEM (v0.7.17) ^3^. Sorting and indexing were performed using SAMtools (v1.10) ^4^, while the BAM correction and the metrics collections were obtained using Picard (v2.20.3). Mutect2 from GATK ^5^ (v4.1.2.0) was used for the variant calling of the somatic variants using the *af-only-gnomad.hg38.vcf* as germline-resource. Only the variants that were annotated as “PASS” by FilterMutectCalls were considered somatic variants. The filtered variants were annotated using wANNOVAR ^6^. We considered CHIP-associated variants the ones with Variant Allele Frequency (VAF) of more than 0.02 ^7^ and less than 0.35 to exclude germinal mutations.

1. **RNA sequencing and RT-PCR of aortic valves**

The RNA concentration was measured using the Qubit RNA HS Assay Kit (ThermoFisher Scientific, Waltham, MA, USA) on a Qubit 2.0 Fluorometer (ThermoFisher Scientific, Waltham, MA, USA) while RIN (RNA Integrity Number) was determined at Agilent 2200 TapeStation System (Agilent Technologies, Santa Clara, CA, USA) using the RNA assay. The 260/230 and 260/280 ratios were determined at the NanoDrop spectrophotometer to exclude the presence of organic compounds and protein contamination, respectively. Indexed libraries were prepared starting from 150 ng of purified RNA with TruSeq Stranded Total RNA Library Prep Kit (Illumina, San Diego, CA, USA) according to the manufacturer’s instructions. Libraries’ size distribution was assessed using the Agilent 2200 TapeStation System (Agilent Technologies, Santa Clara, CA, USA) and pooled in equimolar amounts, with a final concentration of 1.5 nM. The pooled samples were sequenced on NovaSeq 6000 System (Illumina, San Diego, CA, USA) in a 2x100 paired-end format at a final concentration of 250 pM. Paired-end demultiplexed reads were produced using bcl2fastq and the quality control was performed using FastQC. The reads were trimmed from the adapters using Cutadapt ^8^ (v3.3) with a minimum length of 20 bp. The produced reads were mapped on the human genome (Gencode v37 primary assembly annotation) using STAR ^9^ (v2.7.8a). The raw counts were obtained using featureCounts on Rsubread (v2.0.1) ^10^. Normalization and Differential Expression analyses were performed on R (v4.0.2) using DeSeq2 (v1.28.1) ^11^. The presence of confoundings was assessed using sva library on R ^12^. Functional analyses of differentially expressed genes were generated through the use of Ingenuity Pathway Analysis ^13^ and only the significant pathways with a p-value < 0.05 were considered.

For RT-PCR validation, RNA concentration was measured by Nanodrop 2000 spectrophotometer (ThermoFisher Scientific, Waltham, MA, USA) then 100 ng of RNA were reverse transcribed in a volume of 25 μl using 250 units of SuperScript III reverse transcriptase and 50 ng of random hexamers on SimpliAmp™ Thermal Cycler (ThermoFisher Scientific, Waltham, MA, USA). 1 μl of the cDNA mixture was used for real-time PCR experiments. Real-time PCR reactions were conducted on StepOnePlus™ Real-Time PCR System (ThermoFisher Scientific, Waltham, MA, USA) using PerfeCta SYBR Green SuperMix with ROX kit (Quanta Biosciences, Beverly, MA, USA) according to the manufacturer’s protocol.

500 nM of the following primers were used: RPL13: forward 5′-GGAGGTGCAGGTCCTGGTGCTT-3′, reverse 5′ CGTACGACCACCACCTTCCGG-3′; IgG1: forward 5′-CTGCAACGTGAATCACAAGC-3′, reverse 5′-GTGGGCATGTGTGAGTTTTG -3′; Igκ: forward 5′- ACGCTGAGCAAAGCAGACTAC-3′, reverse 5′-CCCTGTTGAAGCTCTTTGTG -3′. RPL13 was used as a reference, gene expression levels were expressed as ΔCt.

**Statistics**

All calculations, statistical analyses, and survival curves were produced on R (v4.0.2) with an alpha value set for p < 0.05. Student’s T and Mann–Whitney U tests were used, respectively, to assess statistical differences of normal continuous distributed and not normally distributed data. A Chi-squared test was used to determine the presence of significant differences between the expected frequencies and the observed ones for discrete variables. Missing data were excluded during statistical hypothesis testing. Long-rank test was used to calculate the statistical significance between survival curves. Principal Component Analysis (PCA) plots of previously normalized and log-transformed data were created using the plotPCA function from DESeq2.^11^ Correlations were calculated by Spearman's rank methods and expressed as R Spearman coefficient. CIBERSORTx ^14^ was used in absolute mode with 1,000 permutations to impute gene expression data and provide an estimation of the cell type’s abundances in the samples.

**References**

1 Bolger, A. M., Lohse, M. & Usadel, B. Trimmomatic: a flexible trimmer for Illumina sequence data. *Bioinformatics* **30**, 2114-2120, doi:10.1093/bioinformatics/btu170 (2014).

2 Smith, T., Heger, A. & Sudbery, I. UMI-tools: modeling sequencing errors in Unique Molecular Identifiers to improve quantification accuracy. *Genome Res* **27**, 491-499, doi:10.1101/gr.209601.116 (2017).

3 Li, H. & Durbin, R. Fast and accurate long-read alignment with Burrows-Wheeler transform. *Bioinformatics* **26**, 589-595, doi:10.1093/bioinformatics/btp698 (2010).

4 Li, H. *et al.* The Sequence Alignment/Map format and SAMtools. *Bioinformatics* **25**, 2078-2079, doi:10.1093/bioinformatics/btp352 (2009).

5 Van der Auwera, G. A. *et al.* From FastQ data to high confidence variant calls: the Genome Analysis Toolkit best practices pipeline. *Curr Protoc Bioinformatics* **43**, 11.10.11-11.10.33, doi:10.1002/0471250953.bi1110s43 (2013).

6 Wang, K., Li, M. & Hakonarson, H. ANNOVAR: functional annotation of genetic variants from high-throughput sequencing data. *Nucleic Acids Res* **38**, e164, doi:10.1093/nar/gkq603 (2010).

7 Steensma, D. P. *et al.* Clonal hematopoiesis of indeterminate potential and its distinction from myelodysplastic syndromes. *Blood* **126**, 9-16, doi:10.1182/blood-2015-03-631747 (2015).

8 Kechin, A., Boyarskikh, U., Kel, A. & Filipenko, M. cutPrimers: A New Tool for Accurate Cutting of Primers from Reads of Targeted Next Generation Sequencing. *J Comput Biol* **24**, 1138-1143, doi:10.1089/cmb.2017.0096 (2017).

9 Dobin, A. *et al.* STAR: ultrafast universal RNA-seq aligner. *Bioinformatics* **29**, 15-21, doi:10.1093/bioinformatics/bts635 (2013).

10 Liao, Y., Smyth, G. K. & Shi, W. The R package Rsubread is easier, faster, cheaper and better for alignment and quantification of RNA sequencing reads. *Nucleic Acids Res* **47**, e47, doi:10.1093/nar/gkz114 (2019).

11 Love, M. I., Huber, W. & Anders, S. Moderated estimation of fold change and dispersion for RNA-seq data with DESeq2. *Genome Biol* **15**, 550, doi:10.1186/s13059-014-0550-8 (2014).

12 Parker, H. S., Corrada Bravo, H. & Leek, J. T. Removing batch effects for prediction problems with frozen surrogate variable analysis. *PeerJ* **2**, e561, doi:10.7717/peerj.561 (2014).

13 Krämer, A., Green, J., Pollard, J. & Tugendreich, S. Causal analysis approaches in Ingenuity Pathway Analysis. *Bioinformatics* **30**, 523-530, doi:10.1093/bioinformatics/btt703 (2014).

14 Newman, A. M. *et al.* Determining cell type abundance and expression from bulk tissues with digital cytometry. *Nat Biotechnol* **37**, 773-782, doi:10.1038/s41587-019-0114-2 (2019).
